# Ploidy variation in *Rhododendron* subsection *Maddenia* and its implications for conservation

**DOI:** 10.1093/aobpla/plad016

**Published:** 2023-04-12

**Authors:** Ling Hu, Jennifer A Tate, Susan E Gardiner, Marion MacKay

**Affiliations:** School of Agriculture and Environment, Massey University, Palmerston North 4442, New Zealand; School of Natural Sciences, Massey University, Palmerston North 4442, New Zealand; The New Zealand Institute for Plant and Food Research Limited, Fitzherbert Science Centre, Palmerston North 4472, New Zealand; School of Agriculture and Environment, Massey University, Palmerston North 4442, New Zealand

**Keywords:** Biodiversity conservation, flow cytometry, polyploidy, *Rhododendron*, subsection *Maddenia*, taxonomic complexity

## Abstract

Polyploidy, which is common in plants, can confound taxon recognition and hence conservation assessments. In the taxonomically complex genus *Rhododendron*, 25 % of the over 1,300 taxa are considered under threat and 27 % Near Threatened or Data Deficient, with their taxonomy needing to be resolved urgently. Although ploidy levels of *Rhododendron* taxa range from diploid (2*x*) to dodecaploid (12*x*) according to previous reports, the extent of polyploidy across the genus has not been examined. We first summarized the taxonomic distribution of polyploids in the genus based on the literature. Then as a case study, we estimated ploidy levels of 47 taxa in subsection *Maddenia* (subgenus *Rhododendron*, section *Rhododendron*) using flow cytometry, together with verification of meiotic chromosome counts for representative taxa. The summary of reported ploidy in *Rhododendron* indicates that polyploidy is most common in subgenera *Pentanthera* and *Rhododendron*. In subsection *Maddenia*, all examined taxa are diploids except for the *R. maddenii* complex that shows a high ploidy variation (2–8*x*, 12*x*). We investigated ploidy level of 12 taxa in subsection *Maddenia* for the first time, and estimated genome sizes of two *Rhododendron* species. Knowledge of ploidy levels will inform phylogenetic analysis of unresolved species complexes. Overall, our study of subsection *Maddenia* provides a model for examining multiple issues including taxonomic complexity, ploidy variation and geographic distribution in relation to biodiversity conservation.

## Teaser

Rhododendron is a woody plant genus of over 1,300 species. Although primarily native to the Himalayan mountains, many species are cultivated in botanic gardens around the world because of their spectacular floral diversity. In many plant species, variation in chromosome numbers (ploidy level) is a key aspect of genetic diversity for biodiversity conservation. Our paper examines the taxonomic distribution of reported ploidy levels in *Rhododendron*and investigates the ploidy level of 47 taxa in subsection *Maddenia*(~65 taxa). Using flow cytometry with chromosome count validation, we report ploidy of 12 taxa for the first time, and verify that taxa in subsection *Maddenia*are all diploids except for the *R. maddenii*complex. Our future study on species geography and phylogenetics will help to understand the rich diversity and resolve the complex taxonomy of subsection *Maddenia*and eventually of the genus *Rhododendron*.

## Introduction

Polyploidization, or whole-genome duplication (WGD), generates organisms containing multiple sets of chromosomes. This major mechanism of plant speciation results from either intraspecific genome duplication (autopolyploidy) or hybridization between different species and chromosome doubling (allopolyploidy) (Stebbins 1947; Van de Peer *et al.* 2017). Fertile polyploids can become new species when strong reproductive incompatibilities and distinct phenotypic differences occur, differentiating them from their diploid progenitors. Polyploidization, accompanied by corresponding morphological differences, has been considered as a characteristic for recognition of species which form conservation units (Soltis *et al.* 2007, 2010; Laport and Ng 2017). Due to multiple copies of genes facilitating adaptive processes, polyploids may be more successful at adapting to new environments (Comai 2005; Van de Peer *et al.* 2017, 2021). As ploidy variation can be associated with regional biodiversity, it should be included in diversity measurements (e.g. phenotypic, inter- and/or intraspecific diversity) for the consideration of conservation, especially in temperate regions where polyploidization is frequently observed (Comai 2005; Laport and Ng 2017; Rice *et al.* 2019).

*Rhododendron* L. (Ericaceae) is a megadiverse genus with more than 1,300 taxa [species, subspecies (ssp.) and varieties (var.)] that typically grow in temperate regions (Gibbs *et al.* 2011). The wild distribution of *Rhododendron* covers a geographic range from the centres of diversity in the south-eastern Himalayas and Malay Archipelago to North America, Europe and northern Australia (Gibbs *et al.* 2011; Argent 2015; MacKay *et al.* 2018; Shrestha *et al.* 2018). The Himalayan region is characterized by a rich biotic assembly (Ming and Fang 1979; Yan *et al.* 2015; Hughes 2017; Shrestha *et al.* 2018), where polyploids are likely to diversify under environmental stress (Rice *et al.* 2019; Van de Peer *et al.* 2021). Extensive hybridization, due to weak reproductive barriers within *Rhododendron,* is also a possible cause of rapid speciation (Frodin 2004; Zhang *et al.* 2007; Zha *et al.* 2008; Soltis and Soltis 2009; Ma *et al.* 2010; Qiu *et al.* 2020). However, hybridization and polyploidy and their influence on speciation rate in *Rhododendron* are still under investigation (Milne *et al.* 2010; Schwery *et al.* 2015; Shrestha *et al.* 2018; Khan *et al.* 2021). Taxa produced from introgression of sympatric species often show morphological similarity to their parents, making correct taxon identification challenging (Darlington *et al.* 1955; Milne *et al.* 2010; Zhang *et al.* 2020).

Effective decisions and strategies for species conservation require distinct taxonomy to assess the risk of extinction of species. It has been reported that 25 % of *Rhododendron* taxa are under threat (Critically Endangered, Endangered and Vulnerable), and 27 % Near Threatened or Data Deficient (MacKay *et al.* 2018). However, problems of taxon identification due to taxonomic complexity and lack of cytogenetic knowledge of particular accessions still need to be resolved to inform conservation strategies (Mehra 1976; Jones *et al.* 2007; Gibbs *et al.* 2011; Mao *et al.* 2017; Khan *et al.* 2021). Molecular phylogenetic techniques can assist to resolve taxonomic uncertainties (Gibbs *et al.* 2011; Gardiner *et al.* 2019). However, the presence of polyploids can confound analyses due to duplicated genomes that are often derived from multiple species (Yan *et al.* 2015; Rothfels 2021). For plant genera that include polyploids, such as *Rhododendron*, any investigation of phylogeny should be preceded by an examination of ploidy levels in the taxa under consideration (Khan *et al.* 2021).

Cytological studies of *Rhododendron* species began in 1930 (Bowers 1930; Sax 1930), with the most extensive and genus-wide chromosome counts reported in the 1950s (Ammal *et al.* 1950; Darlington *et al.* 1955). In *Rhododendron* species, mitotic chromosomes in the root tips are notably small and difficult to distinguish under the microscope (Jones *et al.* 2007; Zaytseva *et al.* 2018). This difficulty may increase for counting the multiple sets of chromosomes in polyploids (Comai 2005; Van de Peer *et al.* 2017). Meiotic chromosome number can be more easily determined by counting haploid chromosomes in pollen mother cells (PMCs) (Windham *et al.* 2020), but little information is available on optimal bud harvest time for meiotic observation in *Rhododendron*. In contrast, flow cytometry (FCM) saves time by enabling rapid determination of nuclear DNA content (genome size) for a large number of samples (Doležel *et al.* 1998; De *et al.* 2010; Zaytseva *et al.* 2018). Apart from the measurement of genome size (Bou Dagher-Kharrat *et al.* 2013; Khan *et al.* 2021; Choi *et al.* 2022), FCM has been applied to estimate *Rhododendron* ploidy levels in several studies (Doležel *et al.* 1998; De Schepper *et al.* 2001; Jones *et al.* 2007; Zhou *et al.* 2008; Zaytseva *et al.* 2018; Khan *et al.* 2021). Cytological studies and FCM generally require access to living material. However, this can be hindered due to difficulties in accessing remote *Rhododendron* habitats or living accessions on sites of *ex situ* collections internationally. For FCM at least, the use of dehydrated leaf tissues has proven to be reliable for ploidy estimation in other species (Suda and Trávníček 2006; Tomaszewska *et al.* 2021), but this approach has not yet been tested on *Rhododendron*.

*Rhododendron* is taxonomically complex, divided into nine subgenera (if considering *Vireya* as a subgenus) with further sections and subsections of varying sizes (Chamberlain *et al.* 1996; Frodin 2004; Argent 2015). Davidian (1982) systematically described the morphology of *Rhododendron*. Chamberlain *et al.* (1996) ‘lumped’ a number of previously recognised species as synonyms, which is considered the most comprehensive reference for the taxon checklist to date. In this study, we initially consider the whole genus, and then focus on subsection (ss.) *Maddenia* (subgenus *Rhododendron*, section *Rhododendron*) as a case study. Due to the complex taxonomy and continuous morphological variation within ss. *Maddenia*, many questions remain about species boundaries, which is identified as a general problem in the genus (Chamberlain *et al.* 1996; Cullen 2005; Gibbs *et al.* 2011; Donald 2012; MacKay 2018; Jamieson 2021). Ss. *Maddenia* is the largest among all the subsections in subgenus *Rhododendron* (if the vireyas are treated as subgenus *Vireya* rather than the broad section *Schistanthe* under subgenus *Rhododendron*), and several new species (Mao and Bhaumik 2015; Mao *et al.* 2017; Chang *et al.* 2021; Rushforth *et al.* 2022) have been published since Chamberlain *et al.* (1996). With ‘lumping’ species as synonyms and/or changes in placement of species over time, the number of accepted species in ss. *Maddenia* varies in treatments by different authors. In *The Rhododendron Species*, Davidian (1982) used the concept of ‘series’ rather than ‘subsection’ and listed a total of 56 species in the two series Ciliatum and Maddenii. In *The Genus Rhododendron: Its classification & synonymy* that we are following as the major reference for taxonomic classification, Chamberlain *et al.* (1996) listed 52 species (57 taxa) in ss. *Maddenia*. Cox and Cox (1997) included 31 species of ss. *Maddenia* in *The Encyclopedia of Rhododendron Species*, while Khan *et al.* (2021) defined 56 species in the phylogenetic study. In ss. *Maddenia*, the *R. maddenii* complex is especially problematic, with 12 taxa placed in synonymy under the two subspecies of *R. maddenii* (*R. maddenii* ssp. *maddenii* and *R. maddenii* ssp. *crassum*) (Chamberlain *et al.* 1996). Previous studies also found exceptional occurrence of polyploids (tetraploid, hexaploid, octoploid and dodecaploid) in *R. maddenii*, which raises questions on the uniqueness of this species complex and patterns of species diversification in ss. *Maddenia* (Ammal *et al.* 1950; Cubey 2003; Khan *et al.* 2021).

Previous phylogenetic studies indicate that ss. *Maddenia* may not be monophyletic (Donald 2012; Shrestha *et al.* 2018; Khan *et al.* 2021). However, two of these studies encompassed the whole genus, with few species included from ss. *Maddenia* (Shrestha *et al.* 2018; Khan *et al.* 2021), while Donald (2012) only considered the yellow-flowered species that include part of ss. *Maddenia* species. The species coverage as well as number of molecular markers applied in previous studies were limited, which does not provide adequate evidence on the relationships between ss. *Maddenia* and other possibly related species or subsections.

**Figure 1.**
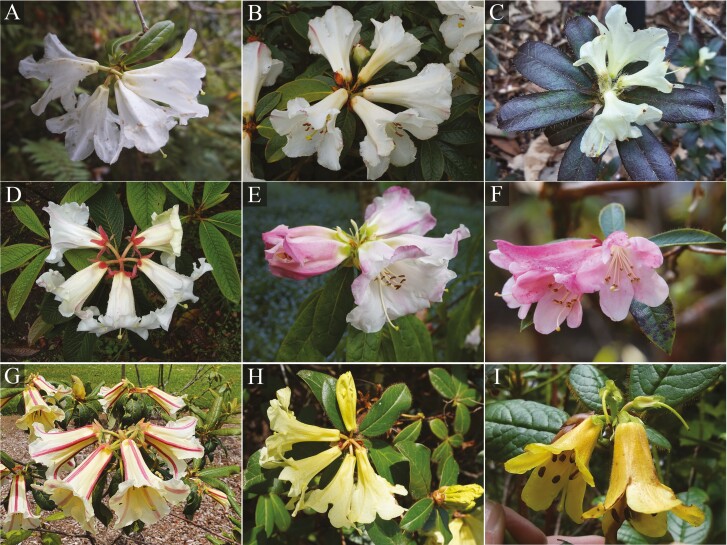
Representative taxa of *Rhododendron* subsection *Maddenia* and their conservation status. (A) *R. coxianum*, Critically Endangered. (B) *R. maddenii* ssp. *crassum*, Least Concern. (C) *R. fletcherianum*, Endangered. (D) *R. nuttallii*, Near Threatened. (E) *R. lindleyi*, Least Concern. (F) *R. formosum* var. *formosum*, Critically Endangered. (G) *R. dalhousiae* var. *rhabdotum*, Vulnerable. (H) *R. burmanicum*, Least Concern. (I) *R. valentinianum* var. *oblongilobatum*, Near Threatened. Conservation status of all taxa was published at the global level (Gibbs *et al.* 2011), except for *R. maddenii* ssp. *crassum* at the national level in China (MEP–CAS 2013).

Subsection *Maddenia* includes rhododendrons that are all lepidote (scaly), originating from the Himalayan region through to southern China and northern Vietnam. The taxa not only present great morphological diversity (Fig. 1) but also possess unique horticultural value because of their scented flowers and high resistance to thrips (Cullen 1980; MacKay *et al.* 2018; Jamieson 2021). According to conservation assessments for 51 ss. *Maddenia* taxa, 33 were placed in either a threatened category or Data Deficient (Gibbs *et al.* 2011; MacKay *et al.* 2018; Chang *et al.* 2021). Due to the variable taxonomy and species definitions derived from traditional morphology, conservation assessments and subsequent conservation action are subject to debate (Cubey 2003; Gibbs *et al.* 2011; Donald 2012; Jamieson 2021).

In this study, we collected leaf samples from living accessions to examine the ploidy variation in ss. *Maddenia*. Our methodology involved (i) reviewing the literature to establish a comprehensive dataset of ploidy levels across *Rhododendron* taxa; (ii) investigating the basic cytogenetics of ss. *Maddenia* by estimating ploidy, especially of the previously reported polyploid taxa in the *R. maddenii* complex and of taxa not previously studied. Flow cytometry was used for ploidy estimation, and when possible, confirmed with meiotic chromosome counts from living material. The resulting data will not only inform our future molecular phylogenetic studies but also assist in developing an understanding of the speciation and ecological features of ss. *Maddenia* for conservation management.

## Materials and Methods

### Literature review of ploidy in *Rhododendron*

Prior knowledge on ploidy levels of *Rhododendron* taxa was compiled in a spreadsheet, with data from chromosome counting (2*n*) and flow cytometry (*x*) both included but listed separately [see Supporting Information—Table S1]. Chromosome data were compiled from the Chromosome Counts Database (CCDB, Rice *et al.* 2015), Index to Plant Chromosome Numbers (IPCN, Goldblatt and Johnson 1979) and literature not covered by these two databases (Sax 1930; Ammal *et al.* 1950; Li 1957; Cubey 2003; Contreras *et al.* 2007). FCM ploidy data were collected from previous reports (De Schepper *et al.* 2001; Jones *et al.* 2007; De *et al.* 2010; Khan *et al.* 2021; Choi *et al.* 2022). Taxonomic classification (subgenus, section, subsection) followed Chamberlain *et al.* (1996), except that the vireya species were treated as subgenus *Vireya* following Argent (2015) (subgenus *Vireya* may be treated as the broad section *Schistanthe* under subgenus *Rhododendron* as in recent studies). Taxa together with their synonyms were indexed according to Chamberlain *et al.* (1996) except for those in subgenus *Vireya* (Argent 2015). We integrated the classification for the convenience of indexing taxon names and summarizing ploidy levels, as these two publications are the most comprehensive and latest references for the respective groups. Irrespective of the positioning of the Indomalesian species as subgenus *Vireya* or the broad section *Schistanthe* (Goetsch *et al.* 2011; Shrestha *et al.* 2018; Khan *et al.* 2021), the group of species involved is still largely as described by Argent (2015). Taxa were recorded as polyploids if polyploidy was reported from either chromosome counting or flow cytometry. Reported ploidy, chromosome number and proportion of polyploid taxa were summarized for each taxonomic group.

### Plant material collection and taxon identification

For flow cytometry, 263 accessions of ss. *Maddenia* taxa were selected from botanic and/or private gardens and nurseries in New Zealand (164) along with accessions from the Royal Botanic Garden Edinburgh (RBGE, 25), UK, and the Rhododendron Species Botanical Garden (RSBG, 74), USA [see Supporting Information—Table S2]. Accessions from RBGE and RSBG are cultivated material of wild origin, while the New Zealand accessions consist of material from wild and horticultural sources. Herbarium specimens for New Zealand accessions are deposited in the Dame Ella Campbell Herbarium (MPN) at Massey University, Palmerston North, New Zealand. Living plants of the accessions in RBGE and RSBG are accessible at the corresponding organizations [see Supporting Information—Table S2].

All taxa of ss. *Maddenia* that have been previously reported for ploidy, except for *R. vanderbiltianum* (Atkinson *et al.* 2000) and *R. yungchangense* (Cubey 2003), were included in the present study. Further to the taxa listed by Chamberlain *et al.* (1996), four taxa were separated from species complexes and analysed as distinct entities: *R. iteophyllum* (A Mao *et al.* 2017), *R. sinonuttallii* (Gibbs *et al.* 2011), *R. taronense* (Gibbs *et al.* 2011) and *R. valentinioides* (ined.) (Gibbs *et al.* 2011; Donald 2012). Three new species published post-1996 were examined: *R. pseudomaddenii* (Mao and Bhaumik 2015), *R. leptocladon* (Rushforth and Nguyen 2019) and *R. kuomeianum* (Chang *et al.* 2021). *R. vanderbiltianum* was also included because of its suggested placement in this subsection (Argent *et al.* 2008; Donald 2012; MacKay *et al.* 2018). Individual accessions from New Zealand collections were identified following Davidian (1982), based on herbarium specimens and photographs taken in the field.

While fresh leaves were collected locally in New Zealand, overseas samples were silica-gel dried and imported from RBGE and RSBG to New Zealand. In both cases, fully expanded young leaves were routinely sampled for flow cytometry, although sometimes leaves from the previous season’s growth were used when young leaves were unavailable. Fresh samples from the New Zealand sites were chilled and shipped overnight to the laboratory for FCM ploidy estimation. For some local accessions, dehydrated leaf samples from herbarium specimens were used, as fresh or silica gel-dried leaf materials were unavailable at the time of the FCM experiment [see Supporting Information—Table S2]. A subset of samples was replicated to verify the consistency of ploidy results for the same accession using fresh leaf vs. silica gel-dried leaf vs. dried leaf from an herbarium specimen.

Leaf tissue collected from *R. fortunei* was routinely used as the internal diploid standard for flow cytometry. When fresh leaves of *R. fortunei* were unexpectedly unavailable, *R. parryae* was used as the internal standard. Both species were previously reported as diploids (Ammal *et al.* 1950; Jones *et al.* 2007) and available as living plants for our sampling. Analysis of a subset of samples was repeated to verify the consistency of ploidy results for the same accession using either of the diploid standards. Genome sizes of these two *Rhododendron* species were measured [see Supporting Information—Table S2], using *Pisum sativum* L. (2C = 8.8 pg) and *Zea mays* L. (2C = 5.33 pg) as standards.

### Flow cytometry preparation and analysis

Cell nuclei suspensions from leaf tissue were prepared for flow cytometry following Doležel *et al.* (2007) with minor modifications. Both fresh and dehydrated leaf samples were processed using the same protocol at Manaaki Whenua—Landcare Research (Lincoln, New Zealand). Approximately 1 cm^2^ of leaf tissue of each sample was co-chopped with the diploid standard using a sharp razor blade in approximately 1 mL ice-cold Otto I buffer in a Petri dish, then left to incubate for 1–2 min at room temperature. The homogenate was filtered into a sample tube through a 20-µM nylon mesh to remove large particles. Next, DAPI (4ʹ6-diamidino-2-phenylindole) stain, prepared with Otto II solution, was added to the sample tube at 4 µg/mL. The samples were run on a Partec PAII flow cytometer, using a 375-nm UV laser and FloMax software, until the particle count was at least 5000. Histogram peaks were manually gated for all samples. The relative fluorescence values of the peak positions of DAPI-stained nuclei (Mean-x) and the coefficient of variation (CV-x %) of the RN1/RN2 peak were evaluated. Where there was any uncertainty to gate a peak, the sample was run alone, then co-chopped with the diploid standard to confirm the peak of the standard. Data were transferred to an Excel spreadsheet from which the FCM ploidy (*x*) was calculated [see Supporting Information—Table S2]. Genome size measurements of the two diploid standards of *Rhododendron* were performed using the same protocol and reagents, except that 10 µg/mL propidium iodide was used as the stain, and samples were run on a Partec CyFlow Space with a blue laser at 488 nm. Each plant sampled was measured sequentially in triplicate using leaves harvested on the same day, from which the average 2C value (pg) was calculated for the genome size.

### Validation of FCM ploidy with meiotic chromosome counts

Chromosome counts were performed on a sub-sample of six accessions to validate the estimated ploidy levels from flow cytometry. Developing flower buds were harvested on sunny mornings in a local garden in New Zealand, mostly between 0730 and 0930 hours. Prior to the harvest, a series of observations were made to determine the correct stage of the meiotically dividing PMCs. Serial sampling of flower buds was performed during the growth of anthers. The outer layers of bud scales were removed before the buds were immersed in fixative (1 part of glacial acetic acid to 3 parts of absolute ethanol) for at least 24 h. Under a stereo microscope (Leica MZ9.5), young anthers were removed from the buds, mashed and stained with 1 % acetocarmine. After removing the debris, slides with a coverslip were heated to ~50 °C for ~30 s and set aside for 1.5–2 h for deeper staining, followed by the final ‘squash’ onto the slide. The meiotic chromosomes of PMCs were observed under a compound microscope (Leica DM500) and images were captured with 100× objective under oil immersion.

## Results

### Taxonomic distribution of polyploidy in *Rhododendron*

**Figure 2.**
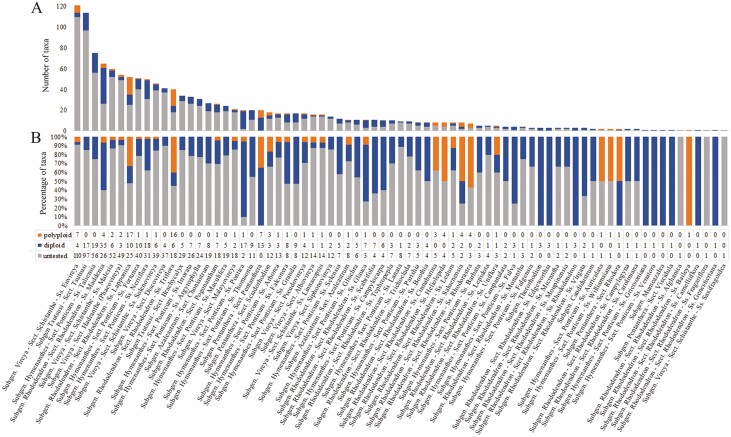
Extent of polyploidy in the reported taxa of *Rhododendron*. (A) Number of taxa. (B) Percentage of taxa. Bars are parallel in both graphs, following the descending order of the number of taxa (A) in each taxonomic group. Each bar represents the reported polyploid taxa (orange), diploid taxa (blue) and untested taxa (grey) in the corresponding taxonomic group (subgenus, section, or subsection), with only the lowest levels listed. Taxa reported include species, subspecies and botanical varieties. A taxon was counted as a polyploid when there was an occurrence from either chromosome counting or flow cytometry. The bottom table shows taxon numbers of the reported ploidy levels in each group. Corresponding to Table 1, number of ‘diploid’ taxa = number of (‘tested taxa’ – ‘polyploid taxa’), while number of ‘untested’ taxa = number of (‘all taxa’ – ‘tested taxa’). Ploidy data were compiled from literature. Details are shown in Supporting Information—Table S1. See online for colour figure.

Our summary of ploidy levels from existing databases and the literature demonstrated that polyploidy occurs in five of the nine subgenera of *Rhododendron* (considering *Vireya* as a subgenus) (Table 1; [see Supporting Information—Table S1]). All subgenera have representative taxa investigated for ploidy levels. Of the total 424 taxa reported, 332 are diploids and 92 are polyploids. Subgenus *Rhododendron* contains the largest number of polyploid taxa, followed by subgenera *Vireya*, *Pentanthera*, *Hymenanthes* and *Azaleastrum* (in the descending order of the number of polyploid taxa reported). Sections *Rhododendron* (in subgenus *Rhododendron*) and *Schistanthe* (in subgenus *Vireya*) have the most polyploid taxa at the sectional level. Notably, subsections *Lapponica* and *Triflora* in section *Rhododendron* contain the most polyploid taxa at subsection level. In six subsections, all tested taxa were reported as polyploids, including subsections *Auriculata*, *Baileya*, *Cinnabarina*, *Heliolepida*, *Rhodorastra* and *Williamsiana*. Despite 424 taxa with chromosome counts or ploidy estimates reported to date, there remain some 952 *Rhododendron* taxa for which ploidy has not been studied (Fig. 2). Information on total taxa at a taxonomic level can be found in Table 1 and Supporting Information—Table S1.

**Table 1.** Taxonomic distribution of polyploidy in *Rhododendron.* Values in parentheses indicate the number of (polyploid taxa)/(tested taxa)/(all taxa) in the corresponding groups. Ploidy data were combined from flow cytometry and chromosome counts. Taxa were counted as polyploids when there was an occurrence, even if diploids were also found. E.g., of the 38 taxa in subgenus *Azaleastrum*, 11 have been examined for ploidy, in which one taxon was reported with occurrence of polyploids. Taxonomic classification of genus *Rhododendron* is according to Chamberlain *et al.* (1996) and Argent (2015). Non-vireya taxon names were indexed following Chamberlain *et al.* (1996) while vireyas (subgenus *Vireya*) following Argent (2015). Other taxonomies may treat the subgenus *Vireya* in this table as the broad section *Schistanthe* under subgenus *Rhododendron* (Goetsch *et al.* 2011; Shrestha *et al.* 2018; Khan *et al.* 2021). *Chromosome counts of taxa in subsection *Saluenensia* by Cubey (2003) are not included due to inaccessibility of data. Reported ploidy data are in Supporting Information—Table S1

Subgenus	Section	Subsection
*Azaleastrum* (1/11/38; 2 sections)	*Azaleastrum* (1/3/11) *Choniastrum* (0/8/27)	
*Candidastrum* (0/1/2)		
*Hymenanthes* (7/149/427; 1 section)	*Ponticum* (7/149/427; 24 subsections)	*Arborea* (1/4/17), *Argyrophylla* (0/7/31), *Auriculata* (1/1/2), *Barbata* (0/2/5), *Campanulata* (0/2/4), *Campylocarpa* (0/6/10), *Falconera* (1/9/17), *Fortunea* (1/11/51), *Fulgensia* (0/1/3), *Fulva* (0/3/4), *Glischra* (0/5/11), *Grandia* (0/9/17), *Griersoniana* (0/1/1), *Irrorata* (0/7/33), *Lanata* (0/1/9), *Maculifera* (0/5/24), *Neriiflora* (1/19/50), *Parishia* (0/3/8), *Pontica* (1/18/20), *Selensia* (0/5/12), *Taliensia* (0/19/75), *Thomsonia* (0/9/20), *Venatora* (0/1/1), *Williamsiana* (1/1/2)
*Mumeazalea* (0/1/1)		
*Pentanthera* (11/29/41; 4 sections)	*Pentanthera* (7/20/20)	
	*Rhodora* (1/2/2)	
	*Sciadorhodion* (3/6/18)	
	*Viscidula* (0/1/1)	
*Therorhodion* (0/3/3)		
*Tsutsusi* (0/22/148; 2 sections)	*Brachycalyx* (0/5/34)	
	*Tsutsusi* (0/17/114)	
*Rhododendron* (57/159/309; 2 sections)	*Pogonanthum* (1/8/26)	
	*Rhododendron* (56/151/283; 28 subsections)	*Afghanica* (0/0/1), *Baileya* (1/1/1), *Boothia* (0/4/8), *Camelliiflora* (0/1/1), *Campylogyna* (0/1/2), *Caroliniana* (0/1/2), *Cinnabarina* (3/3/8), *Edgeworthia* (0/3/3), *Fragariflora* (0/0/1), *Genestieriana* (0/1/1), *Glauca* (1/8/11), *Heliolepida* (4/4/8), *Lapponica* (17/27/52), *Ledum* (1/3/8), *Lepidota* (0/1/5), *Maddenia* (4/39/65), *Micrantha* (0/1/3), *Monantha* (0/1/4), *Moupinensia* (0/1/3), *Rhododendron* (0/3/3), *Rhodorastra* (4/4/7), *Saluenensia* (4/6/8)^*^, *Scabrifolia* (0/7/11), *Tephropepla* (0/3/10), *Trichoclada* (0/2/9), *Triflora* (16/22/40), *Uniflora* (1/2/5), *Virgata* (0/2/3)
*Vireya* (16/49/407; 7 sections)	*Albovireya* (1/2/16) *Discovireya* (0/4/41)	
	*Hadranthe* (*Phaeovireya*) (2/5/54)	
	*Malayovireya* (1/3/21)	
	*Pseudovireya* (1/5/17)	
	*Schistanthe* (11/28/244; 5 subsections)	*Euvireya* (7/11/121), *Linnaeopsis* (1/2/16), *Malesia* (2/8/60), *Saxifragoidea* (0/0/1), *Solenovireya* (1/7/46)
	*Siphonovireya* (0/2/14)	

Ploidy level is highly varied in *Rhododendron* taxa, including 2*x* (387 taxa), 3*x* (9), 4*x* (76), 5*x* (1), 6*x* (25), 8*x* (4) and 12*x* (1) [see Supporting Information—Table S1]. Only diploids have been reported in four of the nine subgenera and several sections or subsections of the other five subgenera (Table 1; Fig. 2). Intraspecific ploidy variation has been observed in a total of 55 taxa in subgenera *Hymenanthes* (6), *Pentanthera* (7), *Rhododendron* (35) and *Vireya* (7). The highest number of intraspecific ploidy levels was revealed in the *R. maddenii* complex, with four levels in both *R. maddenii* ssp. *maddenii* and *R. maddenii* ssp. *crassum*. Over the genus, a discrepancy occurs that polyploidy was reported only from either chromosome counting or flow cytometry when both methods have been used to study the same taxa, which was observed in 22 taxa among the total 424 reported [see Supporting Information—Table S1]. For example, polyploids were identified from only flow cytometry in subgenus *Vireya* and ss. *Rhodorastra* in subgenus *Rhododendron*. In such cases, these taxa were recorded as polyploids for our examination of frequency of polyploidy in *Rhododendron* groups.

### Ploidy levels of taxa in subsection *Maddenia*

#### Ploidy estimation using flow cytometry

Using flow cytometry, we assigned ploidy level to 263 accessions covering 47 of the c.65 taxa in ss. *Maddenia.* These include 201 accessions of 45 taxa outside the *R. maddenii* complex and 62 accessions within the complex (*R. maddenii* ssp. *maddenii* and *R. maddenii* ssp. *crassum* with their synonyms) (Table 2; [see Supporting Information—Table S2]). All taxa outside the *R. maddenii* complex were diploids, with 43 taxa reported using flow cytometry for the first time, among which 12 taxa had no previously reported ploidy data. Polyploids were only identified within the two subspecies of *R. maddenii*, and there were seven diploids as well as 55 polyploid accessions. At least one wild accession was tested for each of 43 taxa (a total of 135 wild accessions examined).

**Table 2.** Ploidy of subsection *Maddenia* in the present study in comparison to published ploidy levels compiled from the literature. Diploids are 2*n* = 2*x* = 26. ^1^Identified taxa in alphabetical order. Taxon list follows Chamberlain *et al.* (1996). ^2^‘Yes’ indicates the first report of flow cytometry ploidy estimation from the present study, with chromosome counts reported previously. ‘*’ indicates the first ploidy report from the present study. ‘/’ indicates the accession was not considered as a distinct species, as it is an affinity or synonym and shown in the parentheses. Ploidy data in Supporting Information—Table S2. ^3^Identification of accessions undetermined for subspecific taxonomy in the complex, based on morphology, thus listed as the species. ^4^Considered as a distinct species according to literature (Fang *et al.* 2005; Gibbs *et al.* 2011; Donald 2012; Cox 2013; Mao and Bhaumik 2015; Mao *et al.* 2017; Chang *et al.* 2021). ^#^Reported as a polyploid from a single accession (Cubey 2003; Khan *et al.* 2021)

Ss. *Maddenia* taxon^1^	Present study			Reported 2*n* or *x*
No. of acc. (wild)	FCM ploidy (no. of acc.)	First report here^2^
*R. burmanicum*	8 (1)	2*x*	Yes	26
*R. carneum*	3 (0)	2*x*	No	26; 4*x*^#^
*R. changii*	1 (1)	2*x*	Yes^*^	
*R. ciliatum*	9 (4)	2*x*	Yes	26
*R. ciliicalyx*	4 (2)	2*x*	Yes	26
*R. ciliipes*	3 (2)	2*x*	Yes^*^	
*R. coxianum*	1 (1)	2*x*	Yes	26
*R. crenulatum*	1 (1)	2*x*	Yes	26
*R. cuffeanum*	1 (1)	2*x*	Yes	26
*R. dalhousiae* var. *dalhousiae*	6 (4)	2*x*	No	26; 2*x*
var. *rhabdotum*	5 (1)	2*x*	Yes	26
*R. dendricola*	8 (4)	2*x*	Yes	26
*R. excellens*	8 (7)	2*x*	Yes	26
*R. fletcherianum*	3 (2)	2*x*	Yes	26
*R. fleuryi*	1 (1)	2*x*	Yes	26
*R. formosum* ^3^	5 (3)	2*x*	/	/
var. *formosum* (*R.formosum*)	10 (1)	2*x*	Yes	26
(*R. iteophyllum*^4^)	3 (0)	2*x*	Yes	c.26
var*. inaequale*	2 (1)	2*x*	Yes	26
(*R.* aff. *formosum*)	1 (1)	2*x*	/	/
*R. goreri*	2 (2)	2*x*	Yes^*^	
*R. horlickianum*	5 (2)	2*x*	Yes	26
*R. johnstoneanum*	8 (3)	2*x*	Yes	26
*R. kiangsiense*	1 (1)	2*x*	Yes^*^	
*R. kuomeianum* ^4^	1 (1)	2*x*	Yes^*^	
*R. leptocladon* ^4^	3 (3)	2*x*	Yes	26
*R. levinei*	2 (2)	2*x*	Yes^*^	
(*R.* aff. *levinei*)	1 (1)	2*x*	/	/
*R. liliiflorum*	5 (5)	2*x*	Yes	26
*R. lindleyi*	11 (3)	2*x*	No	26; 2*x*
*R. ludwigianum*	4 (3)	2*x*	Yes	26
*R. lyi*	5 (3)	2*x*	Yes	26
(*R.* aff. *lyi*)	1 (1)	2x	/	/
*R. maddenii* ^3^	6 (6)	2*x* (1), 6*x* (4), 8*x* (1)	/	26, 52, 78, 104; 2*x*, 6*x*, 8*x*
*R. maddenii* ssp. *maddenii*	8 (6)	4–6*x*? (1), 5*x* (1), 6*x* (4), 6–7*x*? (1), 8*x* (1)	No	26 (only in synonym *R. calophyllum*), 52, 78; 6*x*
(*R. maddenii*)	20 (5)	2*x* (1), 6*x* (14), 7*x* (2), 8*x* (3)	/	/
(*R. brachysiphon*)	3 (0)	6*x* (3)	/	/
(*R. polyandrum*)	2 (2)	6*x* (2)	/	/
*R. maddenii* ssp. *crassum*	13 (11)	2*x* (5), 5–6*x*? (1), 8*x* (7)	No	52, 78, 104; 6*x*, 8*x*
(*R. crassum*)	6 (1)	5*x*? (1), 8*x* (3), 7*x* (2)	/	/
(*R. manipurense*)	4 (1)	6*x* (1), 8*x* (3)	/	78, 156
*R. megacalyx*	5 (2)	2*x*	Yes	26
*R. nuttallii*	9 (6)	2*x*	Yes	26
(var. *stellatum*)	2 (0)	2*x*	/	/
*R. pachypodum*	4 (2)	2*x*	Yes	26
(*R. supranubium*)	3 (0)	2*x*	/	26
*R. parryae*	3 (1)	2*x*	Yes	26
*R. pseudociliipes*	3 (3)	2*x*	Yes^*^	
*R. pseudomaddenii* ^4^	1 (1)	2*x*	Yes^*^	
*R. roseatum*	1 (1)	2*x*	Yes	26
*R. scopulorum*	6 (4)	2*x*	Yes	26
*R. sinonuttallii* ^4^	2 (0)	2*x*	Yes	26
*R. surasianum*	1 (1)	2*x*	Yes^*^	
*R. taggianum*	5 (1)	2*x*	Yes	26
*R. taronense* ^4^	3 (0)	2*x*	Yes	c.104^#^
*R. valentinianum* var. *oblongilobatum*	3 (2)	2*x*	Yes^*^	
*R.* aff. *valentinianum*	1 (0)	2*x*	/	/
*R. valentinioides* (ined.)^4^	1 (1)	2*x*	Yes^*^	
[*R.* aff. *valentinioides* (ined.)]	1 (1)	2*x*	/	/
*R. veitchianum*	6 (5)	2*x*	Yes	26
(*R. cubittii*)	6 (1)	2*x*	/	26
*R. walongense*	2 (2)	2*x*	Yes	26
*R. wumingense*	1 (1)	2*x*	Yes^*^	

When 20 accessions that had been first evaluated from fresh leaves were replicated using dehydrated samples (silica gel-dried or herbarium specimen), ploidy of the seven diploid taxa was consistent, regardless of how the sample was dried. For silica gel-dried samples, FCM ploidy was generally the same as from fresh leaves (Table 3 and see Supporting Information—Table S3). Among the 13 tested polyploids from the *R. maddenii* complex, nine samples showed reproducible ploidy levels, while ploidy for four accessions was not certainly determined. However, when herbarium specimens were used for the same accessions, the ploidy level was the same as using fresh leaves for only three of the polyploid accessions. Polyploidy was not determined for three accessions, or one level lower than ploidy estimated from fresh leaves for five accessions (Table 3).

**Table 3.** Flow cytometry estimates of ploidy level for samples from fresh and dehydrated leaves (silica gel-dried or air-dried herbarium sample). ^1^All samples were analysed using *R. fortunei* as the diploid standard. FCM histograms of accessions with inconsistent ploidy in different runs are shown in Supporting Information—Table S3

Ss. *Maddenia* taxon	Acc. ID	FCM ploidy^1^	Leaf sample
*R. burmanicum*	OM55	2*x*	Fresh
		2*x*	Silica gel-dried
		2*x*	Herbarium
*R. ciliicalyx*	OM06	2*x*	Fresh
		2*x*	Silica gel-dried
		2*x*	Herbarium
*R. dalhousiae* var. *dalhousiae*	OM32	2*x*	Fresh
		2*x*	Silica gel-dried
		2*x*	Herbarium
*R. excellens*	OM34	2*x*	Fresh
		2*x*	Silica gel-dried
		Aneuploid < 2*x*?	Herbarium
*R. formosum* var. *formosum*	OM43	2*x*	Fresh
		2*x*	Silica gel-dried
		2*x*	Herbarium
*R. maddenii* ssp. *crassum*	OM17	8*x*	Fresh
		8*x*	Silica gel-dried
		8*x*	Herbarium
	OM47	8*x*	Fresh
		8*x*	Silica gel-dried
		2*x*	Herbarium
*R. maddenii* ssp. *maddenii*	OM02	6*x*	Fresh
		6*x*	Silica gel-dried
		5*x*	Herbarium
	OM11	6*x*	Fresh
		6*x*	Silica gel-dried
		6*x*	Herbarium
	OM14	8*x*	Fresh
		8*x*	Silica gel-dried
		2*x*?/8*x*?	Herbarium
	OM18	6*x*	Fresh
		6*x*	Silica gel-dried
		5*x*	Herbarium
	OM20	6*x*	Fresh
		6*x*	Silica gel-dried
		5*x*	Herbarium
	OM46	6*x*	Fresh
		5₋6*x*?	Silica gel-dried
		5*x*	Herbarium
	OM48	7*x*	Fresh
		2*x*?/7*x*?	Silica gel-dried
		6₋7*x*	Herbarium
	OM49	6*x*	Fresh
		5₋6*x*?	Silica gel-dried
		6*x*	Herbarium
	OM54	6*x*	Fresh
		5₋6*x*?	Silica gel-dried
		5*x*	Herbarium
	OM56	6*x*	Fresh
		6*x*	Silica gel-dried
		2*x*	Herbarium
	OM58	6*x*	Fresh
		6*x*	Silica gel-dried
		6*x*	Herbarium
*R. sinonuttallii*	OM57	2*x*	Fresh
		2*x*	Silica gel-dried
		2*x*	Herbarium
*R. taronense*	OM40	2*x*	Fresh
		2*x*	Silica gel-dried
		2*x*	Herbarium

As two species were used as the diploid standard, we tested replicate samples of the *R. maddenii* complex to validate FCM ploidy using both standards. Interestingly, all 10 polyploid accessions showed discrepant ploidy results between the two runs (Table 4 and see Supporting Information—Table S3). Ploidy estimation using *R. parraye* was one or two levels lower than that using *R. fortunei,* and there was a higher incidence of odd-numbered results with the *R. parryae* standard. Further examination demonstrated that the genome size of *R. parryae* (2C = 1.70–1.75 pg) was larger than *R. fortunei* (2C = 1.52–1.57 pg) [see Supporting Information—Table S4]. This explains the lower FCM ploidy level calculated for the same accession, when using the former as the diploid standard.

**Table 4.** Flow cytometry ploidy estimates of samples repeated with two diploid standards. ^1^All results based on fresh leaves. FCM histograms are shown in Supporting Information—Table S3

Ss. *Maddenia* taxon	Acc. ID	FCM ploidy^1^	Diploid standard
*R. maddenii* ssp. *crassum*	PK22	8*x*	*R. fortunei*
		7*x*	*R. parryae*
	PK45	7*x*	*R. fortunei*
		6*x*	*R. parryae*
	PK59	6*x*	*R. fortunei*
		5*x*	*R. parryae*
	PK61	7*x*	*R. fortunei*
		6*x*	*R. parryae*
*R. maddenii* ssp. *maddenii*	PK09	7*x*	*R. fortunei*
		5*x*	*R. parryae*
	PK17	8*x*	*R. fortunei*
		7*x*	*R. parryae*
	PK27	6*x*	*R. fortunei*
		5*x*	*R. parryae*
	PK38	6*x*	*R. fortunei*
		5*x*	*R. parryae*
	PK52	6*x*	*R. fortunei*
		5*x*	*R. parryae*
	PK68	6*x*	*R. fortunei*
		5*x*	*R. parryae*

#### Meiotic chromosome counts in representative samples

**Figure 3.**
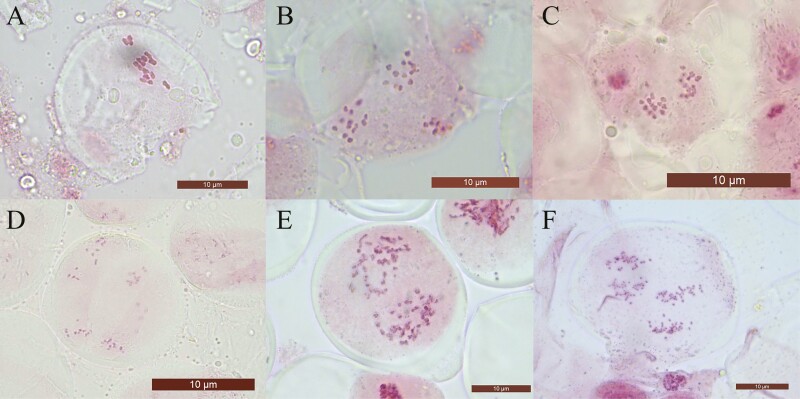
Meiotic chromosomes of ss. *Maddenia* taxa sampled from flower buds, with ploidy levels. (A) OM01, *R. formosum* var. *inaequale*, *n* = 13, 2*x*. (B) OM06, *R. ciliicalyx*, *n* = 13, 2*x*. (C) OM41, *R. carneum*, *n* = 13, 2*x*. (D) OM55, *R. burmanicum*, *n* = 13, 2*x.* (E) OM17, *R. maddenii*, *n* = 52, 8*x*. (F) OM20, *R. maddenii*, *n* = 39, 6*x*. All scale bars 10 µm.

Representative accessions were selected for meiotic chromosome counting to validate the FCM ploidy estimates (Fig. 3). We observed four diploids, one hexaploid (OM20 *R. maddenii* ssp. *maddenii*, Fig. 3F) and one octoploid (OM17 *R. maddenii* ssp. *crassum*, Fig. 3E). The validated diploid accessions were: OM01 *R. formosum* vaR. *inaequale* (Fig. 3A), OM06 *R. ciliicalyx* (Fig. 3B), OM41 *R. carneum* (Fig. 3C) and OM55 *R. burmanicum* (Fig. 3D). The ploidy results demonstrated consistency with those obtained from flow cytometry, as well as those from previously reported chromosome numbers (Table 2).

## Discussion

### High ploidy variation in genus *Rhododendron*, with the most frequent polyploidy in subgenera *Pentanthera* and *Rhododendron*

Our summary dataset (Fig. 2) is the first and most systematic compilation of the ploidy levels of *Rhododendron* species to date. It reveals that 31 % of the 1,376 *Rhododendron* taxa have been examined for ploidy while 69 % are yet to be investigated. Because databases such as CCDB do not currently cover all the *Rhododendron* taxa that have been reported, the datasheet [see Supporting Information—Table S1] can be used to update the relevant databases. Of the taxa for which data have been reported, 78 % (332) (24 % of all *Rhododendron* taxa) are diploids while polyploidy has been found in 22 % (92) (7 % of all) taxa (Ammal *et al.* 1950; Väinölä 2000; Rice *et al.* 2015; Khan *et al.* 2021). The larger subgenera (*Azaleastrum, Hymenanthes*, *Pentanthera*, *Rhododendron*, *Vireya*) all exhibit polyploidy to some degree, while in the smaller subgenera (*Candidastrum*, *Mumeazalea*, *Therorhodion*) and subgenus *Tsutsusi*, all species tested thus far are diploids. The highest frequency of polyploids is in subgenus *Pentanthera* (38 % of the reported in this subgenus) then subgenus *Rhododendron* (36 %) (Table 1), which agrees with the findings in Jones *et al.* (2007). The highest ploidy variation is found in subgenus *Rhododendron*, ranging from triploid (3*x* = 39) to dodecaploid (12*x* = 156) [see Supporting Information—Table S1]. Particularly, taxa reported in subsections *Baileya*, *Cinnabarina*, *Heliolepida*, *Rhodorastra* of subgenus *Rhododendron* are all polyploids (Fig. 2; Table 1). Chromosome counts for most of the taxa support that the basic chromosome number in genus *Rhododendron* is *x* = 13 (2*n* = 26), although 2*n* = 24 has been reported in seven taxa [see Supporting Information—Table S1]. This difference may result from accessions that were rare aneuploids (Jones and Brighton 1972), or likely miscounts due to technological limitations at the time of early cytological studies of *Rhododendron* (Bowers 1930).

Natural ploidy series, with both diploids and polyploids, have been reported in several taxa of *Rhododendron*. Ploidy variation has been reported among species in complexes such as *R. maddenii*, *R. telmateium* and *R. yunnanense*, as well as polyploid series within species *R. flavidum* and *R. occidentale* [see Supporting Information—Table S1]. Jones *et al.* (2007) proposed that the polyploid series within *R. occidentale* was from species diversification while that within *R. flavidum* may result from an unresolved species boundary with *R. calendulaceaum*. Ploidy variation could arise from intraspecific variability (i.e. cytotype diversity) (Husband *et al.* 2013; Farhat *et al.* 2019) and reflects the high diversification rate of flora in the Himalayan region (Schwery *et al.* 2015; Yan *et al.* 2015; Shrestha *et al.* 2018). However, it could also be related to the unresolved taxonomy. That is, in cases where multiple taxa have been ‘lumped’ into one, it is possible that one or some of the previously recognised taxa had a particular ploidy level, which presents as a series within a set of accessions that encompass the synonymous taxa. A third issue might be incorrect field labelling leading to incorrect grouping of samples, although this could be greatly improved with morphological identification by the researcheR. This identification issue was also addressed for taxonomic correction in studies of other plant genera such as *Deutzia* Thunb. (Hydrangeaceae) (Hembree *et al.* 2020). Resolving the taxonomy of polyploid complexes will significantly inform decision making on conservation of biodiversity at several levels (e.g. phenotypic, phylogenetic or species diversity) (Ennos *et al.* 2005; Laport and Ng 2017).

Notably, a discrepancy among ploidy levels was found between chromosome counts and flow cytometry reported in previous studies of *Rhododendron*. Polyploids were identified only from either flow cytometry (e.g. subgenus *Vireya*, subsection *Rhodorastra*) or chromosome counting (e.g. *R. baileyi*) when both methods have been applied [see Supporting Information—Table S1]. This occasional discrepancy is observed more likely from the two largest ploidy studies of *Rhododendron*: Ammal *et al.* (1950) using chromosome counting and Khan *et al.* (2021) using flow cytometry. Limited coverage of samples might be the primary reason for this discrepancy, such that intraspecific ploidy variation was not captured. Another factor that may influence the ploidy discovered is more recent sampling from garden cultivation rather than from the wild, as in cultivation there may be more polyploids due to their favourable horticultural features (Jones *et al.* 2008; Rodionov *et al.* 2019). Given that flow cytometry is less time-consuming than chromosome counting, additional sampling for flow cytometry from wild populations should be considered to further understand the ploidy variation in *Rhododendron* taxa.

### Subsection *Maddenia* consisting of diploids except for the *R. maddenii* complex

Our ploidy estimations using flow cytometry present the most comprehensive ploidy analysis of ss. *Maddenia* reported to date. We have made the first ploidy reports for 12 taxa, increasing the known number of diploid taxa in this subsection to 47 of the total 51 taxa studied (Table 2 and [see Supporting Information—Table S2]. In agreement with previous studies, polyploids are present in ss. *Maddenia*, but only in the two subspecies of *R. maddenii*. The only two polyploid exceptions reported outside the *R. maddenii* complex were one octoploid (2*n* = c.104) *R. taronense* (Cubey 2003) and one tetraploid (4*x*) *R. carneum* (Khan *et al.* 2021), but both were from one accession only. Our FCM results from multiple accessions together with other previous chromosome counts identified only diploids in both species, supporting the conclusion that these two species are diploids (Table 2 and see Supporting Information—Table S1).

The *R. maddenii* complex reveals a ploidy series consisting of diploids and polyploids varying from pentaploid to octoploid (5–8*x*) (Table 2). This is consistent with the previous reports from flow cytometry (Jones *et al.* 2007; De *et al.* 2010; Khan *et al.* 2021), where hexaploids (6*x*) and octoploids (8*x*) were reported. Tetraploids (2*n* = 52) were identified in previous chromosome counts but not in the present study (Ammal *et al.* 1950; Darlington *et al.* 1955). However, it is not known whether the polyploid complex within *R. maddenii* results from intraspecific variability or a combination of ploidy levels due to the ‘lumped’ taxa. Also, we lack data as to whether the *R. maddenii* polyploids are allopolyploids or autopolyploids. Cubey (2003) proposed an autopolyploid origin of the polyploids in *R. maddenii*, when there were no distinguishable morphological characters among plants of different ploidy levels, and diploids were rarely found in this species. Although *R. maddenii* was assessed as a species of Least Concern in the Red List (Gibbs *et al.* 2011), we advocate studies of the two putative subspecies to determine whether the synonymous taxa require species recognition, which might result in a revision of their conservation status.

In the whole genus, natural anisoploidy is not common, although triploids (3*x* or 2*n* = 39) have been reported in nine species and a pentaploid (5*x*) in one species [see Supporting Information—Table S1]. Our study of ss. *Maddenia* revealed some anisoploids (5*x*, 7*x*) in the *R. maddenii* complex (Table 2). More *Rhododendron* anisoploids, usually triploids, are bred for horticulture, but little is known about their reproductive biology (Li 1957; Jones and Ranney 2009). The pentaploid (5*x*) and heptaploid (7*x*) plants in our study do produce flowers, but their fertility and the mechanism as to how these anisoploids arose await further investigation.

In general, *R. maddenii* ssp. *crassum* tends to present a higher ploidy level with more octoploids (8*x*) than *R. maddenii* ssp. *maddenii* which includes more hexaploids (6*x*) (Table 2 and see Supporting Information—Table S1). This may be an infraspecific distinction and related to the geographic distribution of the two subspecies. Cullen (1980) compared the geographic distribution of the two subspecies of *R. maddenii.* While he suggested that the morphological variation in *R. maddenii* was unrelated to geography, our results show a possible trend in the ploidy levels between the two subspecies. However, the relationship between ploidy and geographic pattern in this species complex is still under investigation and requires further carefully structured field surveys.

The *R. maddenii* complex shows a similar pattern of ploidy variation and geographic distribution as the genus *Buddleja* L. (Scrophulariaceae) which is also from the Sino-Himalayan region (Cullen 1980; Chen *et al.* 2007). One hypothesis for the ploidy differences between the two subspecies of *R. maddenii* may be the ongoing *in situ* speciation in this area (Hughes 2017). As one of the world’s youngest mountain ranges, with a high frequency of polyploidy in plants, the Sino-Himalayan region has been identified as a centre of species diversification that may be attributed to polyploidization (Irving and Hebda 1993; Schwery *et al.* 2015; Xing and Ree 2017; Shrestha *et al.* 2018; Rice *et al.* 2019; Xia *et al.* 2021). However, the Sino-Himalayan origin does not explain why polyploids should have continued to occur in *R. maddenii*, while other species in ss. *Maddenia* which are predominantly from the same region do not exhibit polyploids. Further research on a wider range of wild samples, from across the geographic range, and particularly from the *R. maddenii* complex, would inform this question.

### Flow cytometry as a useful tool for estimating ploidy level of *Rhododendron,* even with dehydrated leaves

Despite few reports in previous studies (De Schepper *et al.* 2001; Jones *et al.* 2007; Khan *et al.* 2021), the consistency of ploidy identified by flow cytometry and meiotic chromosome counts in our study supports the reliability of flow cytometry for ploidy estimation of *Rhododendron* (Fig. 3). Our trial with replicated samples verified higher reliability of silica gel-dried over herbarium leaves of *Rhododendron* for FCM ploidy assessment (Table 3). Although not preferred, dehydrated, particularly silica gel-dried leaf tissue, has been successful in other plant groups for ploidy estimation with the standard DAPI protocol using flow cytometry, thereby eliminating the inconvenience of collecting and preserving fresh samples (Šmarda 2006; Šmarda and Stančík 2006; Suda and Trávníček 2006; Farhat *et al.* 2019; Tomaszewska *et al.* 2021). The efficiency of flow cytometry with dehydrated leaves from herbarium specimens might be limited by several factors, including insufficient amount of tissue, sampling of mature leaves rather than newly expanding ones, incorrect drying, storage and preservation of samples and the limited efficacy of nuclei isolation due to degradation (Tomaszewska *et al.* 2021).

Although flow cytometry can be convenient for ploidy determination of a large number of samples, especially for identifying the frequency of polyploids (Jones *et al.* 2007; Kron *et al.* 2007; Hembree *et al.* 2020; Tomaszewska *et al.* 2021), this technique may not always yield definitive results (Suda *et al.* 2006). In our results for all samples that were interpreted as polyploids, histogram peaks of the higher-ploidy samples tended to be significantly lower than that of the diploid standard. Such small peaks may be missed, or the decision between diploid or polyploid may be difficult to interpret in data analysis [see Supporting Information—Table S3]. In addition, in some cases the ratio of two peaks on the histogram was near the midpoint between euploids, and the interpreted ploidy was therefore approximate (e.g., PK17 *R. maddenii* ssp. *maddenii*: 7–8*x*; Table 4 and [see Supporting Information—Table S3]). In such cases, we repeated samples without co-chopped standard tissue, to verify the results. In addition, our use of two diploid standards (*R. fortunei* and *R. parryae*) due to constraints on availability of fresh material in some gardens, brought further challenge to interpreting the ploidy of some accessions. Differing values of DNA contents from different standards slightly change the calculated ploidy ratio, particularly for polyploids. Our results for *R. fortunei* and *R. parryae* fall within the range of genome sizes of reported *Rhododendron* diploids (Bou Dagher-Kharrat *et al.* 2013; Khan *et al.* 2021; Choi *et al.* 2022). However, the larger genome size of *R. parryae* likely caused the interpreted ploidy for the same accessions being one level lower than using *R. fortunei* as the standard (Table 4 and [see Supporting Information—Table S4]). Consistent use of a single standard in a study is therefore recommended, and we anticipate further use of these two *Rhododendron* species as diploid standards in future research for the genus. Nevertheless, due to the degree of unavoidable uncertainty of ploidy determination by flow cytometry, we suggest that chromosome counting is still the ‘gold standard’ approach to determining ploidy level.

In previous studies, most *Rhododendron* chromosome counts were made from root tips grown from seed (Jones *et al.* 2007; De *et al.* 2010; Zaytseva *et al.* 2018). However, this may introduce errors in the ploidy determination, as seedlings from species in cultivation are likely to be from open-pollinated seeds and hence may be hybrids. For this reason, we used developing stamens in flower buds as the source of tissue for chromosome counts, which requires careful observation of the timing of flower bud development. *Rhododendron* flower buds move into dormancy shortly after flower differentiation. The onset and duration of rest depend upon the presence of the flower bud scales (Schneider 1968). Mirgorodskaya *et al.* (2015) reported that the microspores of the evergreen species *R. catawbiense* in Russia underwent meiosis at the end of the summer (i.e. in August) and overwintered at the vacuolization stage. Mitosis with the formation of bicellular pollen grains occurred shortly before flowering at the beginning of summer in the following year (i.e. in June). In light of these observations, we suggest harvesting flower buds with dividing microspore mother cells after blooming and close to winter dormancy, at the late stage of flower differentiation. However, it may take the entire season to observe development and identify the correct stage for sampling. Meiotic chromosomes can only be observed once the buds are dissected under the microscope after fixation and staining, which increases the difficulty of harvesting inflorescence buds at the desired development stage and requires constant sampling in the field. *Rhododendron* chromosomes are small and difficult to view under the microscope (Jones *et al.* 2007), which makes it more difficult to assess polyploids with multiple sets of chromosomes (Windham *et al.* 2020). In some cases, even a physical chromosome count cannot confirm the number of chromosomes, due to overlapping chromosomes, or abnormal chromosome behaviours (e.g. lagging chromosomes) in polyploids (Li 1957; Contreras *et al.* 2007). Other possible approaches to ploidy estimation such as targeted capture sequencing may allow discovery of polyploid characteristics (Viruel *et al.* 2019; Tahir *et al.* 2020).

## Conclusion

Phylogenetic analysis, commonly used to provide knowledge on species relationships and in turn for conservation planning, can be confounded by the presence of polyploids in a set of samples. In the ‘big genus’ (Frodin 2004) *Rhododendron* that presents both complex taxonomy and considerable conservation problems, prior studies reported polyploids in several species. We investigated the taxonomic distribution of polyploidy in the whole genus, and particularly ploidy levels of taxa in ss. *Maddenia.* Polyploidy occurs across the genus with 22 % polyploids among the reported 424 species, with the highest frequency in subgenera *Pentanthera* and *Rhododendron*. However, the genus remains largely underexamined for ploidy, with no report for 69 % of *Rhododendron* taxa.

Flow cytometry is a suitable tool for ploidy estimation in *Rhododendron*. When fresh tissue is unavailable, silica gel-dried leaves are more reliable than leaves from herbarium samples. We used flow cytometry to estimate ploidy for 47 taxa in ss. *Maddenia*, including 12 taxa that had never been investigated in previous studies. In this subsection, polyploids have been definitively identified in only the *R. maddenii* complex, where its two subspecies exhibit ploidy series consisting of diploids and various polyploidy levels. The ploidy variation in the *R. maddenii* complex may be a factor of the unresolved taxonomy or of the diversification of the species across a broad geographic range. Broader sampling from wild populations should be considered in future research to resolve the relationship between taxon geography and ploidy levels.

Current botanic garden accessions of ss. *Maddenia*, especially those from the wild, can be analysed with next-generation sequences mapped against published *Rhododendron* genomes (Zhang *et al.* 2017; Soza *et al.* 2019; Yang *et al.* 2020; Ma *et al.* 2021; Zhou *et al.* 2022) to understand character evolution, especially for those characters used to resolve species taxonomy. More immediately, the present ploidy estimations from our samples will prompt our phylogenetic study of ss*. Maddenia*. Eventually, knowledge of resolved taxonomic debates will underpin the priorities in ss. *Maddenia* for conservation actions.

## Supporting Information

The following additional information is available in the online version of this article—

Table S1. Reported ploidy of taxa in *Rhododendron* L. (Ericaceae).

Table S2. Ploidy estimation of taxa in subsection *Maddenia* using flow cytometry in the present study.

Table S3. Flow cytometry histograms of subsection *Maddenia* accessions with inconsistent ploidy in different runs.

Table S4. Genome size measurements of *R. fortunei* Lindl. and *R. parryae* Hutch.

plad016_suppl_Supplementary_Table_S1

plad016_suppl_Supplementary_Table_S2

plad016_suppl_Supplementary_Table_S3

plad016_suppl_Supplementary_Table_S4

## Data Availability

All data can be found within the paper and its supporting materials, otherwise available on request from the corresponding author.

## Notes

**Natural History and Conservation. Chief Editor**: F. Xavier

## Declarations

Plant specimens from Royal Botanic Garden Edinburgh were collected under Convention on Biological Diversity (permit ID: 266). Plant material from Royal Botanic Garden Edinburgh and Rhododendron Species Botanical Garden were imported to Massey University (Palmerston North, NZ) using Ministry for Primary Industries permit 2021078031.
