# Supplementary material for: Ploidy variation in Rhododendron subsection Maddenia and its implications for conservation
Source: AoB Plants. 2023 Apr 12;15(3):plad016. doi: 10.1093/aobpla/plad016 (PMC10184449; doi:10.1093/aobpla/plad016)
Supplement: plad016_suppl_Supplementary_Table_S1 [file plad016_suppl_supplementary_table_s1.docx]

**Table S1.** Reported ploidy of taxa in *Rhododendron* L. (Ericaceae), 2*n* = 2*x* = 26. **1)** Taxonomic classification (subgenus - section - subsection) followed Chamberlain et al. (1996) and Argent (2015) [1, 2]. For resolved names, non-vireya taxa were indexed according to Chamberlain et al. [1] while vireyas (subgenus Vireya, also known as section *Schistanthe*) followed Argent [2]. **2)** Data of horticultural cultivars that were studied previously are presented here (noted as “cultivation source” in column “Resolved name”), but not included in our calculation of taxon numbers (Table 1 and Fig. 2), as our study focuses on "taxa" which include species, subspecies and botanical varieties. **3)** Despite unresolved taxonomy, *R. vanderbiltianum* is included because of its suggested placement in subsection *Maddenia* [3-5]. **4)** Data in Cubey (2003) [6] are partially presented due to inaccessibility of the PhD thesis, with chromosome counts of subsection *Maddenia* included, but not subsection *Saluenensia*.

| **Subgenus** | **Section** | **Subsection** | **Taxon in original literature** | **Resolved name** | **FCM ploidy (*x*)** | **Chromosome count (2*n*)** | **Reference** |
| --- | --- | --- | --- | --- | --- | --- | --- |
| unknown | unknown | unknown | *R. cunninghami* T. More | *R.* 'Cunninghamii'? (cultivation source) |  | 26 | [7] |
| unknown | unknown | unknown | *R. cunninghami* T. More (*R. cinnamomeum* C. Paxt.) | *R.* 'Cunninghamii'? (cultivation source) |  | 26 | [8] |
| unknown | unknown | unknown | *R. omurasaki* | *R.* o-murasaki ‘Makino’ (cultivation source) |  | 26 | [9] |
| unknown | unknown | unknown | *R. lateritium* | *R.* 'Lateritium' (cultivation source) |  | 26 | [10] |
| unknown | unknown | unknown | *R. praecox* | (cultivation source) |  | 26 | [10] |
| unknown | unknown | unknown | *R. viscosepalum* | (cultivation source) |  | 26 | [10] |
| *Azaleastrum* (1/11/38; 2 sections) | *Azaleastrum* (1/3/11) | \ | *R. leptothrium* Balf. f. & Forrest | *R. leptothrium* Balf. & Forrest |  | 26 | [11, 12] |
|  |  |  | *R. ovatum* (Lindley) Maximowicz | *R. ovatum* (Lindley) Maximowicz | 4*x* |  | [13] |
|  |  |  | *R. vialii* Delavay & Franch. | *R. vialii* Delavay & Franch. |  | 26 | [11] |
|  | *Choniastrum* (0/8/27) | \ | *R. championae* Hooker | *R. championae* Hooker | 2*x* |  | [13] |
|  |  |  | *R. hancockii* Hemsl. | *R. hancockii* Hemsl. |  | 26 | [11] |
|  |  |  | *R. amamiense* Ohwi | *R. latoucheae* Franch. |  | 26 | [11] |
|  |  |  | *R. mackenzianum* Forrest | *R. moulmainense* Hook.f. |  | 26 | [11] |
|  |  |  | *R. stamineum* Franch. | *R. stamineum* Franch. |  | 26 | [11] |
|  |  |  | *R. truncatovarium* L.M. Gao & D.Z. Li | *R. truncatovarium* L. M. Gao & D. Z. Li |  | 26 | [11] |
|  |  |  | *R. tutcherae* Hemsl. & E.H. Wilson | *R. tutcherae* Hemsl. & E. H. Wilson |  | 26 | [11] |
|  |  |  | *R. tutcherae* var. *glabrifolium* L.M. Gao & D.Z. Li | *R. tutcherae* var. *glabrifolium* L.M. Gao & D.Z. Li |  | 26 | [11] |
| *Candidastrum* (0/1/2) | \ | \ | *R. albiflorum* | *R. albiflorum* Hook. |  | 26 | [12] |
| *Hymenanthes* (7/149/(436 - 9 unplaced) = 7/149/427; 1 section - 24 subsections) | *Ponticum* [7/(148 + 1 diploid taxon unplaced for subsection)/427 = 7/149/427] | (unplaced) | *R. batemanii* Hook. | *R*. × *batemannii* Hook.f. (1863) (cultivation source) |  | 26 | [12] |
|  |  | (unplaced) | *R. beimaense* | *R.* × *erythrocalyx* Balf.f. & Forrest (cultivation source) |  | 26 | [12] |
|  |  | (unplaced) | *R. planetum* | *R. planetum* Balf.f. |  | 26 | [12] |
|  |  | *Arborea* (1/4/17) | *R. arboreum* (ssp. *arboreum*) Smith | *R. arboreum* (ssp. *arboreum*) Smith | 2*x* | 26 | [7, 8, 12, 13] |
|  |  |  | *R. zeylanicum* | *R. arboreum* Sm. ssp. *zeylanicum* (Booth) Tagg |  | 26 | [12] |
|  |  |  | *R. silvaticum* | *R. lanigerum* Tagg |  | 26 | [12] |
|  |  |  | *R. niveum* Hook. f. | *R. niveum* Hook. f. | 6*x* | 26 | [12, 14] |
|  |  | *Argyrophylla* (0/7/31) | *R. adenopodum* | *R. adenopodum* Franch. |  | 26 | [12] |
|  |  |  | *R. argyrophyllum* | *R. argyrophyllum* Franch. |  | 26 | [12] |
|  |  |  | *R. argyrophyllum* ssp. *nankingense* (Cowan) Chamberlain | *R. argyrophyllum* ssp. *nankingense* (Cowan) Chamberlain | 2*x* |  | [13] |
|  |  |  | *R. hunnewellianum* | *R. hunnewellianum Rehder & E.H.Wilson* |  | 26 | [12] |
|  |  |  | *R. insigne* | *R. insigne* Hemsl. & E.H.Wilson |  | 26 | [12] |
|  |  |  | *R. ririei* Hemsley & Wilson | *R. ririei* Hemsley & Wilson | 2*x* | 26 | [12, 13] |
|  |  |  | *R. thayerianum* Rehder & Wilson | *R. thayerianum* Rehder & Wilson | 2*x* | 26 | [12, 13] |
|  |  | *Auriculat*a (1/1/2) | *R. auriculatum* Hemsley | *R. auriculatum* Hemsley | 2*x*, 4*x* | 26 | [12, 13] |
|  |  | *Barbata* (0/2/5) | *R. smithii* Nutt. | *R. argipeplum* Balf.f. & R.E.Cooper |  | 26 | [12] |
|  |  |  | *R. barbatum* Wall. ex G. Don | *R. barbatum* Wall. ex G. Don |  | 26 | [8, 12] |
|  |  | *Campanulata* (0/2/4) | *R. campanulatum* Don | *R. campanulatum* D. Don |  | 26 | [8, 12] |
|  |  |  | *R. wallichii* /*R. campanulatum* var. *wallichii* Hook. | *R. wallichii* Hook.f. |  | 26 | [8, 12] |
|  |  | *Campylocarpa* (0/6/10) | *R. callimorphum*/*R. cyclium* | *R. callimorphum* Balf.f. & W.W.Sm. (var. *callimorphum*) |  | 26 | [12] |
|  |  |  | *R. campylocarpum* Hook. f. | *R. campylocarpum* Hook. |  | 26 | [12] |
|  |  |  | *R. campylocarpum* ssp. *caloxanthum* /*R. caloxanthum*/*R. telopeum* | *R. campylocarpum* ssp. *caloxanthum* (Balfour & Farrer) Chamberlain | 2*x* | 26 | [12, 13] |
|  |  |  | *R. souliei* | *R. souliei* Franch. |  | 26 | [12] |
|  |  |  | *R. wardii*/*R. croceum*/*R. litiense* | *R. wardii* W.W.Sm. (var*. wardii*) |  | 26 | [12] |
|  |  |  | *R. wardii* var. *puralbum* (Balfour & Smith) Chamberlain | *R. wardii* var. *puralbum* (Balfour & Smith) Chamberlain | 2*x* |  | [13] |
|  |  | *Falconera* (1/9/17) | *R. decipiens* Lacaita | *R.* × *decipiens* Lacaita (cultivation source) | 2*x* |  | [14] |
|  |  |  | *R. arizelum* | *R. arizelum* Balf. & Forrest |  | 26 | [12] |
|  |  |  | *R. basilicum* | *R. basilicum* Balf.f. & W.W.Sm. |  | 26 | [12] |
|  |  |  | *R. coriaceum* | *R. coriaceum* Franch. |  | 26 | [12] |
|  |  |  | *R. falconeri* Hook. f | *R. falconeri* Hook. f | 2*x* | 26 | [12, 14] |
|  |  |  | *R. eximium* Nutt. | *R. falconeri* Hook.f. ssp. *eximium* (Nutt.) D.F.Chamb. |  | 26 | [12] |
|  |  |  | *R. galactinum* Tagg | *R. galactinum* Tagg | 2*x* | 26 | [12, 13] |
|  |  |  | *R. hodgsonii* Hook. f. | *R. hodgsonii* Hook. |  | 26 | [7, 8, 12] |
|  |  |  | *R. rex* | *R. rex* H. Lév. |  | 52 | [15] |
|  |  |  | *R. fictolacteum* | *R. rex* H. Lév. ssp. *fictolacteum* (Balf.f.) D.F.Chamb. |  | 26 | [12] |
|  |  | *Fortunea* (1/11/51) | *R. calophytum* | *R. calophytum* Franch. |  | 26 | [12] |
|  |  |  | *R. decorum* | *R. decorum* Franch. |  | 26 | [12] |
|  |  |  | *R. diaprepes*/*R. decorum* ssp. *diaprepes* (Balfour & Smith) Ming | *R. decorum* ssp. *diaprepes* (Balfour & Smith) Ming | 2*x* | 26, 39 | [13, 16] |
|  |  |  | *R. discolor* | *R. fortunei* Lindl. ssp. *discolor* (Franch.) D.F.Chamb. |  | 26 | [12] |
|  |  |  | *R. fortunei* | *R. fortunei* Lindl. | 2*x* |  | [17] |
|  |  |  | *R. griffithianum* Cooper | *R. griffithianum* Wight |  | 26 | [12] |
|  |  |  | *R. orbiculare* | *R. orbiculare* Decne. |  | 26 | [12] |
|  |  |  | *R. oreodoxa* | *R. oreodoxa* Franch. |  | 26 | [12] |
|  |  |  | *R. fargesii*/*R. erubescens* | *R. oreodoxa* Franch. var. *fargesii* (Franch.) D.F.Chamb. |  | 26 | [12] |
|  |  |  | *R. praevernum* Hutchinson | *R. praevernum* Hutchinson | 2*x* | 26 | [12, 13] |
|  |  |  | *R. sutchuenense* | *R. sutchuenense* Franch. |  | 26 | [12] |
|  |  | *Fulgensia* (0/1/3) | *R. fulgens* Hook. f. | *R. fulgens* Hook. f. | 2*x* | 26 | [12, 14] |
|  |  | *Fulva* (0/3/4) | *R. fulvoides* | *R. fulvoides* Balf.f. & Forrest |  | 26 | [12] |
|  |  |  | *R. fulvum* | *R. fulvum* Balf.f. & W.W.Sm. |  | 26 | [12] |
|  |  |  | *R. niphargum* | *R. uvarifolium* Diels var. *uvarifolium* |  | 26 | [12] |
|  |  | *Glischra* (0/5/11) | *R. crinigerum* | *R. crinigerum* Franch. |  | 26 | [12] |
|  |  |  | *R. diphrocalyx* | *R. diphrocalyx* Balf.f. |  | 26 | [12] |
|  |  |  | *R. glischrum* | *R. glischrum* Balf.f. & W.W.Sm. |  | 26 | [12] |
|  |  |  | *R. habrotrichum* | *R. habrotrichum* Balf.f. & W.W.Sm. |  | 26 | [12] |
|  |  |  | *R. recurvoides* Tagg & Kingdon-Ward | *R. recurvoides* Tagg & Kingdon-Ward | 2*x* |  | [13] |
|  |  | *Grandia* (0/9/17) | *R. grande Wight* | *R. grande* Wight | 2*x* | 26 | [7, 8, 14, 18] |
|  |  |  | *R. macabeanum* Watt. | *R. macabeanum* Watt ex Balf.f. | 2*x* | 26 | [12, 13] |
|  |  |  | *R. montroseanum* Davidian | *R. montroseanum* Davidian | 2*x* |  | [13] |
|  |  |  | *R. coryphaeum* | *R. praestans* Balf.f. & W.W.Sm. |  | 26 | [12] |
|  |  |  | *R. protistum* Balfour & Forrest | *R. protistum* Balfour & Forrest | 2*x* | 26 | [12, 13] |
|  |  |  | *R. giganteum* | *R. protistum* Balfour & Forrest var. *gigantum* (Forrest ex Tagg) D.F.Chamb. |  | 26 | [12] |
|  |  |  | *R. sidereum* | *R. sidereum* Balf.f. |  | [12] | [12] |
|  |  |  | *R. sinogrande* | *R. sinogrande* Balf.f. & W.W.Sm. | 2*x* | 26 | [12, 17] |
|  |  |  | *R. watsonii* | *R. watsonii* Hemsl. & E.H.Wilson |  | 26 | [12] |
|  |  | *Griersoniana* (0/1/1) | *R. griersonianum* Balfour & Forrest | *R. griersonianum* Balfour & Forrest | 2*x* |  | [12, 13] |
|  |  | *Irrorata* (0/7/33) | *R. aberconwayi* | *R. aberconwayi* Cowan |  | 26 | [12] |
|  |  |  | *R. annae* Franchet/*R. hardingii* | *R. annae* Franchet | 2*x* | 26 | [12, 13] |
|  |  |  | *R. araiophyllum* | *R. araiophyllum* Balf. & W. W. Sm. |  | 26 | [12] |
|  |  |  | *R. hylothreptum* | *R. anthosphaerum* Diels |  | 26 | [12] |
|  |  |  | *R. irroratum*/*R. ningyuenense* (correction: *ninguenense*) | *R. irroratum* Franch. (ssp. *irroratum*) |  | 26 | [12] |
|  |  |  | *R. lukiangense*/*R. cerasinum* | *R. lukiangense* Franch. |  | 26 | [12] |
|  |  |  | *R. shepherdii* Nutt. | *R. kendrickii* Nutt. |  | 26 | [12] |
|  |  | *Lanata* (0/1/9) | *R. lanatum* Hook. f | *R. lanatum* Hook. |  | 26 | [12] |
|  |  | *Maculifera* (0/5/24) | *R. longesquamatum* | *R. longesquamatum* C.K.Schneid |  | 26 | [12] |
|  |  |  | *R. maculiferum* | *R. maculiferum* Franch. |  | 26 | [12] |
|  |  |  | *R. morii* | *R. morii* Hayata |  | 26 | [12] |
|  |  |  | *R. pachytrichum* | *R. pachytrichum* Franch. |  | 26 | [12] |
|  |  |  | *R. strigillosum* Franchet | *R. strigillosum* Franchet | 2*x* | 26 | [12, 13] |
|  |  | *Neriiflora* (1/19/50) | *R. aperantum* | *R. aperantum* Balf. & Kingdon-Ward |  | 26 | [12] |
|  |  |  | *R. beanianum* | *R. beanianum* Cowan |  | 26 | [12] |
|  |  |  | *R. citriniflorum* (var*. citriniflorum*) | *R. citriniflorum* Balf.f. & Forrest (var. *citriniflorum*) | 4*x* | 26 | [12, 13] |
|  |  |  | *R. dichroanthum* | *R. dichroanthum* Diels |  | 26 | [12] |
|  |  |  | *R. apodectum* | *R. dichroanthum* Diels ssp. *apodectum* (Balf. & W. W. Sm.) Cowan |  | 26 | [12] |
|  |  |  | *R. scyphocalyx* | *R. dichroanthum* Diels ssp. *scyphocalyx* (Balf.f. & Forrest) Cowan |  | 26 | [12] |
|  |  |  | *R. floccigerum* | *R. floccigerum* Franch. |  | 26 | [12] |
|  |  |  | *R. repens* | *R. forrestii* Balf.f. ex Diels ssp. *forrestii* |  | 26 | [12] |
|  |  |  | *R. haematodes* | *R. haematodes* Franch. |  | 26 | [12] |
|  |  |  | *R. chaetomallum* Balf. f & Forrest | *R. haematodes* subsp. *chaetomallum* (Balf. & Forrest) D. F. Chamb. |  | 26 | [12] |
|  |  |  | *R. mallotum* | *R. mallotum* Balf.f. & Kingdon-Ward |  | 26 | [12] |
|  |  |  | *R. neriiflorum* Franchet | *R. neriiflorum* Franchet | 2*x* | 26 | [12, 13] |
|  |  |  | *R. euchaites* | *R. neriiflorum* Franch. ssp. *neriiflorum* |  | 26 | [12] |
|  |  |  | *R. phaedropum* | *R. neriiflorum* Franch. ssp. *phardropum* (Balf.f. & Barrer) Tagg |  | 26 | [12] |
|  |  |  | *R. pocophorum* | *R. pocophorum* Balf.f. ex Tagg |  | 26 | [12] |
|  |  |  | *R. sanguineum* | *R. sanguineum* Franch. |  | 26 | [12] |
|  |  |  | *R. didymum* | *R. sanguineum* Franch. ssp. *didymum* (Balf.f. & Forrest) Cowan |  | 26 | [12] |
|  |  |  | *R. haemaleum* | *R. sanguineum* Franch. ssp. *sanguineum* var. *haemaleum* (Balf.f. & Forrest) D.F.Chamb. |  | 26 | [12] |
|  |  |  | *R. sperabile* | *R. sperabile* Balf.f. & Farrer |  | 26 | [12] |
|  |  | *Parishia* (0/3/8) | *R. elliottii Watt* | *R. elliottii* Watt ex Brandis | 2*x* | 26 | [12, 13] |
|  |  |  | *R. facetum*/*R. eriogynum* | *R. facetum* Balf.f. & Kingdon-ward |  | 26 | [12] |
|  |  |  | *R. kyawi* (*R. prophantum*) | *R. kyawi* Lace & W.W.Sm. |  | 26 | [12] |
|  |  | *Pontica* (1/18/20) | *R.* × *sochadzeae* Kharadze & Davlian. | *R. caucasicum* × *ponticum* (cultivation source) |  | 26 | [19] |
|  |  |  | *R. ungernii* | *R. ungernii* Trautv. |  | 26 | [12] |
|  |  |  | *R. hyperythrum* | *R. hyperythrum* Hayata |  | 26 | [12] |
|  |  |  | *R. makinoi* | *R. makinoi* Tagg |  | 26 | [12] |
|  |  |  | *R. metternichii* | *R. degronianum* Carrière ssp. *heptamerum* (Maxim.) H.Hara (Maxim.) Sealy |  | 26 | [12] |
|  |  |  | *R. yakusimanum* | *R. degronianum* Carrière ssp. *yakushimanum* (Nakai) Kitam. var. *yakushimanum* (Nakai) Kitam. |  | 26 | [12] |
|  |  |  | *R. aureum* Georgi | *R. aureum* Georgi |  | 26, 52 | [12, 20-27] |
|  |  |  | *R. brachycarpum* | *R. brachycarpum* D.Don ex G.Don | 2*x* | 26 | [12, 13, 28] |
|  |  |  | *R. fauriei* | *R. brachycarpum* D.Don ex G.Don ssp. *fauriei* (Franch.) D.F.Chamb. |  | 26 | [12] |
|  |  |  | *R. catawbiense* | *R. catawbiense* Michx. |  | 24, 26 | [10, 12, 29, 30] |
|  |  |  | *R. caucasicum* Pall. | *R. caucasicum* Pall. |  | 26 | [12, 31] |
|  |  |  | *R. degronianum* | *R. degronianum* Carrière |  | 26 | [12] |
|  |  |  | *R. degronianum* ssp. *yakushimanum*  (Nakai) Hara | *R. degronianum* ssp. *yakushimanum* (Nakai) Hara | 2*x* |  | [13] |
|  |  |  | *R. obtusum 'Kaempferi'*/*R. kaempferi* | *R. kaempferi* Planch. |  | 26 | [10, 12, 30] |
|  |  |  | *R. obtusum japonicum* | *R. kiusianum* Makino var. *kiusianum* |  | 26 | [10, 30] |
|  |  |  | *R. macrophyllum/R. californicum* | *R. macrophyllum* D. Don ex G. Don | 2*x* | 26 | [12, 13, 32] |
|  |  |  | *R. maximum/R. purpureum* | *R. maximum* L. | 2*x* | 24, 26 | [10, 12, 17, 29, 30] |
|  |  |  | *R. ponticum/R. obtusum/R. speciosum* | *R. ponticum* L. | 2*x* | 26 | [9, 10, 12, 13, 17, 33] |
|  |  |  | *R. smirnowii* Trautvetter | *R. smirnowii* Trautvetter | 2*x* | 26 | [10, 13] |
|  |  | *Selensia* (0/5/12) | *R. bainbridgeanum* | *R. bainbridgeanum* Tagg & Forrest |  | 26 | [12] |
|  |  |  | *R. martinianum* | *R. martinianum* Balf.f. & Forrest |  | 26 | [12] |
|  |  |  | *R. selense* (ssp. *probum*) | *R. selense* Franch. (ssp. *selense*) |  | 26 | [12] |
|  |  |  | *R. rhaibocarpum* | *R. selense* Franch. ssp. *dasycladum* (Balf.f. & W.W.Sm.) D.F.Chamb. |  | 26 | [12] |
|  |  |  | *R. selense* ssp. *jucundum* (Balfour & Smith) Chamberlain | *R. selense* Franch. ssp. *jucundum* (Balfour & Smith) Chamberlain | 2*x* |  | [13] |
|  |  | *Taliensia* (0/19/75) | *R. adenophorum* | *R. adenogynum* Diels |  | 26 | [12] |
|  |  |  | *R. aganniphum* | *R. aganniphum* Balf.f. & Kingdon-Ward |  | 26 | [12] |
|  |  |  | *R. flavorufum* | *R. aganniphum* Balf.f. & Kingdon-Ward var. *flavorufum* (Balf.f. & Forrest) D.F.Chamb. |  | 26 | [12] |
|  |  |  | *R. alutaceum* var. *iodes* (Balfour & Forrest) Chamberlain | *R. alutaceum* var. *iodes* (Balfour & Forrest) Chamberlain | 2*x* |  | [13] |
|  |  |  | *R. tritifolium* | *R. alutaceum* var. *russotinctum* (Balfour & Forrest) Chamberlain |  | 26 | [12] |
|  |  |  | *R. balfourianum* | *R. balfourianum* Diels |  | 26 | [12] |
|  |  |  | *R. bathyphyllum* | *R. bathyphyllum* Balf.f & Forrest |  | 26 | [12] |
|  |  |  | *R. beesianum* | *R. beesianum* Diels |  | 26 | [12] |
|  |  |  | *R. bureavii* | *R. bureavii* Franch. |  | 26 | [12] |
|  |  |  | *R. dumicola* | *R. dumicola* Tagg & Forrest |  | 26 | [12] |
|  |  |  | *R. lacteum* | *R. lacteum* Franch. |  | 26 | [12] |
|  |  |  | *R. mimetes* var. *simulans* | *R. mimetes* Tagg & Forrest var. *simulans* Tagg & Forrest |  | 26 | [12] |
|  |  |  | *R. prattii* | *R. prattii* Franch. |  | 26 | [12] |
|  |  |  | *R. roxieanum* | *R. roxieanum* Forrest |  | 26 | [12] |
|  |  |  | *R. traillianum* | *R. traillianum* Forrest & W.W.Sm. |  | 26 | [12] |
|  |  |  | *R. vellereum* | *R. principis* Bureau & Franch. |  | 26 | [12] |
|  |  |  | *R. wasonii* Hemsley & Wilson | *R. wasonii* Hemsley & Wilson | 2*x* | 26 | [12, 13] |
|  |  |  | *R. wightii* Hook. f. | *R. wightii* Hook. |  | 26 | [12] |
|  |  |  | *R. wiltonii* | *R. wiltonii* Hemsl. & E.H.Wilson |  | 26 | [12] |
|  |  | *Thomsonia* (0/9/20) | *R. cyanocarpum* | *R. cyanocarpum* (Franch.) W.W.Sm. |  | 26 | [12] |
|  |  |  | *R. eclecteum* | *R. eclecteum* Balf.f. & Forrest |  | 26 | [12] |
|  |  |  | *R. sikkimense* Pradhan & Lachungpa | *R.* × *sikkimense* Pradhan & Lachungpa (1990) (cultivation source) | 2*x* |  | [14] |
|  |  |  | *R. hookeri* Nutt. aff. | *R. hookeri* Nutt. aff. |  | 26 | [12] |
|  |  |  | *R. hylaeum* Balfour & Farrer | *R. hylaeum* Balfour & Farrer | 2*x* |  | [13] |
|  |  |  | *R. meddianum* | *R. meddianum* Forrest |  | 26 | [12] |
|  |  |  | *R. sherriffii* Cowan | *R. sherriffii* Cowan | 2*x* | 26 | [12, 13] |
|  |  |  | *R. stewartianum* | *R. stewartianum* Diels |  | 26 | [12] |
|  |  |  | *R. thomsonii* | *R. thomsonii* Hook.f. |  | 26 | [12] |
|  |  |  | *R. thomsonii* ssp. *lopsangianum* Hooker | *R. thomsonii* ssp. *lopsangianum* Hooker | 2*x* |  | [13] |
|  |  | *Venatora* (0/1/1) | *R. venator* Tagg | *R. venator* Tagg | 2*x* | 26 | [12, 13] |
|  |  | *Williamsiana* (1/1/2) | *R. williamsianum* Rehder & Wilson | *R. williamsianum* Rehder & Wilson | 4*x* | 26 | [12, 13] |
| *Mumeazalea* (0/1/1) | \ | \ | *R. semibarbatum* Maximowicz | *R. semibarbatum* Maximowicz | 2*x* | 26 | [12, 13] |
| *Pentanthera* (11/29/41; 4 sections) | *Pentanthera* (7/20/20) | \ | *R. bakeri* | *R.* × *bakeri* (Lemmon & McKay) Hume (1948) (cultivation source) |  | 26 | [33] |
|  |  |  | *R. alabamense* | *R. alabamense* Rehder | 2*x* | 26 | [12, 17, 33-35] |
|  |  |  | *R. arborescens* | *R. arborescens* (Pursh) Torr. | 2*x* | 24, 26 | [10, 12, 13, 17, 29, 30, 33-35] |
|  |  |  | *R. atlanticum* (Ashe) Rehd. | *R. atlanticum* (Ashe) Rehder | 3*x*, 4*x* | 26 | [12, 13, 17, 30, 34, 35] |
|  |  |  | *R. austrinum* | *R. austrinum* (Small) Rehder | 2*x*, 3*x*, 4*x* | 26 | [13, 17, 33-35] |
|  |  |  | *R. calendulaceum* | *R. calendulaceum* (Michx.) Torr. | 3*x*, 4*x* | 24, 52 | [10, 12, 16, 17, 29, 30, 33, 35] |
|  |  |  | *R. canescens/R. roseum* | *R. canescens* (Michx.) Sweet | 2*x* | 26 | [10, 12, 13, 30, 33, 35] |
|  |  |  | *R. colemanii R. Miller* | *R. colemanii* R. Miller | 4*x* |  | [35] |
|  |  |  | *R. cumberlandense* | *R. cumberlandense* E. L. Braun | 2*x* | 26 | [13, 17, 33, 35] |
|  |  |  | *R. eastmanii* | *R. eastmanii* Kron & Creel | 2*x* |  | [17, 35] |
|  |  |  | *R. flammeum* | *R. flammeum* (Michx.) Sarg. | 2*x*, 4*x* | 26 | [17, 32, 35] |
|  |  |  | *R. japonicum/R. metternichii* | *R. japonicum* |  | 26 | [10, 12, 30] |
|  |  |  | *R. luteum* Sweet*/R. flavum* G. Don | *R. luteum* Sweet | 4*x* | 26 | [10, 12, 13, 35, 36] |
|  |  |  | *R. molle* | *R. molle* (Blume) G.Don | 2*x* | 26 | [10, 12, 35] |
|  |  |  | *R. mortieri* | *R*. × *mortieri* (Sweet) ? (*R. calendulaceum* × *periclymenoides*) (cultivation source) |  | 26 | [10] |
|  |  |  | *R. japonicum* Sur. | *R. molle* (Blume) G.Don ssp. *japonicum* (A.Gray) Kron |  | 26 | [37] |
|  |  |  | *R. occidentale* | *R. occidentale* (Torr. & A. Gray) A. Gray | 2*x* | 26, 52, 78 | [10, 12, 13, 17, 32, 35, 38] |
|  |  |  | *R. periclymenoides/R. nudiflorum* | *R. periclymenoides* (Michx.) Shinners | 2*x* | 26 | [12, 13, 17, 33, 35] |
|  |  |  | *R. prinophyllum* | *R. prinophyllum* (Small) Millais | 2*x* | 26 | [13, 17, 35] |
|  |  |  | *R. prunifolium* | *R. prunifolium* (Small) Millais | 2*x* | 26 | [12, 13, 17, 33, 35] |
|  |  |  | *R. serrulatum* | *R. serrulatum* (Small) Millais | 2*x* | 26 | [12, 17] |
|  |  |  | *R. viscosum/R. oblongifolium/R. serrulatum* | *R. viscosum* (L.) Torr. | 2*x* | 26 | [10, 12, 13, 17, 30, 33, 35] |
|  | *Rhodora* (1/2/2) | \ | *R. canadense* | *R. canadense* (L.) Torr. | 2*x* | 52 | [10, 12, 13, 16, 30, 35, 39] |
|  |  |  | *R. vaseyi* | *R. vaseyi* A. Gray | 2*x* | 26 | [10, 12, 13, 17, 30] |
|  | *Sciadorhodion* (3/6/18) | \ | *R. albrechtii* | *R. albrechtii* Maxim. | 2*x* | 26 | [12, 13, 34] |
|  |  |  | *R. benhallii Craven* var. *purpurea* (*Menziesia ciliicalyx* var. *purpurea*) | *R. benhallii* Craven var. *purpurea* (*Menziesia ciliicalyx* var. *purpurea*) | 4*x* |  | [13] |
|  |  |  | *R. pentaphyllum* | *R. pentaphyllum* Maxim. |  | 26 | [12] |
|  |  |  | *R. pilosum* Craven (*Menziesia pilosa* (Michaux) Juspieu) | *R. pilosum* (Michx.) Craven | 4*x* |  | [13] |
|  |  |  | *R. quinquefolium* | *R. quinquefolium* Bisset & S.Moore |  | 26 | [12] |
|  |  |  | *R. schlippenbachii* | *R. schlippenbachii* Maxim. | 2*x*, 3*x* | 26 | [9, 10, 12, 13, 25, 28, 30] |
|  | *Viscidula* (0/1/1) | \ | *R. nipponicum* | *R. nipponicum* Matsum. |  | 26 | [12] |
| *Rhododendron* (57/159/309; 2 sections) | *Pogonanthum* (1/8/26) | \ | *R. anthopogon* D. Don | *R. anthopogon* D. Don | 2*x* | 26 | [12, 13] |
|  |  | \ | *R. adamsii* Rehder | *R. fragrans* (Adams) Maxim. | 3*x* | 26, 55-58 | [28, 40-43] |
|  |  | \ | *R. cephalanthum/R. crebreflorum* | *R. cephalanthum* Franch. (ssp. *cephalanthum*) |  | 26 | [12] |
|  |  | \ | *R. kongboense* | *R. kongboense* Hutch. |  | 26 | [12] |
|  |  | \ | *R. sargentianum* | *R. sargentianum* Rehder & E.H.Wilson |  | 26 | [12] |
|  |  | \ | *R. temoense* | *R. laudandum* Cowan var. *temoense* Kingdon-Ward ex Cowan & Davidian |  | 26 | [12] |
|  |  | \ | *R. trichostomum/R. ledoides/R. radinum/R. sphaeranthum* | *R. trichostomum* Franch. |  | 26 | [12] |
|  |  | \ | *R. tsarongense* | *R. primuliflorum* Bureau & Franch. |  | 26 | [12] |
|  | *Rhododendron* [56/151/(287 - 4 unplaced) = 56/151/283; 28 subsections] | *Baileya* (1/1/1) | *R. baileyi* | *R. baileyi* Balf.f. | 2*x* | 52 | [12, 13] |
|  |  | *Boothia* (0/4/8) | *R. boothii* Nutt. | *R. boothii* Nutt. |  | 26 | [12] |
|  |  |  | *R. chrysodoron* | *R. chrysodoron T*agg ex Hutch. |  | 26 | [12] |
|  |  |  | *R. leucaspis* Tagg | *R. leucaspis* Tagg | 2*x* | 26 | [12, 13] |
|  |  |  | *R. megeratum* | *R. megeratum Balf.f.* |  | 26 | [12] |
|  |  | *Camelliiflora* (0/1/1) | *R. lucidum* Nutt./*R. camelliiflorum* Hook. f | *R. camelliiflorum Hook.* |  | 26 | [12] |
|  |  | *Campylogyna* (0/1/2) | *R. campylogynum/R. myrtilloides* | *R. campylogynum Franch.* |  | 26 | [12] |
|  |  | *Caroliniana* (0/1/2) | *R. minus/R. carolinianum* | *R. minus Michx.* | 2*x* | 24, 26 | [10, 12, 13, 29, 30, 32] |
|  |  | *Cinnabarina* (3/3/8) | *R. cinnabarinum* Hook. f. *(var. roylei; var. blandfordiaeflorum)* | *R. cinnabarinum Hook. (ssp. cinnabarinum)* | 6*x* | 78 | [12, 13, 16] |
|  |  |  | *R. xanthocodon/R. concatenans* | *R. cinnabarinum Hook.f. ssp. xanthocodon (Hutch.)* (Ref: Cullen, 1980) |  | 78 | [12, 16] |
|  |  |  | *R. keysii Nutt.* | *R. keysii Nutt.* |  | 78 | [12] |
|  |  | *Edgeworthia* (0/3/3) | *R. edgeworthii* Hook. f./*R. bullatum* | *R. edgeworthii Hook.* |  | 26 | [12] |
|  |  |  | *R. pendulam* Hook. f. | *R. pendulum Hook. f.* | 2*x* | 26 | [12-14] |
|  |  |  | *R. seinghkuense* | *R. seinghkuense Kingdon-Ward* |  | 26 | [12] |
|  |  | *Genestieriana* (0/1/1) | *R. genestierianum* Forrest | *R. genestierianum Forrest* | 2*x* |  | [13] |
|  |  | *Glauca* (1/8/11) | *R. brachyanthum* | *R. brachyanthum Franch.* |  | 26 | [12] |
|  |  |  | *R. charitostreptum*/*R. hypolepidotum* | *R. brachyanthum* Franch. ssp. *hypolepidotum* (Franch.) Cullen |  | 26 | [12] |
|  |  |  | *R. charitopes* | *R. charitopes* Balf.f. & Farrer |  | 26 | [12] |
|  |  |  | *R. charitopes* ssp. *tsangpoense/R. tsangpoense* | *R. charitopes* Balf.f. & Farrer ssp. *tsangpoense* (Kingdon-Ward) Cullen | 2*x* | 52 | [12, 13, 16] |
|  |  |  | *R. curvistylum* | *R. charitopes* ssp. *tsangpoense* (Kindon-Ward) Cullen × *campylogynum* Franch. |  | 26 | [12] |
|  |  |  | *R. glaucophyllum* | *R. glaucophyllum* Rehder |  | 26 | [12] |
|  |  |  | *R. glaucum* Hook. f. | *R. glaucophyllum* Rehder ssp. *glaucophyllum* var. *glaucophyllum* |  | 26 | [12] |
|  |  |  | *R. pruniflorum* | *R. pruniflorum* Hutch. |  | 26 | [12] |
|  |  | *Heliolepida* (4/4/8) | *R. bracteatum* Rehder & Wilson | *R. bracteatum* Rehder & Wilson | 6*x* |  | [13] |
|  |  |  | *R. heliolepis/R. aporinum*(correction: *R. oporinum*)/*R. heliolepis* var. *fumidum* (Balfour & Smith) Fang | *R. heliolepis* Franch. (var. *heliolepis*) | 6*x* | 52, 78 | [12, 13, 16] |
|  |  |  | *R. brevistylum/R. pholidotum* | *R. heliolepis* Franch. var. *brevistylum* (Franch.) Cullen |  | 52, 104 | [12, 16] |
|  |  |  | *R. rubiginosum/R. desquamatum* | *R. rubiginosum* Franch. | 6*x* | 52, 78 | [12, 13, 16] |
|  |  | *Lapponica* (17/27/52) | *R. capitatum* | *R. capitatum* Maxim. |  | 52 | [12, 16] |
|  |  |  | *R. complexum* | *R. complexum* Balf. & W. W. Sm. |  | 78 | [12, 16] |
|  |  |  | *R. cuneatum/R. ravum* | *R. cuneatum* W. W. Sm. | 6*x* | 52, 78 | [12, 13, 16] |
|  |  |  | *R. dasypetalum* | *R. dasypetalum* Balf. & Forrest |  | 52 | [12, 16] |
|  |  |  | *R. edgarianum* | *R. edgarianum* Rehder & E. H. Wilson |  | 52 | [12, 16] |
|  |  |  | *R. fastigiatum* | *R. fastigiatum* Franch. |  | 26, 52 | [12, 16] |
|  |  |  | *R. flavidum* | *R. flavidum* Franch. |  | 26, 52, 78 | [12, 16] |
|  |  |  | *R. fimbriatum* | *R. hippophaeoides* Balf.f. & W.W.Sm. var. *hippophaeoides* |  | 26 | [12] |
|  |  |  | *R. hippophaeoides* | *R. hippophaeoides* Balf.f. & W.W.Sm. |  | 26 | [12] |
|  |  |  | *R. impeditum/R. litangense* | *R. impeditum* Balf.f. & W.W.Sm. |  | 26 | [12] |
|  |  |  | *R. intricatum* | *R. intricatum* Franch. |  | 26, 52 | [12, 16] |
|  |  |  | *R. lapponicum* (L.) Wahlenb./*R. parvifolium* Adams/*R. parvifolium* Adams subsp. *alpinum* (Glehn) Dostalek | *R. lapponicum* (L.) Wahlenb. | 2*x* | 26, 52 | [12, 16, 28, 43-49] |
|  |  |  | *R. lysolepsis* | *R*. × *lysolepsis* Hutch. (1930) = *R. flavidum* × ? (cultivation source) |  | 26, 52 | [12, 16] |
|  |  |  | *R. ramosissimum/R. violaceum/R. stictophyllum* | *R. nivale* subsp. *boreale* Philipson & M. N. Philipson |  | 26, 52 | [12, 16] |
|  |  |  | *R. paludosum* | *R. nivale* Hook.f. *ssp.* nivale |  | 26 | [12] |
|  |  |  | *R. orthocladum* | *R. orthocladum* Balf.f. & Forrest |  | 26 | [12] |
|  |  |  | *R. microleucum* | *R. orthocladum* Balf.f. & Forrest var. *microleucum* (Hutch.) M.N.Philipson & Philipson | | 26 | [12] |
|  |  |  | *R. polycladum* Franchet/*R. scintillans/R. compactum* | *R. polycladum* Franchet | 2*x* | 26, 52 | [12, 13] |
|  |  |  | *R. rupicola/R. achroanthum* | *R. rupicola* W. W. Sm. |  | 26, 52 | [12, 16] |
|  |  |  | *R. chryseum* | *R. rupicola* W.W.Sm. var. *chryseum* (Balf.f. & Kingdon-Ward) M.N.Philipson & Philipson |  | 26 | [12] |
|  |  |  | *R. russatum* | *R. russatum* Balf. & Forrest |  | 39, 52 | [12, 16] |
|  |  |  | *R. setosum* D. Don | *R. setosum* D. Don | 2*x* | 26 | [12-14] |
|  |  |  | *R. tapetiforme* | *R. tapetiforme* Balf. & Kingdon-Ward | 4*x* | 78 | [12, 13, 16] |
|  |  |  | *R. drumonium/R. idoneum* | *R. telmateium* Balf. & W. W. Sm. |  | 26, 52, 78 | [12, 16] |
|  |  |  | *R. polifolium/R. spilanthum* | *R. thymifolium* Maxim. |  | 26 | [12] |
|  |  |  | *R. websterianum* | *R. websteranum* Rehder & E.H.Wilson |  | 26 | [12] |
|  |  |  | *R. yungingense/R. glomerulatum* | *R. yungningense* Balf. ex Hutch. |  | 26, 52 | [12, 16] |
|  |  | *Ledum* (1/3/8) | *R. columbianum* | *R. columbianum* (Piper) Harmaja |  | 26 | [32] |
|  |  |  | *R. groenlandicum* | *R. groenlandicum* (Oeder) Kron & Judd |  | 26 | [32] |
|  |  |  | *R. tomentosum* | *R. tomentosum* (Stokes) Harmaja | 4*x* | 26,52 | [13, 32, 50] |
|  |  | *Lepidota* (0/1/5) | *R. lepidotum* Wall. ex G. Don/*R. obovatum* Hook. f | *R. lepidotum* Wall. ex G. Don |  | 26 | [12] |
|  |  | *Maddenia* (4/39/65) | *R. burmanicum* | *R. burmanicum* Hutch. |  | 26 | [6] |
|  |  |  | *R. carneum* Hutchinson | *R. carneum* Hutchinson | 4*x* | 26 | [6, 12, 13] |
|  |  |  | *R. ciliatum* Hook. f. | *R. ciliatum* Hook. |  | 26 | [6, 10, 12] |
|  |  |  | *R. ciliicalyx* | *R. ciliicalyx* Franch. |  | 26 | [6, 12] |
|  |  |  | *R. coxianum* | *R. coxianum* Davidian |  | 26 | [6] |
|  |  |  | *R. crenulatum* | *R. crenulatum* Hutch. ex Sleumer |  | 26 | [6] |
|  |  |  | *R. cuffeanum* | *R. cuffeanum* Hutch. |  | 26 | [12] |
|  |  |  | *R. dalhousiae* Hooker (var. *dalhousiae*) | *R. dalhousiae* Hooker (var. *dalhousiae*) | 2*x* | 26 | [6, 12, 13] |
|  |  |  | *R. dalhousiae* var. *rhabdotum* | *R. dalhousiae* Hook.f. var. *rhabdotum* (Baff.f. & R.E.Cooper) Cullen |  | 26 | [6] |
|  |  |  | *R. dendricola* | *R. dendricola* Hutch. |  | 26 | [6, 12] |
|  |  |  | *R. excellens* | *R. excellens* Hemsl. & E.H.Wilson |  | 26 | [6] |
|  |  |  | *R. fletcheranum* | *R. fletcherianum* Davidian |  | 26 | [6] |
|  |  |  | *R. fleuryi* | *R. fleuryi* Dop |  | 26 | [6] |
|  |  |  | *R. formosum* Wall. | *R. formosum* Wall. |  | 26 | [6, 12] |
|  |  |  | *R. inaequale* | *R. formosum* Wall. var. *inaequale* (Hutch.) Cullen |  | 26 | [6, 12] |
|  |  |  | *R. formosum* var. *iteaphyllum* | *R. iteaphyllum* Hutch. |  | c.26 | [6] |
|  |  |  | *R. horlickianum* | *R. horlickianum* Davidian |  | 26 | [6] |
|  |  |  | *R. johnstoneanum* Watt. | *R. johnstoneanum* Watt ex Hutch. |  | 26 | [6, 12] |
|  |  |  | *R. leptocladon* | *R. leptocladon Dop* |  | 26 | [6] |
|  |  |  | R. liliiflorum | Rhododensron liliiflorum *H.Lév.* |  | 26 | [6] |
|  |  |  | *R. lindleyi Moore* | *R. lindleyi* Moore | 2*x* | 26 | [6, 12, 13] |
|  |  |  | *R. ludwigianum* | *R. ludwigianum* Hosseus |  | 26 | [6] |
|  |  |  | *R. lyi* | *R. lyi* H.Lév. |  | 26 | [6, 12, 51] |
|  |  |  | *R. maddenii* (ssp. *maddenii*)/*R. polyandrum* Hutch./*R. calophyllum* Nutt. | *R. maddenii* Hook. f. (ssp. *maddenii*) | 2*x*, 6*x*, 8*x* | 26, 52, 78 | [6, 12-14, 16, 17] |
|  |  |  | *R. maddenii* ssp. *crassum*/R*. crassum*/R*. manipurense* | *R. maddenii* Hook.f. ssp. *crassum* (Franch.) Cullen | 6*x*, 8*x* | 52, 78, 104, 156 | [6, 12, 16, 17] |
|  |  |  | *R. megacalyx* | *R. megacalyx* Balf.f. |  | 26 | [6, 12] |
|  |  |  | *R. nuttallii* | *R. nuttallii* Booth |  | 26 | [6, 12] |
|  |  |  | *R. parryae* Hutch. | *R. parryae* Hutch. |  | 26 | [6, 12] |
|  |  |  | *R. pachypodum*/R. *scottianum*/R. *supranubium* | *R. pachypodum* Balf.f. & W.W.Sm. |  | 26 | [6, 12] |
|  |  |  | *R. roseatum* | *R. roseatum* Hutch. |  | 26 | [6] |
|  |  |  | *R. scopulorum* | *R. scopulorum* Hutch. |  | 26 | [6, 12] |
|  |  |  | *R. sinonuttallii* | *R. sinonuttallii* Balf.f. & Forrest |  | 26 | [6] |
|  |  |  | *R. taggianum* | *R. taggianum* Hutch. |  | 26 | [12] |
|  |  |  | *R. taronense* | *R. taronense* Hutch. |  | c.104 | [6] |
|  |  |  | *R. valentinianum* | *R. valentinianum* Forrest ex. Hutch. |  | 26 | [6, 12] |
|  |  |  | *R. vanderbiltianum* Merr. | *R. vanderbiltianum* Merr. |  | 26 | [52] |
|  |  |  | *R. veitchianum*/*R. cubittii* | *R. veitchianum* Hook.f. |  | 26 | [6, 12] |
|  |  |  | *R. walongense* | *R. walongense* Kingdon-Ward |  | 26 | [6] |
|  |  |  | *R. yungchangense* | *R. yungchangense* Cullen |  | 26 | [6] |
|  |  | *Micrantha* (0/1/3) | *R. micranthum* Turczaninow | *R. micranthum* Turcz. | 2*x* | 26 | [12, 13] |
|  |  | *Monantha* (0/1/4) | *R. flavantherum* | *R. flavantherum* Hutch. & Kingdon-Ward |  | 26 | [12] |
|  |  | *Moupinensia* (0/1/3) | *R. moupinense* Franchet | *R. moupinense* Franchet | 2*x* | 26 | [12, 13] |
|  |  | *Rhododendron* (0/3/3) | *R. ferrugineum* L. | *R. ferrugineum* L. | 2*x* | 26 | [10, 12, 13] |
|  |  |  | *R. hirsutum* L. | *R. hirsutum* L. | 2*x* | 26 | [12, 13, 53] |
|  |  |  | *R. kotschyi* Simonk. | *R. myrtifolium* Schott & Kotschy |  | 26 | [54] |
|  |  | *Rhodorastra* (4/4/7) | *R. da(h)uricum* L. | *R. dauricum* L. | 3*x* | 26 | [10, 12, 20, 27, 30, 55] |
|  |  |  | *R. ledebourii* | *R. ledebourii* Pojark. | 3*x* |  | [55] |
|  |  |  | *R. mucronulatum* Turcz. | *R. mucronulatum* Turcz. | 4*x*, 5*x* | 26 | [10, 12, 13, 20, 55] |
|  |  |  | *R. sichotense* Pojark. | *R. sichotense* Pojark. | 2*x*, 6*x* | 26 | [13, 20, 25, 27, 55] |
|  |  | *Saluenensia* (4/6/8) | *R. calostrotum* | *R. calostrotum* Balf.f. & Kingdon-Ward |  | 26 | [12] |
|  |  |  | *R. keleticum/R. radicans* | *R. calostrotum* Balf.f. & Kingdon-Ward ssp. *keleticum* (Balf.f. & Forrest) Cullen |  | 26 | [12] |
|  |  |  | *R. calciphilum/R. riparium* Ward*/R. nitens* | *R. calostrotum* Balf.f. & Kingdon-Ward ssp. *riparium* (Kingdon-Ward) Cullen |  | 26, 52 | [12, 16] |
|  |  |  | *R. saluenense* | *R. saluenense* Franch. |  | 26, 52 | [12, 16] |
|  |  |  | *R. cosmetum/R. chameunum/R. charidotes* | *R. saluenense* Franch. ssp. *chameunum* (Balf.f. & Forrest) Cullen |  | 26, 52 | [12, 16] |
|  |  |  | *R. prostratum* | *R. saluenense* var. *prostratum* (W. W. Sm.) R. C. Fang |  | 52 | [12, 16] |
|  |  | *Scabrifolia* (0/7/11) | *R. hemitrichotum* | *R. hemitrichotum* Balf.f. & Forrest |  | 26 | [12] |
|  |  |  | *R. mollicomum* | *R. mollicomum* Balf.f.& W.W.Sm. |  | 26 | [12] |
|  |  |  | *R. pubescens* | *R. pubescens* Balf.f. & Forrest |  | 26 | [12] |
|  |  |  | *R. racemosum* Franchet | *R. racemosum* Franchet | 2*x* | 26 | [12, 13] |
|  |  |  | *R. scabrifolium* | *R. scabrifolium* Franch. |  | 26 | [12] |
|  |  |  | *R. spiciferum* Franchet | *R. spiciferum* Franchet | 2*x* | 26 | [12, 13] |
|  |  |  | *R. spinuliferum* Franchet | *R. spinuliferum* Franchet | 2*x* | 26 | [12, 13] |
|  |  | *Tephropepla* (0/3/10) | *R. auritum* Tagg | *R. auritum* Tagg | 2*x* | 26 | [12, 13] |
|  |  |  | *R. tephropeplum* | *R. tephropeplum* Balf.f. & Farrer |  | 26 | [12] |
|  |  |  | *R. xanthostephanum* Merrill | *R. xanthostephanum* Merrill | 2*x* | 26 | [12, 13] |
|  |  | *Triflora* (16/22/40) | *R. ambiguum* Hemsl. | *R. ambiguum Hemsl.* | 6*x* | 52 | [12, 13, 16] |
|  |  |  | *R. amesiae* | *R. amesiae* Rehder & E. H. Wilson | 4*x* | 52 | [12, 13] |
|  |  |  | *R. augustinii* | *R. augustinii* Hemsl. | 4*x* | 52 | [12, 16, 17] |
|  |  |  | *R. augustinii ssp. chasmanthum* Cullen*/R. chasmanthum* | *R. augustinii* ssp. *chasmanthum* Cullen/*R. chasmanthum* | 4*x* | 52 | [12, 13, 16] |
|  |  |  | *R. concinnum/R. pseudoyanthinum* | *R. concinnum* Hemsl. | 2*x*, 6*x* | 52 | [12, 13, 16] |
|  |  |  | *R. davidsonianum/R. charianthum* | *R. davidsonianum* Rehder & E. H. Wilson | 4*x* | 52, 78 | [12, 13, 16] |
|  |  |  | *R. keiskei* Miquel | *R. keiskei* Miquel | 2*x* | 26 | [10, 12, 13] |
|  |  |  | *R. keiskei* var. *hypoglaucum* | *R. keiskei* Miq. var. *hypoglaucum* Sutô & Suzuki |  | 26 | [56] |
|  |  |  | *R. lutescens* Franchet | *R. lutescens* Franchet | 2*x* | 26 | [12, 13] |
|  |  |  | *R. oreotrephis/R. artosquamatum/R. exquisitum/R. timeteum* | *R. oreotrephes* W. W. Sm. |  | 52,78 | [12, 16] |
|  |  |  | *R. pleistanthum* Balfour ex Wilding | *R. pleistanthum* Balfour ex Wilding | 4*x* |  | [13] |
|  |  |  | *R. polylepis* Franchet | *R. polylepis* Franchet | 2*x* | 26 | [12, 13] |
|  |  |  | *R. searsiae* | *R. searsiae* Rehder & E. H. Wilson |  | 52 | [12, 16] |
|  |  |  | *R. siderophyllum* Franch. | *R. siderophyllum* Franch. | 6*x* | 78 | [12, 13, 16] |
|  |  |  | *R. trichanthum* Rehder | *R. trichanthum* Rehder | 6*x* |  | [13] |
|  |  |  | *R. triflorum* Hook. f. | *R. triflorum* Hook. f. | 2*x*, 6*x* | 26 | [12-14] |
|  |  |  | *R. bauhiniiflorum* Watt. ex Hutch | *R. triflorum* var. *bauhiniiflorum* (Watt) C. I. Cullen & D. F. Chamb. |  | 26 | [12] |
|  |  |  | *R. yunnanense/R. aechmophyllum/R. chartophyllum/R. suberosum* | *R. yunnanense* Franch. | 4*x* | 26, 52, 78 | [12, 13, 16] |
|  |  |  | *R. zaleucum* | *R. zaleucum* Balf. & W. W. Sm. |  | 52 | [12, 16] |
|  |  |  | *R. zaleucum* var. *flaviflorum* Balfour & Smith | *R. zaleucum* var. *flaviflorum* Balfour & Smith | 2*x* |  | [13] |
|  |  |  | *R. rigidum* Franchet | *R. rigidum* Franchet | 4*x* | 26 | [12, 13] |
|  |  |  | *R. tatsienense* Franchet | *R. tatsienense* Franchet | 6*x* |  | [13] |
|  |  | *Trichoclada* (0/2/9) | *R. chloranthum* | *R. mekongense* Franch. *Mekongense* |  | 26 | [12] |
|  |  |  | *R. trichocladum* | *R. trichocladum* Franch. |  | 26 | [12] |
|  |  | *Uniflora* (1/2/5) | *R. imperator* | *R. uniflorum* Hutch. & Kingdon-Ward var. *imperator* (Kingdon-ward) Cullen |  | 26 | [12] |
|  |  |  | *R. pemakoense/R. patulum* | *R. pemakoense* Kingdon-Ward |  | 26, 52 | [12, 16] |
|  |  | *Virgata* (0/2/3) | *R. oleifolium* | *R. virgatum* Hook.f. ssp. *oleifolium* (Franch.) Cullen |  | 26 | [12] |
|  |  |  | *R. virgatum* Hook. f. | *R. virgatum* Hook. |  | 26 | [12] |
| *Therorhodion* (0/3/3) | \ | \ | *R. camtschaticum* Pall. (ssp. *camtschaticum*) | *R. camtschaticum* Pall. (ssp. *camtschaticum*) | 2*x* | 24, 26 | [12, 13, 39, 57-59] |
|  |  |  | *Therorhodion glandulosum* | *R. camtschaticum* var. *glandulosum* (Standl.) B. Boivin |  | 24,26 | [32] |
|  |  |  | *R. redowskianum* Maxim. | *R. redowskianum Maxim.* |  | 26 | [40] |
| *Tsutsusi* (0/22/148; 2 sections) | *Brachycalyx* (0/5/34) | \ | *R. amagianum* | *R. amagianum* (Makino) Makino ex H. Hara |  | 26 | [12] |
|  |  |  | *R. dilatatum* Miquel | *R. dilatatum* Miquel | 2*x* |  | [13] |
|  |  |  | *R. mariesii* | *R. mariesii* Hemsl. & E.H.Wilson |  | 26 | [12] |
|  |  |  | *R. reticulatum* | *R. reticulatum* D.Don (1834) |  | 26 | [10, 12, 30] |
|  |  |  | *R. weyrichii* | *R. weyrichii* Maxim. |  | 26 | [12] |
|  | *Tsutsusi* (0/17/114) | \ | *R. eriocarpum* (Hayata) Nakai/*R. phoeniceum* var. *eriocarpum* | *R. eriocarpum* (Hayata) Nakai | 2*x* | 26 | [12, 13] |
|  |  |  | *R. indicum/R. macranthum* G. Don*/R. eriocarpum* | *R. indicum* (L.) Sweet | 2*x* | 26 | [9, 10, 12, 60, 61] |
|  |  |  | *R. pulchrum* | *R. indicum* × 'Ledifolia' (cultivation source) |  | 26 | [12] |
|  |  |  | *R. kaempferi* | *R. kaempferi* Planch. | 2*x* | 26 | [9, 13] |
|  |  |  | *R. kiusianum* | *R. kiusianum* Makino | 2*x* | 26 | [9, 60] |
|  |  |  | *R. mucronatum/R. ledifolium* | *R. mucronatum* (Blume) G.Don | 2*x* | 26 | [10, 12, 60] |
|  |  |  | *R. mucronatum* (Blume) G. Don var. *ripense* (Makino) Wilson | *R. mucronatum* (Blume) G. Don var. *ripense* (Makino) Wilson | 2*x* |  | [13] |
|  |  |  | *R. nakaharai Hay* | *R. nakaharai* Hayata |  | 26 | [62] |
|  |  |  | *R. noriakianum* | *R. noriakianum* Suzuki | 2*x* |  | [60] |
|  |  |  | *R. oldhamii* Maximowicz | *R. oldhamii* Maximowicz | 2*x* | 26 | [12, 13] |
|  |  |  | *R. scabrum* | *R. scabrum* G.Don | 2*x* |  | [60] |
|  |  |  | *R. simsii Planch.* | *R. simsii* Planch. | 2*x* | 26 | [60, 63] |
|  |  |  | *R. linearifolium* (var. *macrosepalum*)/*R. hortense* | *R. stenopetalum* (Hogg) Mabb. |  | 26 | [10, 12] |
|  |  |  | *R. stenopetalum* ‘Linearifolium’ | *R. stenopetalum* (Hogg) Mabb. ‘Linearifolium’ (cultivation source) | 2*x* | 26 | [12, 17] |
|  |  |  | *R. tashiroi* Maximowicz | *R. tashiroi* Maximowicz | 2*x* |  | [13] |
|  |  |  | *R. tosaense* | *R. tosaense* Makino |  | 26 | [12] |
|  |  |  | *R. tschonoskii* | *R. tschonoskii* Maxim. |  | 26 | [12] |
|  |  |  | *R. yedoense* | *R. yedoense* Maxim. |  | 26 | [10, 12] |
|  |  |  | *R. yedoense poukhanense* | *R. yedoense* var. *poukhanense* (H.Lév.) Nakai |  | 26 | [30] |
| *Vireya* (16/49/407; 7 sections) | *Albovireya* (1/2/16) | \ | *R. aequabile* J.J. Sm. | *R. aequabile* J. J. Sm. |  | 26 | [51, 52] |
|  |  |  | *R. album* Blume | *R. album* Blume | 4*x* | 26 | [13, 52] |
|  | *Discovireya* (0/4/41) | \ | *R. cuneifolium* Stapf | *R. cuneifolium* Stapf |  | c.26 | [52] |
|  |  |  | *R. gaultheriifolium* J.J.S. | *R. gaultheriifolium* J. J. Sm. |  | 26 | [51] |
|  |  |  | *R. quadrasianum* Vidal | *R. quadrasianum* S. Vidal |  | 26 | [52] |
|  |  |  | *R. retusum* | *R. retusum* (Blume) Benn. | 2*x* | 26 | [12, 13] |
|  | *Hadranthe* (Phaeovireya) (2/5/54) | \ | *R. beyerinckianum* Koord. | *R. beyerinckianum* Koord. |  | 26 | [51, 52] |
|  |  |  | *R. dianthosmum* Sleumer | *R. dianthosmum* Sleumer |  | 26 | [52] |
|  |  |  | *R. konori* | *R. konori* Becc. | 4*x* | 26 | [13, 51] |
|  |  |  | *R. phaeopeplum* Sleum. | *R. konori* Becc. var. *phaeopeplum* (Sleumer) Argent |  | 26 | [51] |
|  |  |  | *R. phaeochitum* F. Muell. | *R. phaeochitum* F. Muell. | 4*x* | 26 | [13, 52] |
|  |  |  | *R. caliginis* Kores × *R. vitis-idaea* Sleumer | *R. caliginis* Kores × *R. vitis-idaea* Sleumer (cultivation source) |  | 26 | [52] |
|  | *Malayovireya* (1/3/21) | \ | *R. acuminatum* Hook. f. | *R. acuminatum* Hook. |  | 26 | [51] |
|  |  |  | *R. apoanum* Stein | *R. apoanum* Stein | 4*x* | 26 | [13, 52] |
|  |  |  | *R. himantodes* Sleumer | *R. himantodes* Sleumer |  | 26 | [52] |
|  | *Pseudovireya* (1/5/17) | \ | *R. emarginatum* | *R. emarginatum* Hemsl. & E.H.Wils. | 4*x* |  | [13] |
|  |  |  | *R. kawakamii* Hayata | *R. kawakamii* Hayata (1911) | 2*x* |  | [13] |
|  |  |  | *R. rushforthii* Argent & D.F. Chamb. | *R. rushforthii* Argent & D. F. Chamb. | 2*x* | 26 | [13, 52] |
|  |  |  | *R. santapaui* Sastry & et al. | *R. santapaui* Sastry & al. |  | 26 | [52] |
|  |  |  | *R. vaccinioides* Hook. f. | *R. vaccinioides* Hook. |  | 26 | [12] |
|  | *Schistanthe* (11/28/244; 5 subsections) | *Euvireya* (7/11/121) | *R. aurigeranum* | *R. aurigernum* Sleumer | 4*x* |  | [13] |
|  |  |  | *R. christianae* Sleum. | *R. christianae* Sleumer |  | 26 | [51] |
|  |  |  | *R. crassifolium* Stapf | *R. crassifolium* Stapf |  | 26 | [52] |
|  |  |  | *R. javanicum* (ssp. *javanicum*) | *R. javanicum* (Blume) Benn. | 4*x* | 26 | [12, 13] |
|  |  |  | *R. laetum* J.J.S. | *R. laetum J*. J. Sm. | 4*x* | 26 | [13, 51] |
|  |  |  | *R. lochiae* F. Muell. | *R. lochiae* F.Muell. |  | 26 | [12, 51, 52] |
|  |  |  | *R. lochiae/viriolosum* (unidentified sample) | *R. lochiae/viriolosum* (unidentified sample) | 4*x* |  | [13] |
|  |  |  | *R. macgregoriae* | *R. macgregoriae* F. Muell. | 4*x* | 26 | [13, 51] |
|  |  |  | *R. orbiculatum* | *R. orbiculatum* Ridl. | 4*x* | 26 | [13, 51, 52] |
|  |  |  | *R. polyanthemum* | *R. polyanthemum* Sleumer | 4*x* |  | [13] |
|  |  |  | *R. praetervisum* Sleumer | *R. praetervisum* Sleumer |  | 26 | [52] |
|  |  |  | *R. verticillatum* | *R. verticillatum* Low ex Lindl. | 4*x* |  | [13] |
|  |  | *Linnaeopsis* (1/2/16) | *R. anagalliflorum* Wernham | *R. anagalliflorum* Wernham |  | 26 | [52] |
|  |  |  | *R. gracilentum* | *R. gracilentum* F. Meull. | 4*x* |  | [13] |
|  |  | *Malesia* (2/8/60) | *R. bagobonum* H.F. Copel. | *R. bagobonum* H. F. Copel. |  | 26 | [52] |
|  |  |  | *R. burtii* | *R. burtii* R.Woods | 4*x* |  | [13] |
|  |  |  | *R. buxifolium* Low ex Hook. f. | *R. buxifolium* H. Low ex Hook. |  | 26 | [51] |
|  |  |  | *R. commonae* Foerst. | *R. commonae* Foerst. |  | 26 | [51, 52] |
|  |  |  | *R. inconspicuum* J.J.S.*/R. invasorium* Sleum. | *R. inconspicuum* J.J.Sm. |  | 26 | [51] |
|  |  |  | *R. multicolor* Miq. | *R. multicolor* Miq. |  | 26 | [51] |
|  |  |  | *R. taxifolium* | *R. taxifolium* Merr. | 4*x* |  | [13] |
|  |  |  | *R. wrightianum* var. *cyclopense* J.J. Sm. | *R. wrightianum* var. *cyclopense* J.J. Sm. |  | 26 | [52] |
|  |  | *Solenovireya* (1/7/46) | *R. cruttwellii* | *R. crutwellii* Sleumer | 4*x* |  | [13] |
|  |  |  | *R. pneumonanthum* Sleumer | *R. edanoi* var. *pneumonanthum* |  | 26 | [52] |
|  |  |  | *R. jasminiflorum* | *R. jasminiflorum* Hook. |  | 26 | [12] |
|  |  |  | *R. loranthiflorum* Sleumer | *R. loranthiflorum* Sleumer |  | 26 | [52] |
|  |  |  | *R. ruttenii* J.J. Sm. | *R. ruttenii* J. J. Sm. |  | 26 | [52] |
|  |  |  | *R. stapfianum* Hemsl. ex Prain | *R. stapfianum* Hemsl. ex Prain |  | c.26 | [52] |
|  |  |  | *R. tuba* Sleumer | *R. tuba* Sleumer |  | 26 | [52] |
|  | *Siphonovireya* (0/2/14) | \ | *R. herzogii* Warb. | *R. herzogii* Warb. |  | 26 | [52] |
|  |  |  | *R. inundatum* Sleumer | *R. psammogenes* var. *inundatum* |  | 26 | [52] |

**References**

1. Chamberlain, D., et al., *The genus Rhododendron: its classification and synonymy*. 1996, Edinburgh, UK: Royal Botanic Garden Edinburgh.

2. Argent, G., *Rhododendron of subgenus Vireya*. 2nd ed. 2015, Edinburgh, UK: Royal Botanic Garden Edinburgh.

3. Argent, G., M. Möller, and A. Clark, *Current taxonomy-Rhododendron vanderbilitianum Merr.* Rhododendrons with Camellias and Magnolias, 2008: p. 100-102.

4. Donald, F., *A taxonomic review of the yellow-flowered species of Rhododendron L. subsection Maddenia (Hutch.) Sleumer*. 2012, The University of Edinburgh & Royal Botanic Garden Edinburgh Edinburgh, UK.

5. MacKay, M., *Vireyas from West and East: distribution and conservation of Rhododendron section Schistanthe.* Rhododendrons International, 2018. **2**: p. 138.

6. Cubey, J.J., *A cytological and morphological taxonomic study of Rhododendron L. subsections Saluenensia (Hutch.) Sleumer and Maddenia (Hutch.) Sleumer*. 2003, University of Liverpool: Liverpool, UK.

7. Mehra, P. and K. Bawa, *Chromosomal evolution in tropical hardwoods.* Evolution, 1969: p. 466-481.

8. Mehra, P.N., *Cytology of Himalayan hardwoods*. 1976, Calcutta, India: Sree Saraswaty Press Ltd.

9. Mitsukuri, Y. and I. Ishii, *Cytological studies on Ericaceae. I. Chromosome numbers and karyotypes of subgenus Rhododendron.* Japanese Journal of Genetics, 1966. **41**(4): p. 283-+.

10. Darlington, C.D. and E. Janaki Ammal, *Chromosome atlas of cultivated plants.* Chromosome atlas of cultivated plants., 1945.

11. Lianming, G., et al., *Chromosome numbers of some species of Rhododeodron, subgen. Azaleastrum.* Acta Botanica Yunnanica, 2005. **27**(4): p. 433-436.

12. Ammal, E.J., I. Enoch, and M. Bridgwater, *Chromosome numbers in species of Rhododendron.* Rhododendron year book, 1950. **5**: p. 78-91.

13. Khan, G., et al., *Incongruent phylogenies and its implications for the study of diversification, taxonomy and genome size evolution of Rhododendron.* American journal of botany, 2021. **108**(10): p. 1957-1981.

14. De, K.K., et al., *Investigation on relative genome sizes and ploidy levels of Darjeeling-Himalayan Rhododendron species using flow cytometer.* Indian Journal of Biotechnology, 2010. **9**: p. 64-68.

15. Cave, M.S., *Index to Plant Chromosome Numbers for 1957*. 1958, California Botanical Society: Berkeley.

16. Darlington, C.D. and A.P. Wylie, *Chromosome atlas of flowering plants*. 1955, London, UK: George Allen & Unwin Ltd.

17. Jones, J.R., et al., *Ploidy levels and relative genome sizes of diverse species, hybrids, and cultivars of Rhododendron*. 2007, Journal American Rhododendron Society. p. 220-227.

18. Mehra, P., *Cytogenetical evolution of hardwoods.* The Nucleus, 1972. **15**: p. 64-83.

19. Milne, R.I., S. TERZİOĞLU, and R.J. Abbott, *Origin and maintenance of Rhododendron × sochadzeae, a fertile F1 hybrid which occupies an ecotone between R. ponticum and R. caucasicum in Turkey.* Turkish Journal of Botany, 2004. **28**(1): p. 93-100.

20. Gurzenkov, N., *Studies of chromosome numbers of plants from the south of the Soviet Far East.* Komarov Lectures, 1973. **20**: p. 47-61.

21. Zhukova, P., *Chromosome numbers of some western Chukotka plant species.* Bot. Zhurn., 1975. **60**: p. 395-401.

22. Zhukova, P., *Chromosome numbers of some Southern Chukotka plant species.* Bot. Zhurn., 1980. **65**: p. 51-59.

23. Stepanov, N., *Chromosome numbers of some higher plant taxa of the flora of the Krasnoyarsk region.* Botanicheskiĭ Zhurnal, 1994. **79**(2): p. 135-139.

24. Probatova, N.S., et al., *Further chromosome studies on vascular plant species from Sakhalin, Moneran and Kurile Islands.* 北海道大学総合博物館研究報告, 2006. **3**: p. 93-110.

25. Marhold, K., *IAPT/IOPB chromosome data 5.* Taxon, 2008. **57**(2): p. 553-562.

26. Marhold, K. and J. Kucera, *IAPT/IOPB chromosome data 22.* Taxon, 2016. **65**(5): p. 1200-1207.

27. Andriyanova, E.A. and O.A. Mochalova, *IAPT/IOPB chromosome data 26.* Taxon, 2017. **66**(6): p. 1487-1499.

28. Zaytseva, Y.G., et al., *Flow cytometric determination of genome size and ploidy level of some frost-resistant cultivars and species of Rhododendron L. native to Asian Russia.* Botanica Pacifica: a Journal of Plant Science and Conservation, 2018. **7**(1): p. 97-100.

29. Bowers, C.G., *The development of pollen and viscin strands in Rhododendron catawbiense.* Bulletin of the Torrey Botanical Club, 1930. **57**(5): p. 285-313.

30. Sax, K., *Chromosome stability in the genus Rhododendron.* American Journal of Botany, 1930. **XVII**(4): p. 247-251.

31. Magulaev, A.V., *Cytotaxonomic study in some flowering plants of the North Caucasus.* Botaniceskjij Zurnal SSSR, 1984. **69**(4): p. 511-517.

32. *Flora of North America*. Available from: <http://floranorthamerica.org/Main_Page>.

33. Li, H.-L., *Chromosome studies in the azaleas of eastern North America.* American Journal of Botany, 1957. **44**(1): p. 8-14.

34. Fedorov, A., *Chromosome numbers of flowering plants.* Rept. Koenigstein., 1974.

35. Zhou, W.-Y., et al. *Rhododendron colemanii: A new species of deciduous azalea (Rhododendron section Pentanthera; Ericaceae) from the Coastal Plain of Alabama and Georgia*. 2008. **62**, 72-78.

36. Pogan, E., et al., *Further studies in chromosome numbers of Polish angiosperms. Part XIX.* Acta Biologica Cracoviensia. Series Botanica, 1986. **28**.

37. Kalninya, E.M. and S.V. Klivtsov., *Karyotype characteristics of Rhododendron japonicum Sur.* J. Biol. Sci. (Bombay), 1980. **12**: p. 62-64.

38. Niehaus, T. and J. L. Wong, *IOPB chromosome reports XXXII.* Taxon, 1971. **20**: p. 349-356.

39. Ornduff, R., *Index to plant chromosome numbers for 1965*. 1966, International Bureau for Plant Taxonomy and Nomenclature of the International Association for Plant Taxonomy.

40. Krogulevich, R., *The role of polyploidy in the genesis of the alpine flora of the Stanovoye Nagorye Mountains.* The ecology of flora of the Trans-Baikal region, 1971: p. 115-214.

41. Krogulevich, R., *Chromosome numbers of plant species from the Tunkinsky Alpes (East Sayan).* News Sib. Depart. Ac. Sci. USSR, Ser. Biol., 1976. **3**: p. 64-52.

42. Krogulevich, R., *Karyological analysis of the species of the flora of estern Sayan.* Flora of the Prebaikal, Novosibirsk, 1978: p. 19-48.

43. Yurtsev, B., *Chromosome numbers of some plants of the northeastern Yakutia (the Drainage of the Indigerka River in its middle reaches).* Bot. Zurn., 1982. **67**: p. 778-787.

44. Zhukova, P., *Chromosome numbers in some species of plants of the north-eastern part of the USSR II.* Bot. Zhur., 1967. **52**: p. 982-987.

45. Hagerup, O., *Morphological and cytological studies of Bicornes*. 1928: éditeur non identifié.

46. Murín, A., I. Háberová, and C. Žamsran, *Further karyological studies of the Mongolian flora.* Folia geobotanica et phytotaxonomica, 1984. **19**: p. 29-39.

47. Lantai, K. and B. Kihlman, *The chromosome numbers of Ledum palustre ssp. decumbens and of some related taxa.* Hereditas, 1995. **122**(2): p. 181-184.

48. Aguilera, P.M., J.R. Daviña, and A.I. Honfi, *IAPT/IOPB chromosome data 12.* Taxon, 2011. **60**(6): p. 1784-72E.

49. Rice, A., et al., *The Chromosome Counts Database (CCDB) – a community resource of plant chromosome numbers.* New Phytologist, 2015. **206**(1): p. 19-26.

50. A., M. and M. J., *Karyological study of Slovakian flora IV*. 1983, Acta Fac. Rerum Nat. Univ. Comen., Bot. p. 1-16.

51. Jones, K. and C. Brighton, *Chromosome numbers of tropical Rhododendrons.* Kew Bulletin, 1972. **26**(3): p. 559-561.

52. Atkinson, R., K. Jong, and G. Argent, *Chromosome numbers of some tropical rhododendrons (section Vireya).* Edinburgh Journal of Botany, 2000. **57**(1): p. 1-7.

53. Dobea, C., B. Hahn, and W. Morawetz, *Chromosome numbers of the vascular plants—Flora of Austria.* Linzer Biol. Beitr, 1997. **29**: p. 5-43.

54. Pashuk, K., *Chromosome numbers in species of subalpine belt of Chernogora (Ukrainian Carpatians).* Bot. Zurn., 1987. **72**: p. 1069-1074.

55. Zaytseva, Y.G., et al., *Flow cytometric determination of genome size and ploidy level of some frost-resistant cultivars and species of Rhododendron L. native to Asian Russia.* Botanica Pacifica, 2018. **7**(1): p. 97-100.

56. Choi, B., et al., *A study of the chromosome number and genome size of the rare species Rhododendron keiskei var. hypoglaucum in Korea.* Korean Journal of Plant Taxonomy, 2022. **52**(2): p. 102-107.

57. Ornduff, R., *Index to plant chromosome numbers.* Reg. Veg., 1968. **55**.

58. Cave, M.S., *Index to Plant Chromosome Numbers for 1963*. 1964, California Botanical Society: Berkeley.

59. Goldblatt, P. and D. Johnson. *Index to plant chromosome numbers (IPCN)*. Missouri Botanical Garden website 1979; Available from: <http://legacy.tropicos.org/project/ipcn>.

60. De Schepper, S., et al., *Flow cytometric analysis of ploidy in Rhododendron (subgenus Tsutsusi).* HortScience, 2001. **36**(1): p. 125-127.

61. Kumar, V., *Chromosome Atlas of Flowering Plants of the Indian Subcontinent.* Botanical Survey of India, 1987.

62. Hsu, C.-C., *Preliminary chromosome studies on the vascular plants of Taiwan (II).* Taiwania, 1968. **14**(1): p. 11-27.

63. Chen, R., *Chromosome atlas of major economic plants genome in China*. 2003: Science Press.
