# Supplementary material for: Ploidy variation in Rhododendron subsection Maddenia and its implications for conservation
Source: AoB Plants. 2023 Apr 12;15(3):plad016. doi: 10.1093/aobpla/plad016 (PMC10184449; doi:10.1093/aobpla/plad016)
Supplement: plad016_suppl_Supplementary_Table_S2 [file plad016_suppl_supplementary_table_s2.docx]

**Table S2.** Ploidy estimation of taxa in subsection *Maddenia* in the present study, using flow cytometry. For diploids (RN1 only), FCM ploidy = diploid standard (2*x*); for polyploids (RN1 and RN2), $FCM ploidy=\frac{Mean-x \left( RN2: polyploid peak \right)}{\mathrm{Mean}-x \left( RN1: standard or diploid peak \right)}\boldsymbol{\times}2$, round number. **1)** Sample IDs marked with an asterisk (*) were also evaluated using silica gel-dried and herbarium leaves (Tables 3 and S3). Sample IDs marked with a hashtag (#) were evaluated using both *R. fortunei* Lindl. and *R. parryae* Hutch. as diploid standards (Tables 4 and S3). Ploidy shown here was from the most reliable result. **2)** Voucher specimen ID of diploid standards: *R. fortunei*, MPN52690; *R. parryae*, MPN52773. **3)** Herbarium specimens for NZ accessions are deposited in the Dame Ella Campbell Herbarium (MPN) at Massey University, Palmerston North, New Zealand (listed with voucher specimen ID). Living plants of the accessions in the Royal Botanic Garden Edinburgh (RBGE), UK and Rhododendron Species Botanical Garden (RSBG), Washington, USA are accessible at the corresponding organizations (listed with living accession ID). When there is no specimen ID provided, the living accession can be traced on site, with the original collection number as a note to “Taxon labelled as”. **4)** When a sample accession is recorded as from the wild, it is noted as a “Wild accession” with original locality, when the information is available. Samples are otherwise from a cultivated source.

| **Sample** | **Taxon** | **Taxon labelled as** | **FCM ploidy** | **RN1** | | **RN2** | | **Diploid SD** | **Leaf sample** | **Wild accession** | **Specimen location and ID** |
| --- | --- | --- | --- | --- | --- | --- | --- | --- | --- | --- | --- |
|  |  |  |  | **Mean-x** | **CV-x%** | **Mean-x** | **CV-x%** |  |  |  |  |
| CH02 | *R. maddenii* ssp. *maddenii* (as *R. brachysiphon*) | *R. maddenii* (as *R. brachysiphon*) | 6*x* | 51.08 | 5.07 | 149.21 | 2.88 | *R. fortunei* | fresh |  | MPN52752; MPN52753 |
| CH03 | *R. maddenii* ssp. *crassum* | *R. maddenii* | 8*x* | 51.16 | 6.03 | 204.03 | 2.93 | *R. fortunei* | fresh |  | MPN52754; MPN52755; MPN52756; MPN52757 |
| CH04 | *R. veitchianum* (as *R. cubittii*) | *R. cubittii* | 2*x* | 54.96 | 8.17 | n/a | n/a | *R. fortunei* | fresh |  | MPN52758 |
| CH05 | *R. carneum* | *R. carneum* | 2*x* | 55.31 | 8 | n/a | n/a | *R. fortunei* | fresh |  | MPN52759; MPN52760 |
| CH06 | *R. megacalyx* | *R. megacalyx* | 2*x* | 50.19 | 8.87 | n/a | n/a | *R. fortunei* | fresh |  | MPN52761; MPN52762 |
| CH07 | *R. dendricola* | *R. dendricola* | 2*x* | 56.68 | 8.44 | n/a | n/a | *R. fortunei* | fresh |  | MPN52763; MPN53021 |
| CH08 | *R. formosum* | *R. assamicum* | 2*x* | 53.18 | 6.48 | n/a | n/a | *R. fortunei* | fresh |  | MPN52764 |
| CH09 | *R. maddenii* ssp. *crassum* (as *R. manipurense*) | *R. crassum* | 8*x* | 48.23 | 6.85 | 190.77 | 3.2 | *R. fortunei* | fresh |  | MPN52765 |
| CH10 | *R. formosum* var. *formosum* (as *R. formosum*) | *R. formosum* | 2*x* | 50.28 | 5.94 | n/a | n/a | *R. fortunei* | fresh |  | MPN52766; MPN53014 |
| CH11 | *R. lindleyi* | *R. lindleyi* | 2*x* | 51.98 | 7.82 | n/a | n/a | *R. fortunei* | fresh |  | MPN52767 |
| CH14 | *R. veitchianum* (as *R. cubittii*) | *R. ciliicalyx* | 2*x* | 52.94 | 9.69 | n/a | n/a | *R. fortunei* | fresh |  | MPN52770; MPN52771 |
| DN01 | *R. maddenii* ssp. *crassum* | *R. maddenii* ssp. *crassum* | 5-6*x*? | 51.37 | 5.31 | 129.1 | 9.11 | *R. fortunei* | herbarium |  | MPN52952 |
| DN02 | *R. formosum* var. *formosum* (as *R. formosum*) | *R. formosum* | 2*x* | 55.54 | 6.7 | n/a | n/a | *R. fortunei* | herbarium |  | MPN52953 |
| DN03 | *R. valentinianum* var.*oblongilobatum* | *R. valentinianum* var. *oblongilobatum* | 2*x* | 47.33 | 11.47 | n/a | n/a | *R. fortunei* | herbarium |  | MPN52954 |
| DN05 | *R. formosum* var. *inaequale* (as *R. inaequale*) | *R. formosum* var. *formosum* | 2*x* | 46.78 | 9.79 | n/a | n/a | *R. fortunei* | fresh |  | MPN52956 |
| DN06 | *R. formosum* | *R. formosum* var. *inaequale* | 2*x* | 52.01 | 8.06 | n/a | n/a | *R. fortunei* | herbarium |  | MPN52957; MPN53007 |
| DN07 | *R. maddenii* ssp. *crassum* (as *R. crassum*) | *R. maddenii* (*R. polyandrum*) | 5*x*? | 45.87 | 10.53 | 106.08 | 7.26 | *R. fortunei* | herbarium |  | MPN52958; MPN52959; MPN52999 |
| DN08 | *R. maddenii* ssp. *maddenii* | *R. maddenii* (*R. polyandrum*) | 6-7*x*? | 50.58 | 7.16 | 167.81 | 7.14 | *R. fortunei* | herbarium |  | MPN52960 |
| DN09 | *R. nuttallii* | *R. nuttallii* | 2*x* | 39.62 | 8.32 | n/a | n/a | *R. fortunei* | herbarium |  | MPN52961 |
| DN10 | *R. nuttallii* | *R. nuttallii* | 2*x* | 45.79 | 12.51 | n/a | n/a | *R. fortunei* | herbarium |  | MPN52962 |
| DN11 | *R. excellens* | *R. excellens* | 2*x* | 42.9 | 11.12 | n/a | n/a | *R. fortunei* | herbarium |  | MPN52963 |
| DN12 | *R. megacalyx* | *R. megacalyx* | 2*x* | 42.08 | 9.48 | n/a | n/a | *R. fortunei* | herbarium |  | MPN52964 |
| DN14 | *R. horlickianum* | *R. horlickianum* | 2*x* | 44.52 | 10.46 | n/a | n/a | *R. fortunei* | herbarium |  | MPN52966 |
| DN15 | *R. fletcherianum* | *R. fletcherianum* | 2*x* | 45.69 | 11.78 | n/a | n/a | *R. fortunei* | herbarium |  | MPN52967 |
| DN16 | *R. maddenii* ssp. *maddenii* (as *R. maddenii*) | *R. maddenii* Kotuku | 2*x* | 44.6 | 10.07 | n/a | n/a | *R. fortunei* | herbarium |  | MPN52968 |
| DN17 | *R. johnstoneanum* | *R. johnstoneanum* | 2*x* | 47.3 | 8.79 | n/a | n/a | *R. fortunei* | fresh |  | MPN52969 |
| DN18 | *R. horlickianum* | *R. horlickianum* | 2*x* | 47.02 | 11.88 | n/a | n/a | *R. fortunei* | fresh |  | accession died |
| DN20 | *R. pachypodum* | *R. pachypodum* AC 1993 | 2*x* | 44.33 | 11.42 | n/a | n/a | *R. fortunei* | fresh | wild | MPN52970 |
| DN21 | *R. leptocladon* | *R. leptocladon* (H.P. Cox) ex Murdoch | 2*x* | 46.51 | 10.58 | n/a | n/a | *R. fortunei* | herbarium | wild | MPN52971 |
| DN22 | *R. taggianum* | *R. taggianum* CCHH 108 ex Murdoch | 2*x* | 44.77 | 9.65 | n/a | n/a | *R. fortunei* | fresh | wild | MPN52972 |
| E01 | *R. ciliatum* | *R. ciliatum* | 2*x* | 43.48 | 10.67 | n/a | n/a | *R. fortunei* | silica gel-dried | wild: India: Sikkim, N | RBGE#19820583*F |
| E02 | *R. ciliatum* | *R. ciliatum* | 2*x* | 44.97 | 10.86 | n/a | n/a | *R. fortunei* | silica gel-dried | wild: Nepal | RBGE#19850525*K |
| E03 | *R. dalhousiae* var. *dalhousiae* | *R. dalhousiae* | 2*x* | 45.24 | 11.83 | n/a | n/a | *R. fortunei* | silica gel-dried | wild: India: W Bengal | RBGE#19722633*F |
| E04 | *R. dalhousiae* var. *dalhousiae* | *R. dalhousiae* | 2*x* | 45.42 | 12.23 | n/a | n/a | *R. fortunei* | silica gel-dried | wild: India: Sikkim | RBGE#19880631*F |
| E05 | *R. dendricola* | *R. dendricola* | 2*x* | 48.36 | 11.63 | n/a | n/a | *R. fortunei* | silica gel-dried | wild: Myanmar: Kachin State | RBGE#19754089*F |
| E06 | *R. formosum* | *R. formosum* | 2*x* | 49.2 | 12.41 | n/a | n/a | *R. fortunei* | silica gel-dried | wild: India: Meghalaya | RBGE#19751328*G |
| E07 | *R. liliiflorum* | *R. liliiflorum* | 2*x* | 48 | 10.09 | n/a | n/a | *R. fortunei* | silica gel-dried | wild: China: Guizhou | RBGE#19911969*B |
| E08 | *R. liliiflorum* | *R. liliiflorum* | 2*x* | 48.61 | 13.55 | n/a | n/a | *R. fortunei* | silica gel-dried | wild: China: Guizhou | RBGE#19861177*A |
| E09 | *R. ludwigianum* | *R. ludwigianum* | 2*x* | 45.74 | 12.91 | n/a | n/a | *R. fortunei* | silica gel-dried | wild: Thailand: Chiang Mai | RBGE#19850478*D |
| E10 | *R. maddenii* ssp. *crassum* | *R. maddenii* ssp. *crassum* | 2*x* | 51.41 | 8.83 | n/a | n/a | *R. fortunei* | silica gel-dried | wild: China: Yunnan | RBGE#19391033*N |
| E11 | *R. maddenii* ssp. *crassum* | *R. maddenii* ssp. *crassum* | 8*x* | 48.61 | 7.37 | 186.9 | 5.09 | *R. fortunei* | silica gel-dried | wild: India: Arunachal Pradesh | RBGE#19832535*I |
| E12 | *R. maddenii* ssp. *maddenii* | *R. maddenii* ssp. *maddenii* | 8*x* | 51.28 | 10.35 | 200.65 | 5.02 | *R. fortunei* | silica gel-dried | wild: Bhutan: Thimphu Dist. | RBGE#19150028*L |
| E13 | *R. maddenii* ssp. *maddenii* | *R. maddenii* ssp. *maddenii* | 6*x* | 45.87 | 10.2 | 131.31 | 5.14 | *R. fortunei* | silica gel-dried | wild: Bhutan: Tashigang Dist. | RBGE#19754068*I |
| E14 | *R. maddenii* ssp. *maddenii* | *R. maddenii* ssp. *maddenii* | 4-6*x*? | 47.6 | 9.69 | 122.64 | 7.48 | *R. fortunei* | silica gel-dried | wild: China: Xizang (Tibet) Aut. Reg. | RBGE#19141003*C |
| E15 | *R. nuttallii* | *R. nuttallii* | 2*x* | 49.04 | 10.01 | n/a | n/a | *R. fortunei* | silica gel-dried | wild: China: Yunnan - Gaoligong Shan | RBGE#20042030*E |
| E16 | *R. scopulorum* | *R. scopulorum* | 2*x* | 53.15 | 10.61 | n/a | n/a | *R. fortunei* | silica gel-dried | wild: China: Xizang (Tibet) Aut. Reg. | RBGE#19730921*P |
| E17 | *R. veitchianum* | *R. veitchianum* | 2*x* | 55.37 | 11.33 | n/a | n/a | *R. fortunei* | silica gel-dried | wild: Thailand: Chiang Mai | RBGE#19750211*H |
| E18 | *R. fletcherianum* | *R. fletcherianum* | 2*x* | 51.96 | 8.21 | n/a | n/a | *R. fortunei* | silica gel-dried | wild: Xizang (Tibet) Aut. Reg. | RBGE#19754070*J |
| E19 | *R. ciliipes* | *R. ciliipes* | 2*x* | 54.99 | 7.22 | n/a | n/a | *R. fortunei* | silica gel-dried | wild: China: Yunnan | RBGE#19991313*B |
| E20 | *R. maddenii* ssp. *crassum* | *R. maddenii* ssp. *crassum* | 8*x* | 54.88 | 9.12 | 208.28 | 4.37 | *R. fortunei* | silica gel-dried | wild: China: Xizang (Tibet) Aut. Reg. - Yunnan border | RBGE#19491022*B |
| E21 | *R. maddenii* | *R. maddenii* | 6*x* | 52.42 | 8.36 | 141.88 | 5.46 | *R. fortunei* | silica gel-dried | wild: Bhutan: Thimphu Dist. | RBGE#19831844*B |
| E22 | *R. maddenii* | *R. maddenii* | 6*x* | 54.1 | 6.46 | 142.24 | 5.48 | *R. fortunei* | silica gel-dried | wild: India: Sikkim | RBGE#19890430*B |
| E23 | *R. maddenii* ssp. *crassum* | *R. maddenii* ssp. *crassum* | 2*x* | 53.07 | 8.79 | n/a | n/a | *R. fortunei* | silica gel-dried | wild: China: Yunnan | RBGE#19321024*D |
| E24 | *R. maddenii* ssp. *maddenii* | *R. maddenii* ssp. *maddenii* | 6*x* | 53.14 | 7.56 | 154.4 | 5.5 | *R. fortunei* | silica gel-dried | wild: China: Xizang (Tibet) Aut. Reg. | RBGE#19715035*G |
| E25 | *R. maddenii* ssp. *maddenii* | *R. maddenii* ssp. *maddenii* | 6*x* | 48.63 | 10.04 | 128.7 | 3.46 | *R. fortunei* | silica gel-dried | wild: India: Arunachal Pradesh | RBGE#19730904*E |
| HP01 | *R. excellens* | *R. excellens* DT307/98 | 2*x* | 50.59 | 6.84 | n/a | n/a | *R. fortunei* | fresh | wild | MPN52703; MPN52704 |
| HP02 | *R. maddenii* ssp. *maddenii* (as *R. maddenii*) | *R. maddenii* (as *R. polyandrum*) | 6*x* | 54.09 | 5.33 | 156.12 | 3.05 | *R. fortunei* | fresh |  | MPN52705; MPN52706; MPN52707 |
| HP03 | *R. taggianum* | *R. taggianum* | 2*x* | 58.91 | 6.89 | n/a | n/a | *R. fortunei* | fresh |  | MPN52708; MPN52709 |
| HP05 | *R. maddenii* ssp. *maddenii* (as *R. maddenii*) | *R. maddenii* | 6*x* | 53.15 | 6 | 151.36 | 2.76 | *R. fortunei* | fresh | wild: Burma road, Yunnan, China | MPN52712; MPN52713; MPN52714 |
| HP06 | *R. veitchianum* (as *R. cubittii*) | *R. veitchianum*? (*as R. cubittii*) | 2*x* | 47.26 | 9.15 | n/a | n/a | *R. fortunei* | fresh |  | MPN52715 |
| OM45 | *R. veitchianum* (as *R. cubittii*) | *R. veitchianum*? (*as R. cubittii*) | 2*x* | 70.09 | 5.75 | n/a | n/a | *R. fortunei* | fresh |  | MPN52661; MPN52662 |
| HP07 | *R. dalhousiae* var.*rhabdotum* | *R. dalhousiae* var. *rhabdotum* | 2*x* | 45.24 | 6.42 | n/a | n/a | *R. fortunei* | fresh |  | MPN52716; MPN52717; MPN52718 |
| HP08 | *R. maddenii* ssp. *maddenii* (as *R. maddenii*) | *R. maddenii* No. 1, ex Cullinane | 8*x* | 42.97 | 6.24 | 169.24 | 2.81 | *R. fortunei* | fresh |  | MPN52719; MPN52720 |
| HP09 | *R. maddenii* ssp. *crassum* (as *R. crassum*) | *R. maddenii* ssp. *crassum* DT 98 | 8*x* | 42.73 | 5.43 | 170.83 | 3.48 | *R. fortunei* | fresh | wild | MPN52721; MPN52722 |
| HP10 | *R. maddenii* ssp. *maddenii* (as *R. maddenii*) | *R. maddenii* ssp. *crassum* Jury selection | 6*x* | 50.97 | 5.15 | 148.24 | 2.93 | *R. fortunei* | fresh |  | MPN52723; MPN52724; MPN52725 |
| HP11 | *R. maddenii* ssp. *maddenii* (as *R. maddenii*) | *R. maddenii* ssp. *crassum* Jury selection (as *R. polyandrum*)? | 6*x* | 51.07 | 4.64 | 143.23 | 3.42 | *R. fortunei* | fresh |  | MPN52726; MPN52727 |
| HP13 | *R. ciliipes* | *R. ciliipes* | 2*x* | 54.61 | 9.42 | n/a | n/a | *R. fortunei* | fresh |  | MPN52730; MPN52731; MPN52732; MPN52733 |
| HP14 | *R. lyi* | *R. ciliicalyx* ssp. *lyi* Gigha form (as *R. lyi*) | 2*x* | 56.11 | 6.54 | n/a | n/a | *R. fortunei* | fresh |  | MPN52734; MPN52735 |
| HP15 | *R. maddenii* ssp. *crassum* (as *R. crassum*) | *R. maddenii* ssp. *maddenii* | 8*x* | 51.25 | 6.69 | 197.97 | 3.62 | *R. fortunei* | fresh |  | MPN52736; MPN52737 |
| HP16 | *R. pachypodum* (as *R. supranubium*) | *R. pachypodum* (as *R. supranubium*) | 2*x* | 52.89 | 8.84 | n/a | n/a | *R. fortunei* | fresh |  | MPN52738; MPN52739 |
| HP17 | *R. taggianum* | *R. taggianum* | 2*x* | 56.5 | 7.5 | n/a | n/a | *R. fortunei* | fresh |  | MPN52740; MPN52741 |
| HP19 | *R. taggianum* | *R. taggianum* | 2*x* | 53.68 | 11.68 | n/a | n/a | *R. fortunei* | silica gel-dried |  | MPN52743; MPN52744; MPN53025 |
| HP20 | *R. maddenii* ssp. *maddenii* (as *R. maddenii*) | *R. polyandrum* | 6*x* | 50.25 | 7.02 | 143.21 | 3.72 | *R. fortunei* | fresh |  | MPN52745; MPN52998 |
| HP21 | *R.* aff. *valentinianum* | *R. valentinianum* aff. | 2*x* | 57.2 | 8.31 | n/a | n/a | *R. fortunei* | fresh |  | MPN52746; MPN53022 |
| OM01 | *R. formosum* var. *formosum* (as *R. formosum*) | *R. inaequale* | 2*x* | 67.55 | 5.04 | n/a | n/a | *R. fortunei* | fresh |  | MPN52583; MPN52584 |
| HP18 | *R. formosum* var. *formosum* (as *R. formosum*) | *R. inaequale* | 2*x* | 54.39 | 7.2 | n/a | n/a | *R. fortunei* | fresh |  | MPN52742 |
| OM02* | *R. maddenii* ssp. *maddenii* (as *R. maddenii*) | *R. maddenii* (as *R.* *manipurense*) | 6*x* | 63.14 | 6.78 | 178.28 | 2.77 | *R. fortunei* | fresh |  | MPN52585; MPN52586 |
| OM03 | *R. ciliatum* | *R. ciliatum* | 2*x* | 65.45 | 6.69 | n/a | n/a | *R. fortunei* | fresh |  | MPN52587; MPN52588 |
| OM04 | *R. pachypodum* | *R. pachypodum* (as *R. supranubium*) | 2*x* | 67.71 | 10.75 | n/a | n/a | *R. fortunei* | fresh |  | MPN52589; MPN52590; MPN52591 |
| HP04 | *R. pachypodum* | *R. pachypodum* (as *R. supranubium*) | 2*x* | 57.68 | 6.35 | n/a | n/a | *R. fortunei* | fresh |  | MPN52710; MPN52711; MPN53015 |
| OM05 | *R. burmanicum* | *R. burmanicum* | 2*x* | 61.06 | 6.95 | n/a | n/a | *R. fortunei* | fresh |  | MPN52592; MPN52592 |
| OM06* | *R. ciliicalyx* | *R. ciliicalyx* | 2*x* | 66.52 | 6.88 | n/a | n/a | *R. fortunei* | fresh |  | MPN52594; MPN52595 |
| OM07 | *R. ciliicalyx* | *R. ciliicalyx* ‘Charisma’ | 2*x* | 61.99 | 6.78 | n/a | n/a | *R. fortunei* | fresh |  | MPN52596; MPN52597 |
| OM08 | *R. ciliatum* | *R. ciliatum* | 2*x* | 60.91 | 5.02 | n/a | n/a | *R. fortunei* | fresh |  | MPN52598; MPN52599 |
| OM09 | *R. formosum* var. *formosum* (as *R. formosum*) | *R. formosum* (as *R. assamicum*) | 2*x* | 64.14 | 5.39 | n/a | n/a | *R. fortunei* | fresh |  | MPN52600; MPN52601 |
| OM10 | *R. formosum* var. *formosum* (as *R. iteophyllum*) | *R. formosum* (as *R. iteaphyllum*) | 2*x* | 62.94 | 5.96 | n/a | n/a | *R. fortunei* | fresh |  | MPN52602; MPN52603 |
| OM11* | *R. maddenii* ssp. *maddenii* (as *R. maddenii*) | *R. maddenii* SD1104 | 6*x* | 63.34 | 6.33 | 180.47 | 2.66 | *R. fortunei* | fresh | wild: Sikkim | MPN52604; MPN52605 |
| OM12 | *R. nuttallii* var. *stellatum* | *R. nuttallii* 'stellata' | 2*x* | 58.75 | 5.71 | n/a | n/a | *R. fortunei* | fresh |  | MPN52606; MPN52607 |
| OM13 | *R. johnstoneanum* | *R. johnstoneanum* KB form | 2*x* | 62.09 | 6.81 | n/a | n/a | *R. fortunei* | fresh |  | MPN52608; MPN52609 |
| OM14* | *R. maddenii* ssp. *maddenii* (as *R. maddenii*) | *R. maddenii* TH268? | 8*x* | 62.33 | 6.37 | 250.59 | 2.84 | *R. fortunei* | fresh | wild: Vietnam | MPN52610; MPN52611 |
| OM15 | *R. formosum* var. *formosum* (as *R. formosum*) | *R. veitchianum* (as *R. cubittii*) | 2*x* | 59.87 | 7.99 | n/a | n/a | *R. fortunei* | fresh |  | MPN52612; MPN52613 |
| OM16 | *R. parryae* | *R. parryae* | 2*x* | 59.26 | 7.05 | n/a | n/a | *R. fortunei* | fresh |  | MPN52614; MPN52615 |
| OM17* | *R. maddenii* ssp. *crassum* (as *R. crassum*) | *R. maddenii* ssp. *crassum* | 8*x* | 59.75 | 7.05 | 239.78 | 2.45 | *R. fortunei* | fresh |  | MPN52616; MPN52617; MPN53000 |
| OM18* | *R. maddenii* ssp. *maddenii* (as *R. maddenii*) | *R. maddenii* ‘Late Pink’ | 6*x* | 61.73 | 6.62 | 174.45 | 3.19 | *R. fortunei* | fresh |  | MPN52618; MPN52619 |
| OM19 | *R. johnstoneanum* | *R. johnstoneanum* | 2*x* | 56.03 | 7.16 | n/a | n/a | *R. fortunei* | fresh |  | MPN52620; MPN52621 |
| OM20* | *R. maddenii* ssp. *maddenii* (as *R. maddenii*) | *R. maddenii* | 6*x* | 58.28 | 6.42 | 166.06 | 2.64 | *R. fortunei* | fresh |  | MPN52622; MPN52623 |
| OM22 | *R. lindleyi* | *R. lindleyi* | 2*x* | 61.42 | 5.64 | n/a | n/a | *R. fortunei* | fresh |  | accession died |
| OM23 | *R. lindleyi* | *R. lindleyi* ‘Helen Gordon’ | 2*x* | 60.31 | 5.58 | n/a | n/a | *R. fortunei* | fresh |  | MPN52679; MPN52680 (accession is a clone of OM52) |
| OM52 | *R. lindleyi* | *R. lindleyi* ‘Helen Gordon’ | 2*x* | 79.22 | 4.58 | n/a | n/a | *R. fortunei* | fresh |  | MPN52679; MPN52680 |
| OM24 | *R. lindleyi* | *R. lindleyi* | 2*x* | 59.88 | 5.52 | n/a | n/a | *R. fortunei* | fresh |  | accession died |
| OM25 | *R. veitchianum* | *R. veitchianum* | 2*x* | 59.44 | 6.15 | n/a | n/a | *R. fortunei* | fresh |  | MPN52624; MPN52625 |
| OM26 | *R. dalhousiae* var*. rhabdotum* | *R. dalhousiae* var. *rhabdotum* | 2*x* | 59.3 | 6.39 | n/a | n/a | *R. fortunei* | fresh |  | MPN52626 |
| OM29 | *R. dalhousiae* var.*rhabdotum* | *R. dalhousiae* var. *rhabdotum* | 2*x* | 56.6 | 6.3 | n/a | n/a | *R. fortunei* | fresh |  | MPN52630 |
| OM30 | *R. johnstoneanum* | *R. johnstoneanum* | 2*x* | 55.29 | 6.53 | n/a | n/a | *R. fortunei* | fresh |  | MPN52631; MPN52632 |
| OM32* | *R. dalhousiae* var. *dalhousiae* | *R. dalhousiae* var. *dalhousiae* | 2*x* | 63.83 | 6.58 | n/a | n/a | *R. fortunei* | fresh |  | MPN52634; MPN52635 |
| OM33 | *R. parryae* | *R. parryae* | 2*x* | 59.15 | 5.09 | n/a | n/a | *R. fortunei* | fresh |  | MPN52636; MPN52637 |
| OM34* | *R. excellens* | *R. excellens* | 2*x* | 62.49 | 6 | n/a | n/a | *R. fortunei* | fresh | wild: Nth Vietnam | MPN52638; MPN52639 |
| OM36 | *R. burmanicum* | *R. burmanicum* | 2*x* | 56.1 | 4.93 | n/a | n/a | *R. fortunei* | fresh |  | MPN52643; MPN52644 |
| OM37 | *R. johnstoneanum* | *R. johnstoneanum* KB form | 2*x* | 59.89 | 5.4 | n/a | n/a | *R. fortunei* | fresh |  | MPN52645; MPN52646 |
| OM38 | *R. burmanicum* | *R. burmanicum* Marchant form | 2*x* | 57.78 | 5.9 | n/a | n/a | *R. fortunei* | fresh |  | MPN52647; MPN52648 |
| OM39 | *R. scopulorum* | *R. scopulorum* | 2*x* | 64.34 | 4.91 | n/a | n/a | *R. fortunei* | fresh |  | MPN52649; MPN52650 |
| OM40* | *R. taronense* | *R. taronense* | 2*x* | 63.58 | 6.14 | n/a | n/a | *R. fortunei* | fresh |  | MPN52651; MPN52652 |
| OM41 | *R. carneum* | *R. carneum* | 2*x* | 65.51 | 8.44 | n/a | n/a | *R. fortunei* | fresh |  | MPN52653; MPN52654 |
| OM42 | *R. formosum* var. *formosum* (as *R. formosum*) | *R. veitchianum* (as *R. cubittii*) | 2*x* | 70.64 | 7.02 | n/a | n/a | *R. fortunei* | fresh |  | MPN52655; MPN52656 |
| OM43* | *R. formosum* var. *formosum* (as *R. formosum*) | *R. formosum* | 2*x* | 62.36 | 6.61 | n/a | n/a | *R. fortunei* | fresh |  | MPN52657; MPN52658 |
| OM44 | *R. formosum* | *R. aff. veitchianum* KR2247 | 2*x* | 64.47 | 5.27 | n/a | n/a | *R. fortunei* | fresh | wild | MPN52659; MPN52660 |
| OM46* | *R. maddenii* ssp. *maddenii* (as *R. maddenii*) | *R. maddenii* ‘Jury Selection’ (*R. polyandrum)* | 6*x* | 59.02 | 4.98 | 165.86 | 2.9 | *R. fortunei* | fresh |  | MPN52663; MPN52664 |
| OM47* | *R. maddenii* ssp. *crassum* (as *R. manipurense*) | *R. maddenii* ssp. *crassum* (as *R. manipurense*) | 8*x* | 57.87 | 6.34 | 228.08 | 2.63 | *R. fortunei* | fresh |  | MPN52665; MPN52666 |
| OM48* | *R. maddenii* ssp. *maddenii* (as *R. maddenii*) | *R. maddenii* ssp. *crassum* (as *R*. *manipurense*) | 7*x* | 57.4 | 6.48 | 197.92 | 3.84 | *R. fortunei* | fresh | wild: possibly Nth Vietnam | MPN52667; MPN52668; MPN52669 |
| OM49* | *R. maddenii* ssp. *maddenii* (as *R. maddenii*) | *R. maddenii* | 6*x* | 58.46 | 5.54 | 179.98 | 2.86 | *R. fortunei* | fresh |  | MPN52670; MPN52671 |
| OM50 | *R. nuttallii* | *R. nuttallii* | 2*x* | 71.09 | 7.08 | n/a | n/a | *R. fortunei* | fresh | wild: Nth Vietnam | MPN52672; MPN52673; MPN52674; MPN52675 |
| OM51 | *R. nuttallii* | *R. nuttallii* | 2*x* | 71.66 | 10.14 | n/a | n/a | *R. fortunei* | fresh | wild: China | MPN52676; MPN52677; MPN52678; MPN53018 |
| OM53 | *R. sinonuttallii* | *R. sinonuttallii* | 2*x* | 71.29 | 4.98 | n/a | n/a | *R. fortunei* | fresh |  | MPN52681; MPN52682; MPN52683 |
| OM54* | *R. maddenii* ssp. *maddenii* (as *R. maddenii*) | *R. maddenii ‘*Virginalis’ | 6*x* | 58.98 | 5.97 | 170.11 | 2.87 | *R. fortunei* | fresh |  | MPN52684; MPN52685 |
| OM55* | *R. burmanicum* | *R. burmanicum* | 2*x* | 57.16 | 4.7 | n/a | n/a | *R. fortunei* | fresh |  | MPN52686 |
| OM56* | *R. maddenii* ssp. *maddenii* (as *R. maddenii*) | *R. crassum* | 6*x* | 58.53 | 6.9 | 167.38 | 3.5 | *R. fortunei* | fresh |  | MPN52687 |
| OM57* | *R. sinonuttallii* | *R. sinonuttallii* | 2*x* | 57.44 | 6.64 | n/a | n/a | *R. fortunei* | fresh |  | MPN52688 |
| OM58* | *R. maddenii* ssp. *maddenii* (as *R. brachysiphon*) | *R. brachysiphon* | 6*x* | 54.99 | 5.97 | 163.53 | 3.42 | *R. fortunei* | fresh |  | MPN52689 |
| PK01 | *R. excellens* | *R. excellens* KR2998 | 2*x* | 50.58 | 5.7 | n/a | n/a | *R. parryae* | fresh | wild | MPN52774; MPN52775 |
| PK02 | *R. horlickianum* | *R. horlickianum* Gwavas | 2*x* | 52.72 | 4.49 | n/a | n/a | *R. parryae* | fresh |  | MPN52776 |
| PK03 | *R. ciliipes* | *R. ciliipes* K56 | 2*x* | 52.18 | 4.51 | n/a | n/a | *R. parryae* | fresh | wild | MPN52777; MPN52778; MPN52779; MPN52780 |
| PK04 | *R. formosum* var. *formosum* (as *R. iteophyllum*) | *R. iteophyllum* Gigha form | 2*x* | 52.12 | 4.62 | n/a | n/a | *R. parryae* | fresh |  | MPN52781; MPN52782; MPN52783; MPN52784 |
| PK05# | *R. taggianum* | *R. taggianum* | 2*x* | 54.11 | 7.93 | n/a | n/a | *R. parryae* | fresh |  | accession died |
| PK06 | *R. excellens* | *R. excellens* AC435 | 2*x* | 49.56 | 6.48 | n/a | n/a | *R. parryae* | fresh | wild | MPN52785; MPN52786 |
| PK07 | *R. pachypodum* (as *R. supranubium*) | *R. pachypodum* (as *R. supranubium*) | 2*x* | 50.77 | 5.69 | n/a | n/a | *R. parryae* | fresh |  | MPN52787; MPN52788; MPN52789; MPN52790 |
| PK08 | *R. formosum* var. *formosum* (as *R. iteophyllum*) | *R. iteophyllum* Brodick (*R. formosum* var. *formosum* on the tag) | 2*x* | 50.91 | 5.25 | n/a | n/a | *R. parryae* | fresh |  | MPN52791; MPN52792; MPN52793 |
| PK09# | *R. maddenii* ssp. *maddenii* (as *R. maddenii*) | *R. maddenii* ssp. *maddenii* (as *R. brachysiphon*) | 7*x* | 61.12 | 5.09 | 163.18 | 2.74 | *R. fortunei* | fresh |  | MPN52794; MPN52795; MPN52796; MPN52797 |
| PK10 | *R. excellens* | *R. excellens* TVO 064 | 2*x* | 58.56 | 5.27 | n/a | n/a | *R. parryae* | fresh | wild | MPN52798; MPN52799; MPN52800 |
| PK11 | *R. veitchianum* (as *R. cubittii*) | *R. formosum* var. *inaequale* KW16029 | 2*x* | 75.65 | 4.21 | n/a | n/a | *R. parryae* | fresh | wild | MPN52801; MPN52802; MPN52803 |
| PK12 | *R. scopulorum* | *R. scopulorum* | 2*x* | 72.55 | 5.05 | n/a | n/a | *R. parryae* | fresh |  | MPN52804; MPN52805; MPN52806; MPN52807; MPN52808 |
| PK13 | *R. maddenii* ssp. *maddenii* | *R. maddenia* ssp. *maddenii* (as *R. polyandrum*) | 5*x* | 72.22 | 4.05 | 232.19 | 2.33 | *R. fortunei* | fresh |  | MPN52809; MPN52810 |
| PK14 | *R. parryae* | *R. parryae* RBGE146 Fischer form | 2*x* | 74.35 | 4.39 | n/a | n/a | *R. parryae* | fresh | wild | MPN52811; MPN52812 |
| PK15 | *R. burmanicum* | *R. burmanicum* Brodick form | 2*x* | 74.48 | 4.61 | n/a | n/a | *R. parryae* | fresh |  | MPN52813; MPN52814 |
| PK16 | *R. valentinianum* var.*oblongilobatum* | *R. valentinianum* var. *oblongilobatum* AC1258? | 2*x* | 58.74 | 5.67 | n/a | n/a | *R. parryae* | fresh | wild | MPN52815; MPN52816; MPN52817 |
| PK17# | *R. maddenii* ssp. *maddenii* (as *R. maddenii*) | *R. maddenii* TH. 237 | 8*x* | 59.74 | 5.13 | 216.78 | 2.24 | *R. fortunei* | fresh | wild | MPN52818; MPN52819; MPN52820 |
| PK18 | *R. formosum* var. *formosum* (as *R. formosum*) | *R. formosum* Ten Tashi | 2*x* | 58.52 | 4.93 | n/a | n/a | *R. parryae* | fresh | wild | MPN52821; MPN52822; MPN52823 |
| PK19 | *R. dendricola* | *R. dendricola* | 2*x* | 55.41 | 5.46 | n/a | n/a | *R. parryae* | fresh |  | MPN52824; MPN52825; MPN52826; MPN52827; MPN52828 |
| PK20 | *R. burmanicum* | *R. burmanicum '*Deep Yellow' | 2*x* | 54.3 | 6.13 | n/a | n/a | *R. parryae* | fresh |  | MPN52829; MPN52830; MPN52831 |
| PK21 | *R. veitchianum* | *R. veitchianum* 'Ice White' | 2*x* | 58.45 | 5.1 | n/a | n/a | *R. parryae* | fresh | wild: Thailand | MPN52832; MPN52833; MPN52834 |
| PK22# | *R. maddenii* ssp. *crassum* (as *R. manipurense*) | *R. maddenii* ssp. *maddenii* (as *R. polyandrum*) | 8*x* | 56.84 | 5.26 | 204.51 | 2.81 | *R. fortunei* | fresh |  | MPN52836; MPN52837; MPN52838 |
| PK23 | *R. dalhousiae* var.*rhabdotum* | *R. dalhousiae* var. *rhabdotum* | 2*x* | 54.64 | 5.8 | n/a | n/a | *R. parryae* | fresh |  | MPN52839; MPN52840 |
| PK24 | *R. carneum* | *R. carneum* | 2*x* | 58.22 | 5.73 | n/a | n/a | *R. parryae* | fresh |  | MPN52841; MPN52842; MPN52843 |
| PK25A | *R. dendricola* | *R. dendricola* | 2*x* | 56.41 | 6.39 | n/a | n/a | *R. parryae* | fresh |  | MPN52844; MPN52845; MPN52846 |
| PK26 | *R. pachypodum* (as *R. supranubium*) | *R. pachypodum* | 2*x* | 56.29 | 4.85 | n/a | n/a | *R. parryae* | fresh |  | MPN52847; MPN52848; MPN52849; MPN52850 |
| PK27# | *R. maddenii* ssp. *maddenii* (as *R. maddenii*) | *R. maddenii* ssp. *crassum* | 6*x* | 56.24 | 6.01 | 146.7 | 2.75 | *R. fortunei* | fresh |  | MPN52851; MPN52852; MPN52853 |
| PK28 | *R. lindleyi* | *R. lindleyi* KW8546 ex Gwavas | 2*x* | 55.16 | 6.57 | n/a | n/a | *R. parryae* | fresh | wild | MPN52854; MPN52855 |
| PK29 | *R. coxianum* | *R. coxianum* 1984-443, Glendoick? | 2*x* | 69.92 | 5.2 | n/a | n/a | *R. parryae* | fresh | wild | MPN52856; MPN52857 |
| PK30 | *R. excellens* | *R. excellens* AC5630 | 2*x* | 72.97 | 4.49 | n/a | n/a | *R. parryae* | fresh | wild: Ban Khoang, Vietnam | MPN52860; MPN52861; MPN52862 |
| PK31 | *R. megacalyx* | *R. megacalyx* | 2*x* | 54.33 | 6.9 | n/a | n/a | *R. parryae* | fresh |  | MPN52863; MPN52864; MPN52865; MPN52866 |
| PK32 | *R. nuttallii* | *R. nuttallii* | 2*x* | 55.08 | 5.6 | n/a | n/a | *R. parryae* | fresh |  | MPN52867; MPN52868; MPN52869; MPN52870 |
| PK33 | *R. johnstoneanum* | *R. johnstoneanum* KW20305 | 2*x* | 64.84 | 6.53 | n/a | n/a | *R. parryae* | fresh | wild | MPN52871; MPN52872 |
| PK34 | *R. dalhousiae* var. *dalhousiae* | *R. dalhousiae* | 2*x* | 72.38 | 4.76 | n/a | n/a | *R. parryae* | fresh | wild: Solu Khumbu, Nepal | MPN52873; MPN52874 |
| PK35 | *R. nuttallii* var. *stellatum* | *R. nuttallii* 'Stellata' | 2*x* | 70.17 | 5.3 | n/a | n/a | *R. parryae* | fresh |  | MPN52875; MPN52876; MPN52877; MPN53024 |
| PK36 | *R. formosum* var. *formosum* (as *R. formosum*) | *R. veitchianum* 'Ashcombe' | 2*x* | 70.74 | 5.04 | n/a | n/a | *R. parryae* | fresh |  | MPN52878; MPN52879 |
| PK37 | *R. dalhousiae* var. *dalhousiae* | *R. dalhousiae* | 2*x* | 72.98 | 4.38 | n/a | n/a | *R. parryae* | fresh |  | MPN52880; MPN52881 |
| PK38# | *R. maddenii* ssp. *maddenii* (as *R. polyandrum*) | *R. maddenii* NAPE 109 | 6*x* | 69.55 | 5.22 | 178.2 | 3.52 | *R. fortunei* | fresh | wild: Nagaland-Araunchal Pradesh | MPN52882; MPN52883 |
| PK39 | *R. lindleyi* | *R. lindleyi* Hollard form | 2*x* | 70.39 | 5.22 | n/a | n/a | *R. parryae* | fresh |  | MPN52884; MPN52885 |
| PK40 | *R. ciliatum* | *R. ciliatum* | 2*x* | 67.11 | 4.75 | n/a | n/a | *R. parryae* | fresh |  | MPN52886; MPN52887; MPN52888; MPN52889 |
| PK41 | *R. burmanicum* | *R. burmanicum* | 2*x* | 68.67 | 4.86 | n/a | n/a | *R. parryae* | fresh |  | MPN52890; MPN52891 |
| PK42 | *R. taronense* | *R. taronense* | 2*x* | 69.37 | 4.58 | n/a | n/a | *R. parryae* | fresh |  | MPN52892; MPN52893; MPN52894 |
| PK43 | *R. ludwigianum* | *R. ludwigianum* Doi Ang Kang, Thailand | 2*x* | 69.28 | 4.36 | n/a | n/a | *R. parryae* | fresh | wild | MPN52895; MPN52896; MPN52897 |
| PK44 | *R. lyi* | *R. lyi* | 2*x* | 69.81 | 5.16 | n/a | n/a | *R. parryae* | fresh |  | MPN52898; MPN52899 |
| PK45# | *R. maddenii* ssp. *crassum* (as *R. crassum*) | *R. maddenii* ssp. *crassum* | 7*x* | 67.11 | 4.97 | 212.12 | 2.47 | *R. fortunei* | fresh |  | MPN52900; MPN52901; MPN52902; MPN53001 |
| PK47 | *R. johnstoneanum* | *R. johnstoneanum* AC5532 | 2*x* | 71.3 | 4.87 | n/a | n/a | *R. parryae* | fresh | wild | MPN52906; MPN52907 |
| PK49 | *R. veitchianum* (as *R. cubittii*) | *R. veitchianum* | 2*x* | 63.18 | 5.54 | n/a | n/a | *R. parryae* | fresh |  | MPN52911 |
| PK50 | *R. ciliatum* | *R. ciliatum* | 2*x* | 64.04 | 4.86 | n/a | n/a | *R. parryae* | fresh |  | MPN52912; MPN52913; MPN52914 |
| PK51 | *R. ciliatum* | *R. ciliatum* | 2*x* | 66.76 | 4.78 | n/a | n/a | *R. parryae* | fresh |  | MPN52915; MPN52916 |
| PK52# | *R. maddenii* ssp. *maddenii* (as *R. polyandrum*) | *R. maddenii* KC0108 | 6*x* | 68.34 | 4.53 | 181.49 | 2.13 | *R. fortunei* | fresh | wild: Araunchal Pradesh | MPN52917; MPN52918; MPN52919; MPN52920; MPN52921 |
| PK53 | *R. ciliicalyx* | *R. ciliicalyx* 'Charisma' KW20280 | 2*x* | 67.43 | 4.4 | n/a | n/a | *R. parryae* | fresh | wild | MPN52922; MPN52923 |
| PK56 | *R. dendricola* | *R. dendricola* | 2*x* | 64.77 | 5.28 | n/a | n/a | *R. parryae* | fresh |  | MPN52928; MPN52929 |
| PK57 | *R. taronense* | *R. taronense* | 2*x* | 65.14 | 5.02 | n/a | n/a | *R. parryae* | fresh |  | MPN52930; MPN52931 |
| PK58 | *R. veitchianum* | *R. veitchianum* OS Blumhardt collection | 2*x* | 62.66 | 5.45 | n/a | n/a | *R. parryae* | fresh | wild: Thailand | MPN52932; MPN52933 |
| PK59# | *R. maddenii* ssp. *crassum* (as *R. manipurense*) | *R. maddenii* ssp. *crassum* Nape 025 | 6*x* | 66.14 | 4.43 | 171.57 | 2.52 | *R. fortunei* | fresh | wild: Aranachal Pradesh | MPN52934; MPN52935 |
| PK60 | *R. nuttallii* | *R. nuttallii* | 2*x* | 63.97 | 4.72 | n/a | n/a | *R. parryae* | fresh | wild: China | MPN52936; MPN52937; MPN52938 |
| PK61# | *R. maddenii* ssp. *crassum* (as *R. crassum*) | *R. maddenii* ssp. *crassum* (*R. odoriferum*) | 7*x* | 63.57 | 5.48 | 197.26 | 2.68 | *R. fortunei* | fresh |  | MPN52939; MPN52940 |
| PK63 | *R. lindleyi* | *R. lindleyi* | 2*x* | 63.42 | 5.47 | n/a | n/a | *R. parryae* | fresh |  | MPN52942 |
| PK66 | *R. ludwigianum* | *R. ludwigianum* | 2*x* | 65.03 | 5.28 | n/a | n/a | *R. parryae* | fresh |  | MPN52948; MPN52949 |
| PK67 | *R. lindleyi* | *R. lindleyi* | 2*x* | 63.03 | 5.68 | n/a | n/a | *R. parryae* | fresh |  | MPN52950 |
| PK68# | *R. maddenii* ssp. *maddenii* (as *R. brachysiphon*) | *R. maddenii ssp. maddenii* (as *R. brachysiphon*) | 6*x* | 60.92 | 5.05 | 158.64 | 3.14 | *R. fortunei* | fresh |  | MPN52951 |
| RSF01 | *R. burmanicum* | *R. burmanicum* | 2*x* | 61.84 | 10.23 | n/a | n/a | *R. fortunei* | silica gel-dried | wild | RSBG#99/095 |
| RSF02 | *R. changii* | *R. changii* JN 11029, wc Nov 2011 | 2*x* | 51.74 | 10.45 | n/a | n/a | *R. fortunei* | silica gel-dried | wild | RSBG#2015/043 |
| RSF03 | *R. ciliatum* | *R. ciliatum* LS&H 16019 | 2*x* | 59.49 | 9.8 | n/a | n/a | *R. fortunei* | silica gel-dried | wild: Bhutan | RSBG#1980/077 |
| RSF04 | *R. ciliatum* | *R. ciliatum* SEH 545 | 2*x* | 63.07 | 9.51 | n/a | n/a | *R. fortunei* | silica gel-dried | wild: Sikkim | RSBG#2000/094 |
| RSF05 | *R. ciliicalyx* | *R. ciliicalyx* TH#1276 2013/113 | 2*x* | 61.25 | 10.19 | n/a | n/a | *R. fortunei* | silica gel-dried | wild | RSBG |
| RSF06 | *R. crenulatum* | *R. crenulatum* NV#072 | 2*x* | 64.31 | 9.84 | n/a | n/a | *R. fortunei* | silica gel-dried | wild | RSBG |
| RSF07 | *R. cuffeanum* | *R. cuffeanum* KW 21909 | 2*x* | 58.33 | 9.39 | n/a | n/a | *R. fortunei* | silica gel-dried | wild | RSBG#2001/148 |
| RSF08 | *R. dalhousiae* var. *dalhousiae* | *R. dalhousiae* var. *dalhousiae* SEH 522 | 2*x* | 60.91 | 11.02 | n/a | n/a | *R. fortunei* | silica gel-dried | wild | RSBG#99/153 |
| RSF09 | *R. dalhousiae* var.*rhabdotum* | *R. dalhousiae* var. *rhabdotum* KCSH 0310 | 2*x* | 56.4 | 9.21 | n/a | n/a | *R. fortunei* | silica gel-dried | wild: India | RSBG#2006/046 |
| RSF10 | *R. dendricola* | *R. dendricola* F 17227 | 2*x* | 66.51 | 10.15 | n/a | n/a | *R. fortunei* | silica gel-dried | wild: Himalaya | RSBG#1998/021 |
| RSF11 | *R. dendricola* | *R. dendricola* SEH 25037 | 2*x* | 60.33 | 11.17 | n/a | n/a | *R. fortunei* | silica gel-dried | wild: China | RSBG#2007/246 |
| RSF12 | *R. dendricola* | *R. dendricola* DGEY#046 2013/490 | 2*x* | 63.31 | 9.07 | n/a | n/a | *R. fortunei* | silica gel-dried | wild | RSBG |
| RSF13 | *R. excellens* | *R. excellens* TH#3773 | 2*x* | 61.12 | 11.07 | n/a | n/a | *R. fortunei* | silica gel-dried | wild | RSBG#2019/054 |
| RSF14 | *R. fletcherianum* | *R. fletcherianum* | 2*x* | 56.78 | 12.88 | n/a | n/a | *R. fortunei* | silica gel-dried | wild | RSBG#65/355 |
| RSF15 | *R. fleuryi* | *R. fleuryi* KR 3286 | 2*x* | 62.99 | 9.21 | n/a | n/a | *R. fortunei* | silica gel-dried | wild: Vietnam | RSBG#1998/009 |
| RSF16 | *R. formosum* | *R. formosum* C&H 320 | 2*x* | 60.62 | 10.92 | n/a | n/a | *R. fortunei* | silica gel-dried | wild: Khasia, India | RSBG#1998/012 |
| RSF17 | *R. formosum* var. *inaequale* | *R. formosum* var. *inaequale* C&H 301 | 2*x* | 59.38 | 11.37 | n/a | n/a | *R. fortunei* | silica gel-dried | wild: India | RSBG#1998/002 |
| RSF18 | *R.* aff. *formosum* | *R. formosum* aff. APA#008 | 2*x* | 59.2 | 10.72 | n/a | n/a | *R. fortunei* | silica gel-dried | wild | RSBG#07/249 |
| RSF19 | *R. goreri* | *R. goreri* KR 6027 | 2*x* | 63.48 | 10.24 | n/a | n/a | *R. fortunei* | silica gel-dried | wild: China | RSBG#2003/188 |
| RSF20 | *R. goreri* | *R. goreri* RR 112 | 2*x* | 63.42 | 9.26 | n/a | n/a | *R. fortunei* | silica gel-dried | wild: China | RSBG#2005/249 |
| RSF21 | *R. horlickianum* | *R. horlickianum* CCHH 8074 | 2*x* | 61.13 | 10.29 | n/a | n/a | *R. fortunei* | silica gel-dried | wild: China | RSBG#2002/011 |
| RSF22 | *R. horlickianum* | *R. horlickianum* KW 9403 | 2*x* | 59.4 | 12.3 | n/a | n/a | *R. fortunei* | silica gel-dried | wild: Burma&China | RSBG#1987/053 |
| RSF23 | *R. johnstoneanum* | *R. johnstoneanum* NAPE 024 | 2*x* | 64.52 | 10.35 | n/a | n/a | *R. fortunei* | silica gel-dried | wild: India&Bhutan | RSBG |
| RSF24 | *R. kiangsiense* | *R. kiangsiense* EN | 2*x* | 69.48 | 9.59 | n/a | n/a | *R. fortunei* | silica gel-dried | wild | RSBG#2013/269 |
| RSF25 | *R. kuomeianum* | *R. kuomeianum* SEH#171 | 2*x* | 55.19 | 12.14 | n/a | n/a | *R. fortunei* | silica gel-dried | wild | RSBG |
| RSF26 | *R. leptocladon* | *R. leptocladon* KR 2932 | 2*x* | 65.64 | 9.45 | n/a | n/a | *R. fortunei* | silica gel-dried | wild: Vietnam | RSBG#1996/066 |
| RSF27 | *R. leptocladon* | *R. leptocladon* HWJ#99688 | 2*x* | 69.75 | 8.54 | n/a | n/a | *R. fortunei* | silica gel-dried | wild | RSBG#2005/035 |
| RSF28 | *R. levinei* | *R. levinei* CGG#14162 | 2*x* | 62.15 | 11.67 | n/a | n/a | *R. fortunei* | silica gel-dried | wild | RSBG#2020/142 |
| RSF29 | *R. levinei* | *R. levinei* | 2*x* | 67.55 | 9.42 | n/a | n/a | *R. fortunei* | silica gel-dried | wild | RSBG#2002/006 |
| RSF30 | *R.* aff. *levinei* | *R. levinei* aff.TH#2805 | 2*x* | 67.52 | 10.33 | n/a | n/a | *R. fortunei* | silica gel-dried | wild | RSBG#2013/244 |
| RSF31 | *R. liliiflorum* | *R. liliiflorum* CGG 14066 | 2*x* | 68.14 | 10.16 | n/a | n/a | *R. fortunei* | silica gel-dried | wild | RSBG#2012/036 |
| RSF32 | *R. liliiflorum* | *R. liliiflorum* PW 116 | 2*x* | 70.59 | 9.57 | n/a | n/a | *R. fortunei* | silica gel-dried | wild: China | RSBG#2005/166 |
| RSF33 | *R. liliiflorum* | *R. liliiflorum* NN#0958 | 2*x* | 64.7 | 8.71 | n/a | n/a | *R. fortunei* | silica gel-dried | wild | RSBG#2017/143 |
| RSF34 | *R. lindleyi* | *R. lindleyi* KR 8074 | 2*x* | 75.17 | 6.53 | n/a | n/a | *R. fortunei* | silica gel-dried | wild: India | RSBG#2008/100 |
| RSF35 | *R. lindleyi* | *R. lindleyi* SEH#534 | 2*x* | 74.16 | 8.21 | n/a | n/a | *R. fortunei* | silica gel-dried | wild | RSBG#2001/239 |
| RSF36 | *R. ludwigianum* | *R. ludwigianum* | 2*x* | 75.29 | 8.09 | n/a | n/a | *R. fortunei* | silica gel-dried | wild | RSBG#2004/030 |
| RSF37 | *R. lyi* | *R. lyi* KR 2962 | 2*x* | 75.38 | 6.9 | n/a | n/a | *R. fortunei* | silica gel-dried | wild: Vietnam | RSBG#1996/060 |
| RSF38 | *R. lyi* | *R. lyi* KR Valder# | 2*x* | 79.34 | 6.79 | n/a | n/a | *R. fortunei* | silica gel-dried | wild: Vietnam (? probably Thailand) | RSBG#2001/146 |
| RSF39 | *R. lyi* | *R. lyi* FMWJ#13042 | 2*x* | 78.11 | 8.34 | n/a | n/a | *R. fortunei* | silica gel-dried | wild | RSBG#2015/018 |
| RSF40 | *R.* aff. *lyi* | *R. lyi affinity* NV#03 | 2*x* | 81.1 | 8.16 | n/a | n/a | *R. fortunei* | silica gel-dried | wild | RSBG#2019/083 |
| RSF41 | *R. maddenii* ssp. *crassum* | *R. maddenii* ssp. *crassum* | 8*x* | 43.25 | 9.08 | 181.55 | 3.48 | *R. fortunei* | silica gel-dried | wild | RSBG#2011/120 |
| RSF42 | *R. maddenii* | *R. maddenii* KCSH#0345 | 6*x* | 38.11 | 6.52 | 109.27 | 7.31 | *R. fortunei* | silica gel-dried | wild: Arunachal Pradesh, India | RSBG#2006/393 |
| RSF43 | *R. maddenii* ssp. *maddenii* | *R. maddenii* ssp. *maddenii* SEH#599 | 6*x* | 45.45 | 8.93 | 125.85 | 6.98 | *R. fortunei* | silica gel-dried | wild: Sikkim, India | RSBG#2001/192 |
| RSF44 | *R. maddenii* | *R. maddenii* | 8*x* | 44.61 | 8.92 | 179.51 | 9.76 | *R. fortunei* | silica gel-dried | wild | RSBG#2005/237 |
| RSF45 | *R. maddenii* | *R. maddenii* | 6*x* | 44 | 9.83 | 127.67 | 4 | *R. fortunei* | silica gel-dried | wild | RSBG#2006/153 |
| RSF46 | *R. maddenii* | *R. maddenii* | 2*x* | 44.18 | 8.72 | n/a | n/a | *R. fortunei* | silica gel-dried | wild | RSBG#2008/046 |
| RSF47 | *R. maddenii* ssp. *crassum* | *R. maddenii* ssp. *crassum* | 2*x* | 46.2 | 7.95 | n/a | n/a | *R. fortunei* | silica gel-dried | wild | RSBG#2010/005 |
| RSF48 | *R. maddenii* ssp. *crassum* | *R. maddenii* ssp. *crassum* | 2*x* | 49.47 | 8.72 | n/a | n/a | *R. fortunei* | silica gel-dried | wild | RSBG#1999/631 |
| RSF49 | *R. maddenii* ssp. *crassum* | *R. maddenii* ssp. *crassum* | 8*x* | 50.18 | 7.93 | 199.1 | 5.47 | *R. fortunei* | silica gel-dried | wild | RSBG#2007/273 |
| RSF50 | *R. maddenii* ssp. *crassum* | *R. maddenii* ssp. *crassum* BASE#9539 | 8*x* | 51.17 | 6.21 | 206.78 | 4.12 | *R. fortunei* | silica gel-dried | wild: China | RSBG#2009/017 |
| RSF51 | *R. maddenii* ssp. *crassum* | *R. maddenii* ssp. *crassum* KR#3116 | 2*x* | 51.7 | 6.94 | n/a | n/a | *R. fortunei* | silica gel-dried | wild: Vietnam | RSBG#1996/027 |
| RSF52 | *R. maddenii* ssp. *crassum* | *R. maddenii* ssp. *crassum* DJHM#13057 | 8*x* | 50.45 | 6.76 | 199.02 | 5.03 | *R. fortunei* | silica gel-dried | wild: Burma | RSBG |
| RSF53 | *R. megacalyx* | *R. megacalyx* BASE 9544 | 2*x* | 51.38 | 7.05 | n/a | n/a | *R. fortunei* | silica gel-dried | wild: China | RSBG#2005/243 |
| RSF54 | *R. megacalyx* | *R. megacalyx* DGEY#026 | 2*x* | 51.62 | 5.86 | n/a | n/a | *R. fortunei* | silica gel-dried | wild | RSBG#2006/304 |
| RSF55 | *R. nuttallii* | *R. nuttallii* DGEY 079 | 2*x* | 52.5 | 7.68 | n/a | n/a | *R. fortunei* | silica gel-dried | wild: China | RSBG#2004/374 |
| RSF56 | *R. nuttallii* | *R. nuttallii* HECC#10005 | 2*x* | 49.76 | 8.13 | n/a | n/a | *R. fortunei* | silica gel-dried | wild: Arunachal Pradesh | RSBG |
| RSF57 | *R. pachypodum* | *R. pachypodum* JN 11046 | 2*x* | 52.3 | 7.27 | n/a | n/a | *R. fortunei* | silica gel-dried | wild: China | RSBG#2014/217 |
| RSF58 | *R. pseudociliipes* | *R. pseudociliipes* JN 11070 | 2*x* | 47.59 | 7.4 | n/a | n/a | *R. fortunei* | silica gel-dried | wild: China | RSBG#2014/184 |
| RSF59 | *R. pseudociliipes* | *R. pseudociliipes* BASE 9697 | 2*x* | 55.55 | 8.21 | n/a | n/a | *R. fortunei* | silica gel-dried | wild: China | RSBG#2005/132 |
| RSF60 | *R. pseudociliipes* | *R. pseudociliipes* | 2*x* | 56.16 | 8.79 | n/a | n/a | *R. fortunei* | silica gel-dried | wild: Vietnam | RSBG#2018/162 |
| RSF61 | *R. pseudomaddenii* | *R. pseudomaddenii* HECC#10083 | 2*x* | 42.84 | 12.72 | n/a | n/a | *R. fortunei* | silica gel-dried | wild | RSBG#2017/066 |
| RSF62 | *R. roseatum* | *R. roseatum* GLEN - RBGE | 2*x* | 39.72 | 11.53 | n/a | n/a | *R. fortunei* | silica gel-dried | wild | RSBG#1998/016 |
| RSF63 | *R. scopulorum* | *R. scopulorum* AC 3669 | 2*x* | 41.93 | 11.89 | n/a | n/a | *R. fortunei* | silica gel-dried | wild: Bayi in Tibet, China | RSBG#2005/110 |
| RSF64 | *R. scopulorum* | *R. scopulorum* KW 6354 | 2*x* | 46.7 | 12.34 | n/a | n/a | *R. fortunei* | silica gel-dried | wild: Tibet & Bhutan | RSBG#1998/014 |
| RSF65 | *R. scopulorum* | *R. scopulorum* CC 7571 | 2*x* | 45.02 | 12.08 | n/a | n/a | *R. fortunei* | silica gel-dried | wild: Tibet | RSBG#2002/007 |
| RSF66 | *R. surasianum* | *R. surasianum* (no detail) | 2*x* | 44.13 | 13.34 | n/a | n/a | *R. fortunei* | silica gel-dried | wild | RSBG#2003/177 |
| RSF67 | *R.* aff. *valentinioides* (ined.) | *R. valentinioides* affinity | 2*x* | 42.42 | 13.73 | n/a | n/a | *R. fortunei* | silica gel-dried | wild | RSBG#2014/166 |
| RSF68 | *R. valentinianum* var.*oblongilobatum* | *R. oblongilobatum* | 2*x* | 37.54 | 12.57 | n/a | n/a | *R. fortunei* | silica gel-dried | wild | RSBG#2014/211 |
| RSF69 | *R. valentinioides* (ined.) | *R. valentinioides* | 2*x* | 35.06 | 10.17 | n/a | n/a | *R. fortunei* | silica gel-dried | wild | RSBG#1997/139 |
| RSF70 | *R. veitchianum* | *R. veitchianum* C 9001 | 2*x* | 41.85 | 13.63 | n/a | n/a | *R. fortunei* | silica gel-dried | wild: Thailand | RSBG#2002/012 |
| RSF71 | *R. veitchianum* | *R. veitchianum* | 2*x* | 43.33 | 11.5 | n/a | n/a | *R. fortunei* | silica gel-dried | wild | RSBG#1998/017 |
| RSF72 | *R. walongense* | *R. walongense* HECC 10004 | 2*x* | 44.18 | 10.86 | n/a | n/a | *R. fortunei* | silica gel-dried | wild: India | RSBG#1998/003 |
| RSF73 | *R. walongense* | *R. walongense* HECC 10006 | 2*x* | 44.1 | 11.69 | n/a | n/a | *R. fortunei* | silica gel-dried | wild: India | RSBG#2005/253 |
| RSF74 | *R. wumingense* | *R. wumingense* CGG 14150 | 2*x* | 43.51 | 13.51 | n/a | n/a | *R. fortunei* | silica gel-dried | wild | RSBG#2014/111 |
