# Supplementary material for: Ploidy variation in Rhododendron subsection Maddenia and its implications for conservation
Source: AoB Plants. 2023 Apr 12;15(3):plad016. doi: 10.1093/aobpla/plad016 (PMC10184449; doi:10.1093/aobpla/plad016)

**Table S3.** Flow cytometry histograms of subsection *Maddenia* accessions with inconsistent ploidy in different runs. Samples are listed in alphabetical order of species names. Histogram graphs for each of the accessions are presented below the table correspondingly.

| **Taxon** | **Sample** | **FCM ploidy** | **Diploid standard** | **Leaf sample** | **Mean-x (RN1)** | **CV-x% (RN1)** | **Mean-x (RN2)** | **CV-x% (RN2)** | **FCM date** |
| --- | --- | --- | --- | --- | --- | --- | --- | --- | --- |
| *R. excellens* | OM34 | 2*x* | *R. fortunei* (OM60) | fresh | 62.49 | 6 | n/a | n/a | 23-02-2021 (left) |
|  |  | 2*x* | *R. fortunei* (OM60) | silica gel-dried | 64.86 | 6.15 | n/a | n/a | 18-05-2021 (middle) |
|  |  | Aneuploid lower than diploid standard? | *R. fortunei* (OM60) | Herbarium | 48.46 | 5.85 | 33 | 7.35 | 27-05-2022 (right) |
| 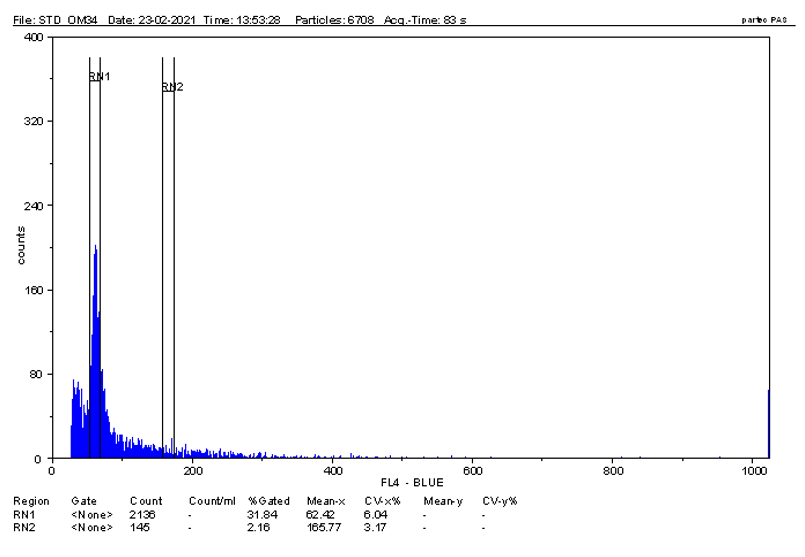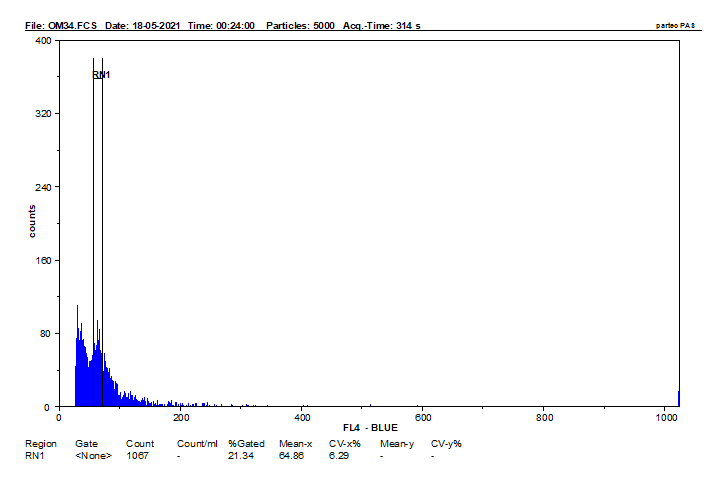 |  |  |  |  |  |  |  |  |  |
|  |  |  |  |  |  |  |  |  |  |
|  |  |  |  |  |  |  |  |  |  |
|  |  |  |  |  |  |  |  |  |  |
|  |  |  |  |  |  |  |  |  |  |
|  |  |  |  |  |  |  |  |  | 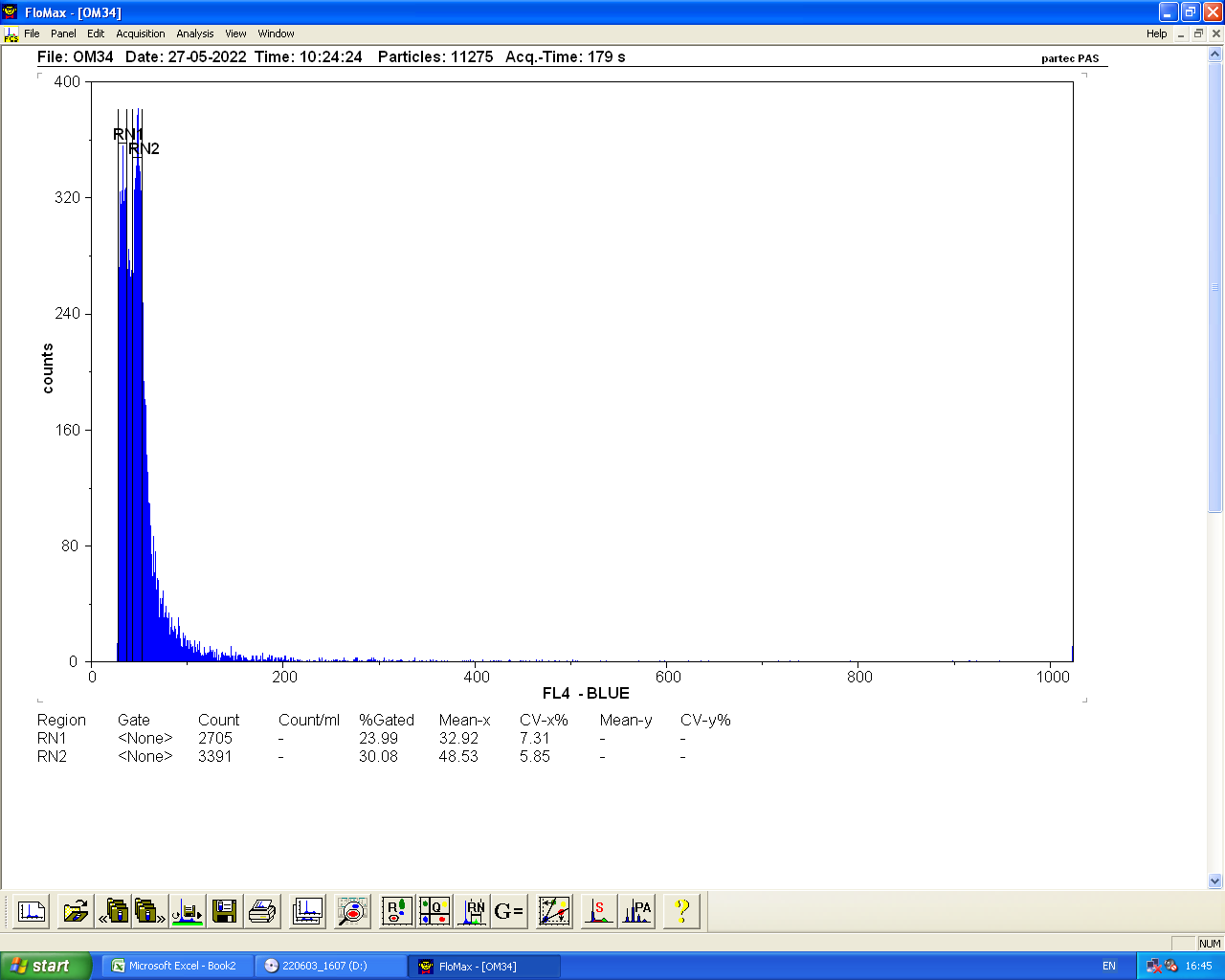 |
|  |  |  |  |  |  |  |  |  |  |
|  |  |  |  |  |  |  |  |  |  |
|  |  |  |  |  |  |  |  |  |  |
|  |  |  |  |  |  |  |  |  |  |
|  |  |  |  |  |  |  |  |  |  |
|  |  |  |  |  |  |  |  |  |  |
| *R. maddenii* ssp. *crassum* | OM47 | 8*x* | *R. fortunei* (OM60) | fresh | 57.87 | 6.34 | 228.08 | 2.63 | 23-02-2021 (left) |
|  |  | 8*x* | *R. fortunei* (OM60) | silica gel-dried | 60.13 | 6.2 | 230.52 | 2.14 | 18-05-2021 (middle) |
|  |  | 2*x* | *R. fortunei* (OM60) | Herbarium | 38.46 | 9.12 | n/a | n/a | 27-05-2022 (right) |
| 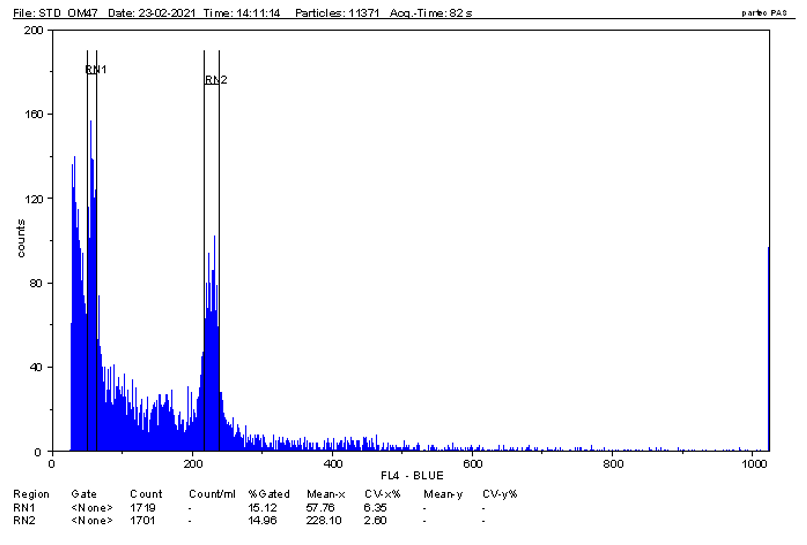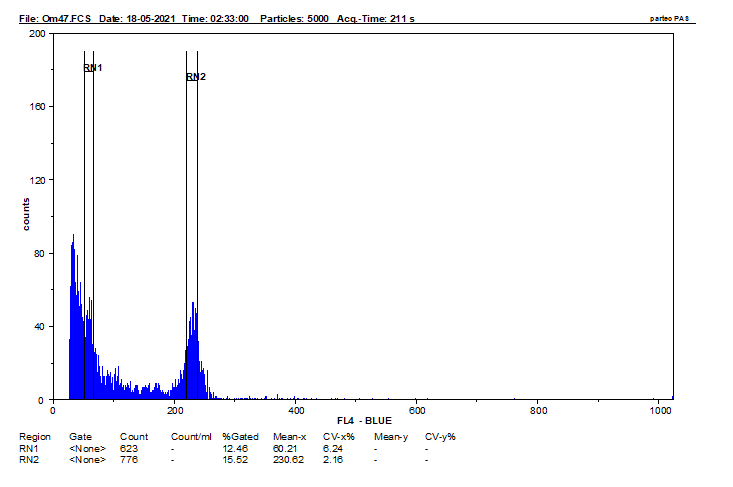 |  |  |  |  |  |  |  |  |  |
|  |  |  |  |  |  |  |  |  |  |
|  |  |  |  |  |  |  |  |  |  |
|  |  |  |  |  |  |  |  |  | 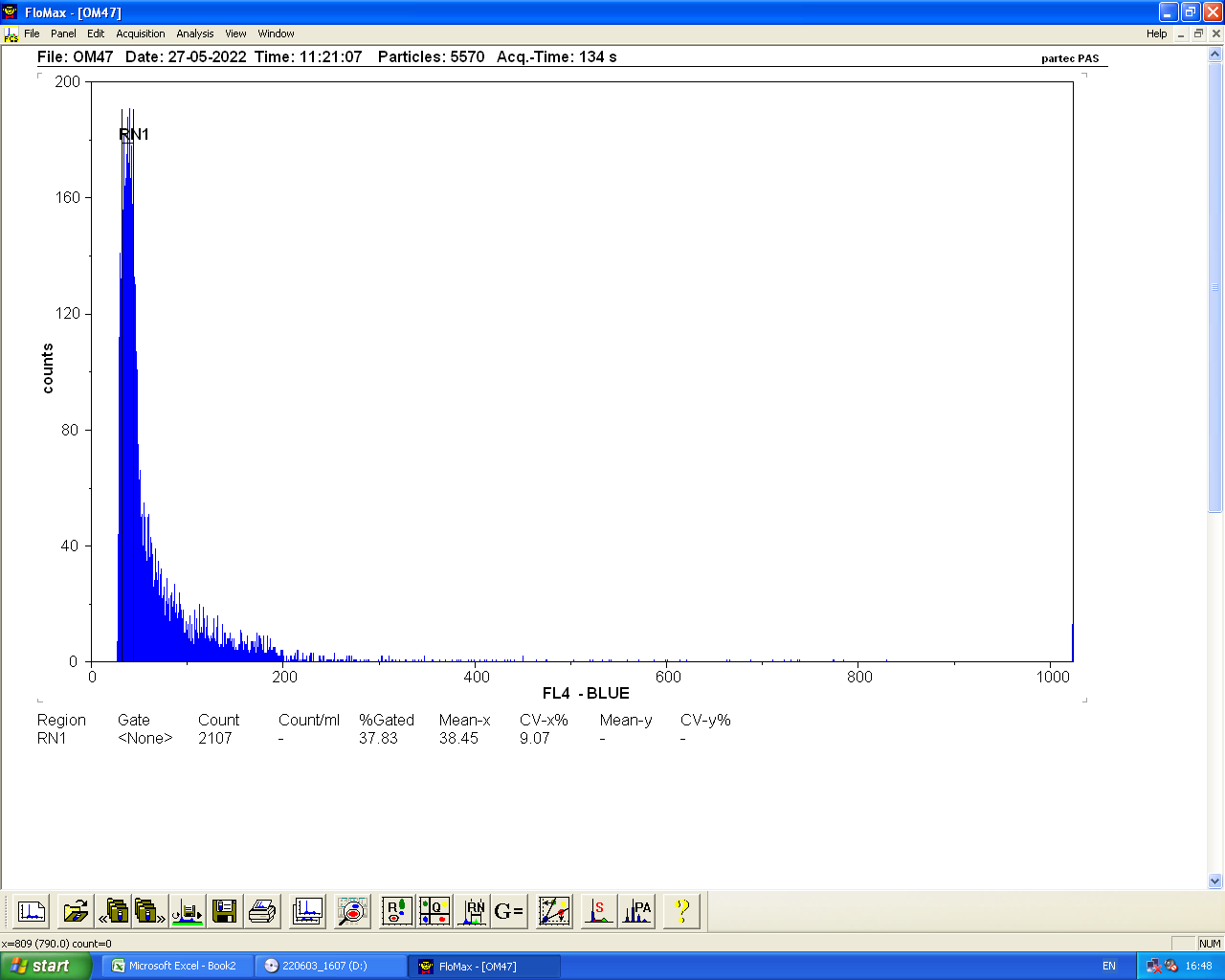 |
|  |  |  |  |  |  |  |  |  |  |
|  |  |  |  |  |  |  |  |  |  |
|  |  |  |  |  |  |  |  |  |  |
|  |  |  |  |  |  |  |  |  |  |
|  |  |  |  |  |  |  |  |  |  |
|  |  |  |  |  |  |  |  |  |  |
| *R. maddenii* ssp. *maddenii* | OM02 | 6*x* | *R. fortunei* (OM60) | fresh | 63.14 | 6.78 | 178.28 | 2.77 | 23-02-2021 (left) |
|  |  | 6*x* | *R. fortunei* (OM60) | silica gel-dried | 66.4 | 3.66 | 184.92 | 2.36 | 18-05-2021 (middle) |
|  |  | 5*x* | *R. fortunei* (OM60) | Herbarium | 30.76 | 12.21 | 78.66 | 6.02 | 25-05-2022 (right) |
|  |  |  |  |  |  |  |  |  |  |
|  |  |  |  |  |  |  |  |  |  |
|  |  |  |  |  |  |  |  |  |  |
|  |  |  |  |  |  |  |  |  |  |
|  |  |  |  |  |  |  |  |  |  |
|  |  | 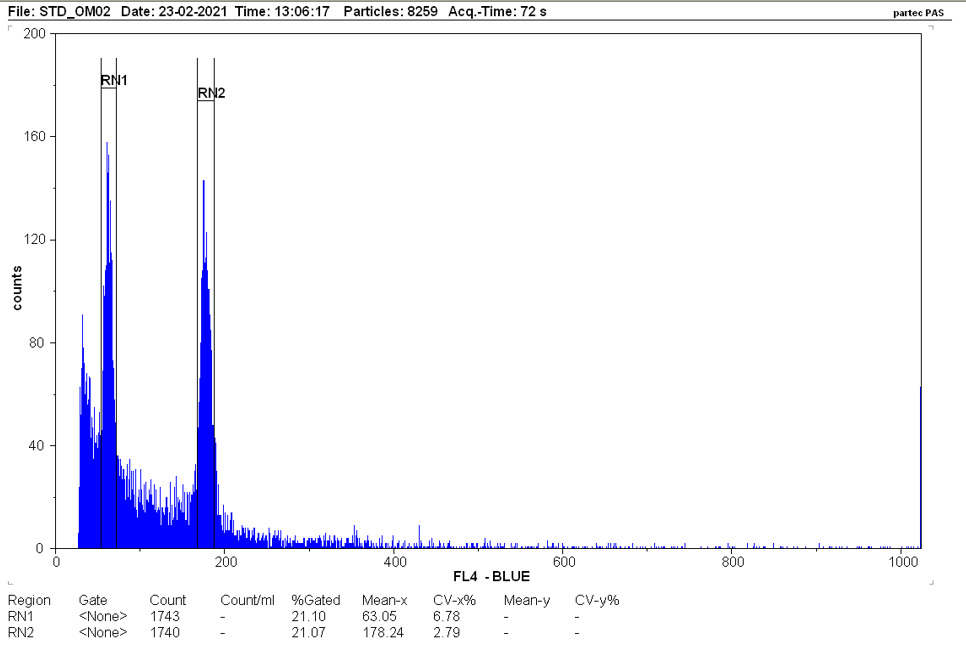 |  |  | 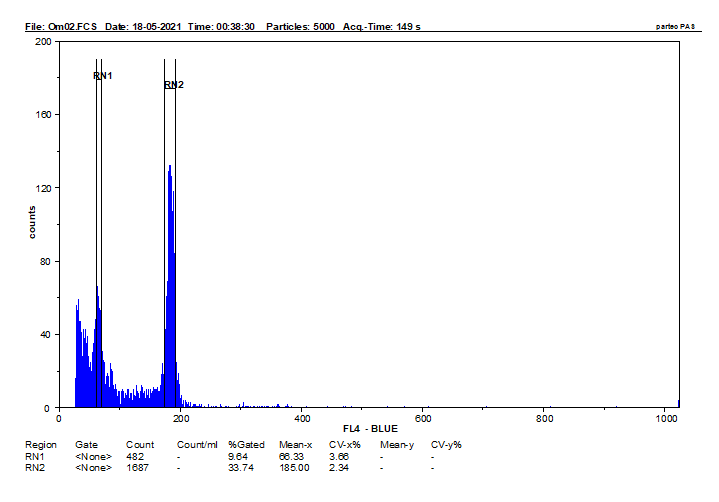 |  |  |  |  |
|  |  |  |  |  |  |  |  | 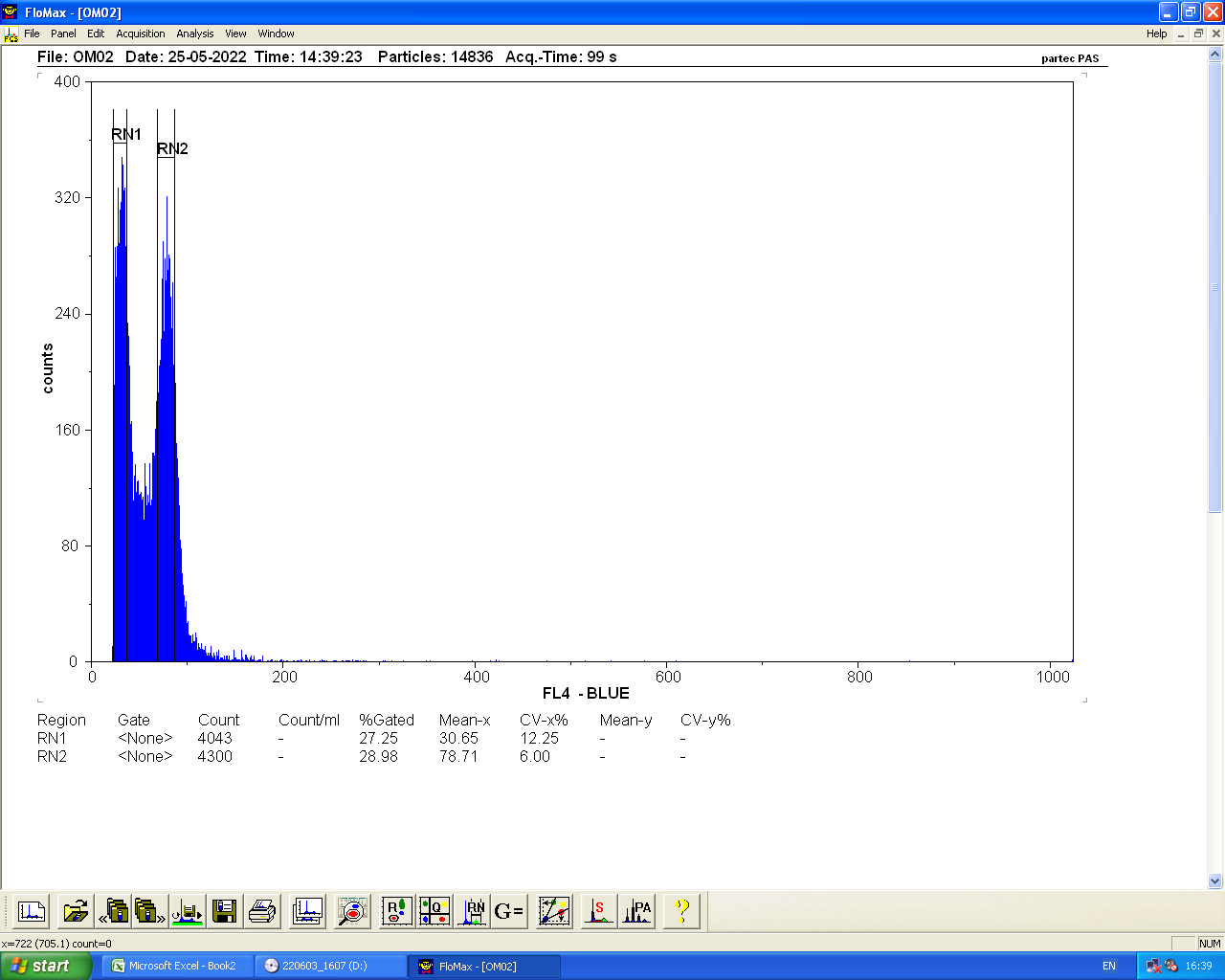 |  |
|  |  |  |  |  |  |  |  |  |  |
|  |  |  |  |  |  |  |  |  |  |
|  |  |  |  |  |  |  |  |  |  |
|  |  |  |  |  |  |  |  |  |  |
|  |  |  |  |  |  |  |  |  |  |
|  |  |  |  |  |  |  |  |  |  |
|  |  |  |  |  |  |  |  |  |  |
| *R. maddenii* ssp. *maddenii* | OM14 | 8*x* | *R. fortunei* (OM60) | fresh | 62.33 | 6.37 | 250.59 | 2.84 | 23-02-2021 (left) |
|  |  | 8*x* | *R. fortunei* (OM60) | silica gel-dried | 62.34 | 5.32 | 244.2 | 2.57 | 18-05-2021 (middle) |
|  |  | 2*x*? / 8*x*? | *R. fortunei* (OM60) | Herbarium | 35.84 | 9.74 | 141.86 | 4.14 | 25-05-2022 (right) |
| 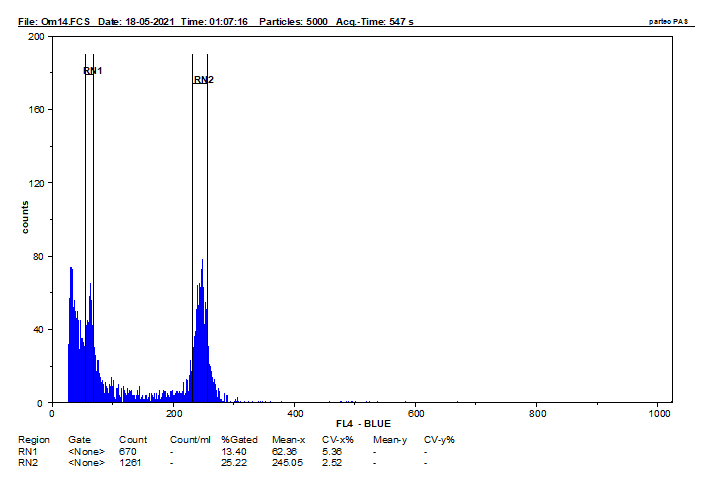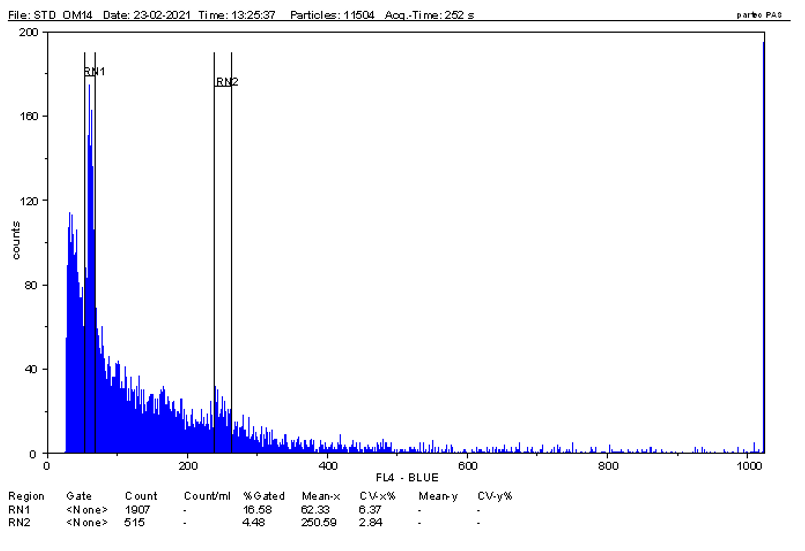 |  |  |  |  |  |  |  |  |  |
|  |  |  |  |  |  |  |  |  |  |
|  |  |  |  |  |  |  |  |  |  |
|  |  |  |  |  |  |  |  |  |  |
|  |  |  |  |  |  |  |  |  |  |
|  |  |  |  |  |  |  |  |  | 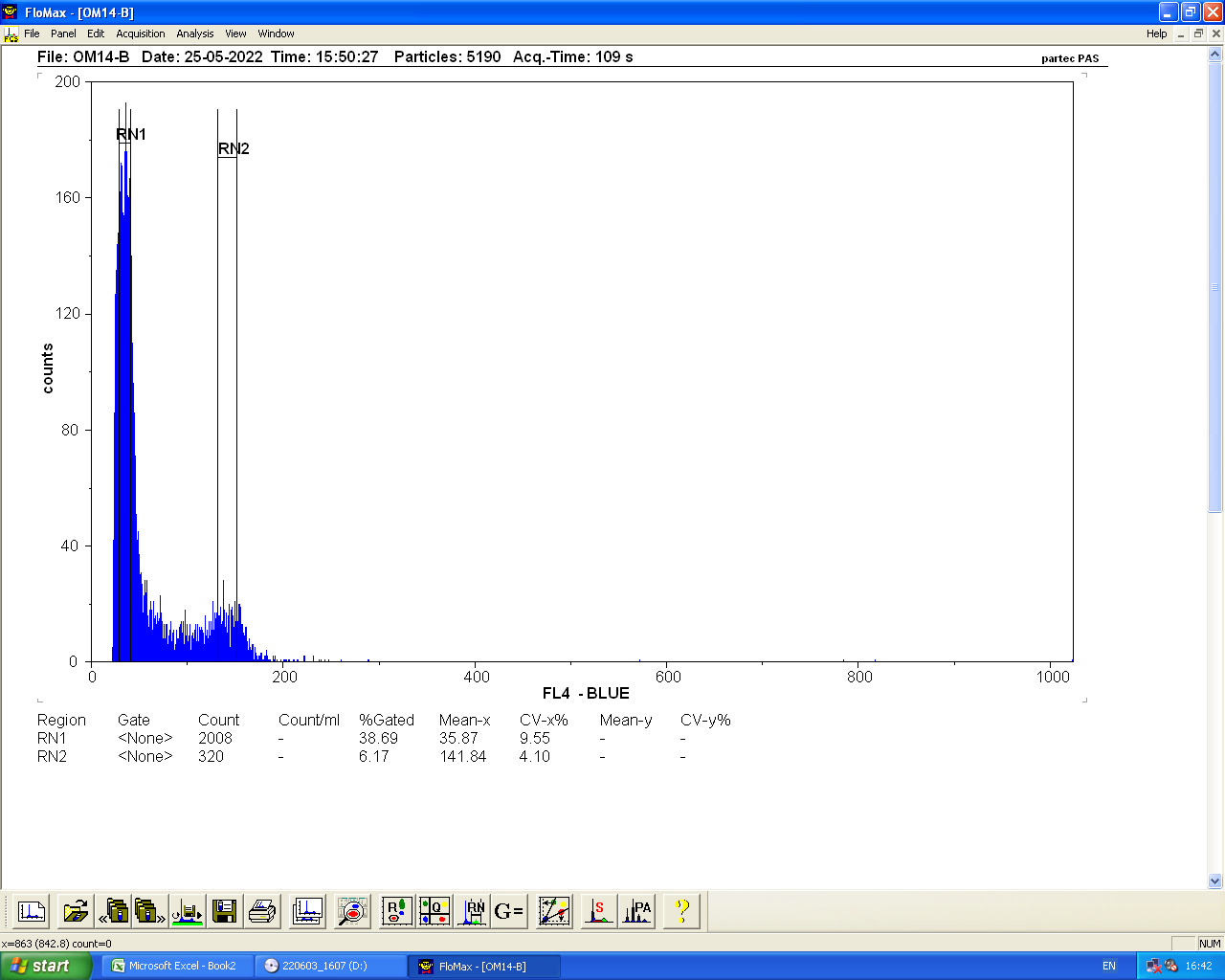 |
|  |  |  |  |  |  |  |  |  |  |
|  |  |  |  |  |  |  |  |  |  |
|  |  |  |  |  |  |  |  |  |  |
|  |  |  |  |  |  |  |  |  |  |
|  |  |  |  |  |  |  |  |  |  |
|  |  |  |  |  |  |  |  |  |  |
|  |  |  |  |  |  |  |  |  |  |
| *R. maddenii* ssp. *maddenii* | OM18 | 6*x* | *R. fortunei* (OM60) | fresh | 61.73 | 6.62 | 174.45 | 3.19 | 23-02-2021 (left) |
|  |  | 6*x* | *R. fortunei* (OM60) | silica gel-dried | 64.08 | 5.44 | 182.84 | 3.36 | 18-05-2021 (middle) |
|  |  | 5*x* | *R. fortunei* (OM60) | Herbarium | 45.18 | 6.75 | 117.76 | 3.32 | 27-05-2022 (right) |
| 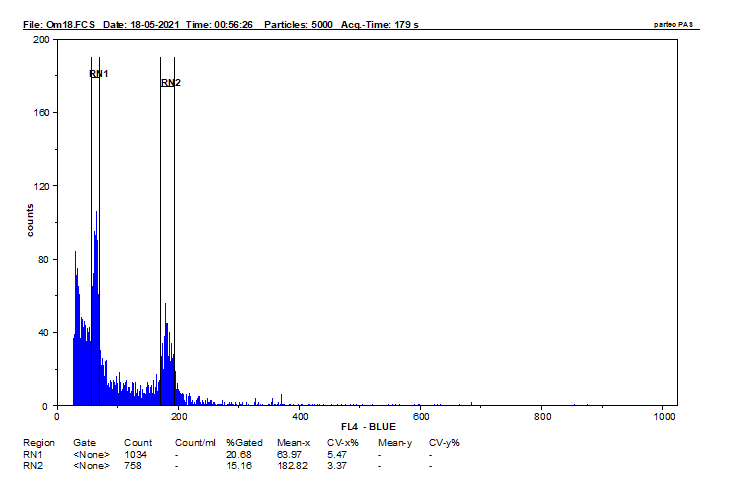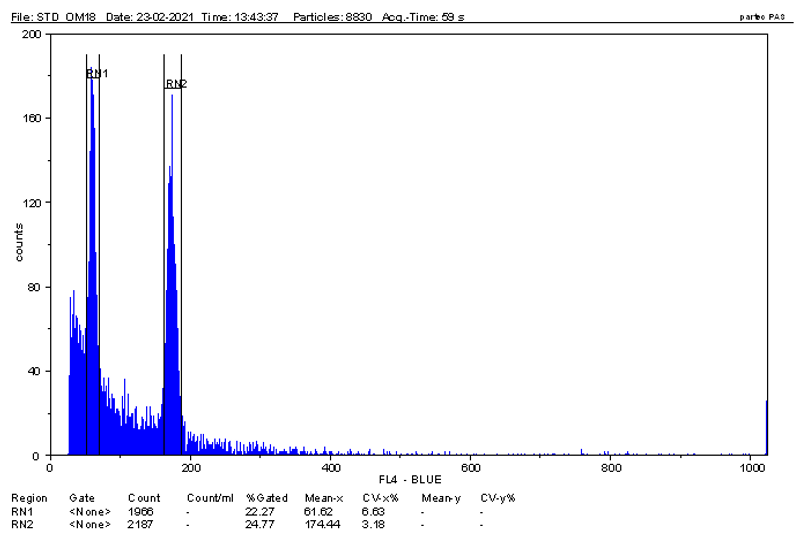 |  |  |  |  |  |  |  |  |  |
|  |  |  |  |  |  |  |  |  |  |
|  |  |  |  |  |  |  |  |  |  |
|  |  |  |  |  |  |  |  |  |  |
|  |  |  |  |  |  |  |  |  |  |
|  |  |  |  |  |  |  |  |  |  |
|  |  |  |  |  |  |  |  | 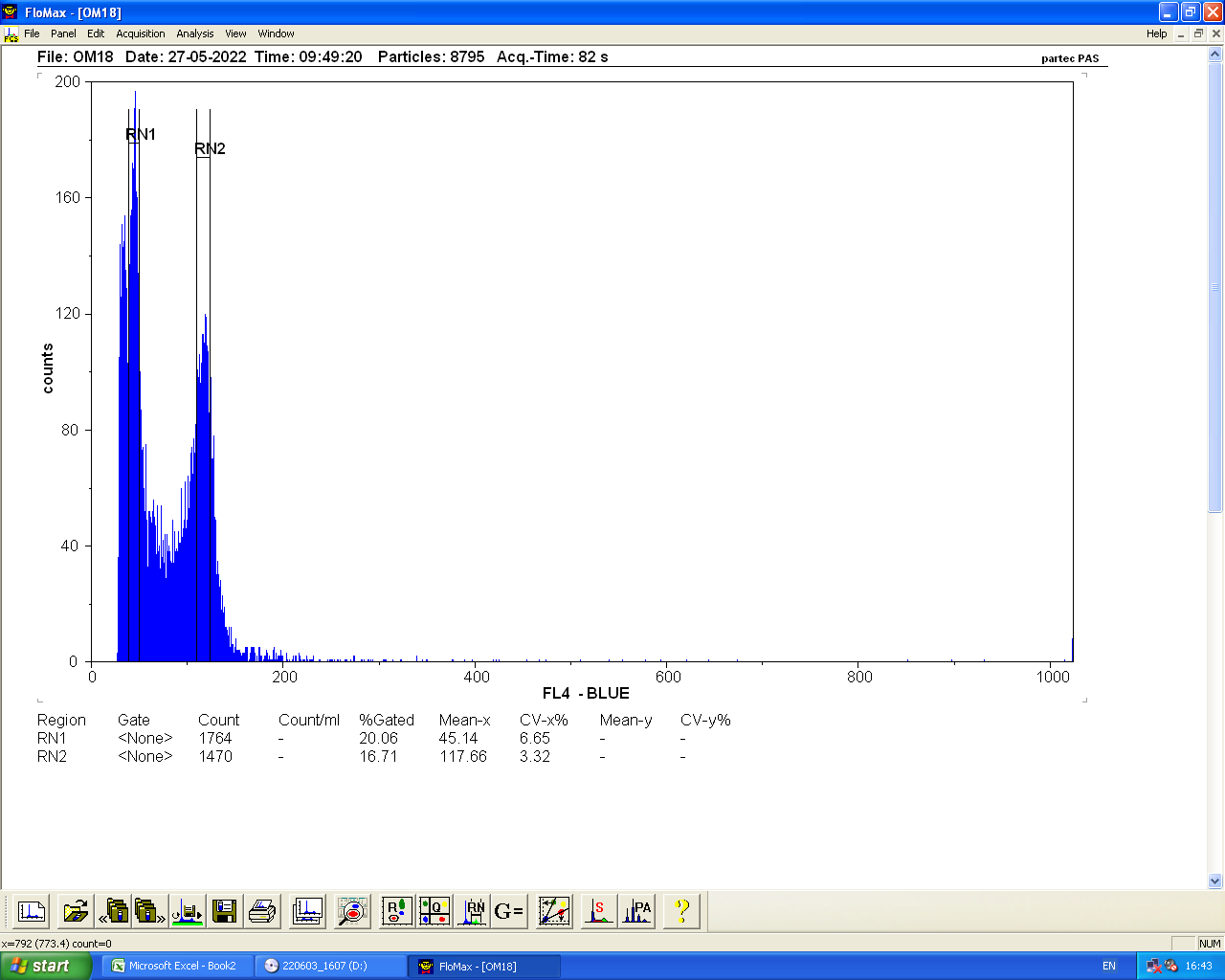 |  |
|  |  |  |  |  |  |  |  |  |  |
|  |  |  |  |  |  |  |  |  |  |
|  |  |  |  |  |  |  |  |  |  |
|  |  |  |  |  |  |  |  |  |  |
|  |  |  |  |  |  |  |  |  |  |
|  |  |  |  |  |  |  |  |  |  |
|  |  |  |  |  |  |  |  |  |  |
| *R. maddenii* ssp. *maddenii* | OM20 | 6*x* | *R. fortunei* (OM60) | fresh | 58.28 | 6.42 | 166.06 | 2.64 | 23-02-2021 (left) |
|  |  | 6*x* | *R. fortunei* (OM60) | silica gel-dried | 56.34 | 5.49 | 158.12 | 3.07 | 18-05-2021 (middle) |
|  |  | 5*x* | *R. fortunei* (OM60) | Herbarium | 42.3 | 6.35 | 108.95 | 5.56 | 27-05-2022 (right) |
| 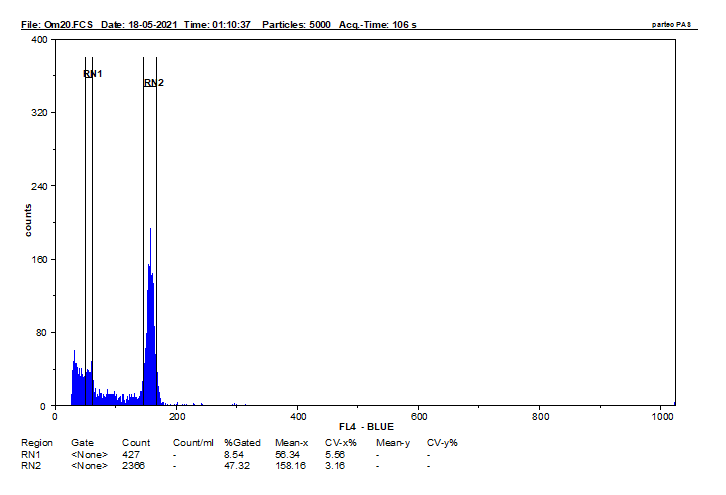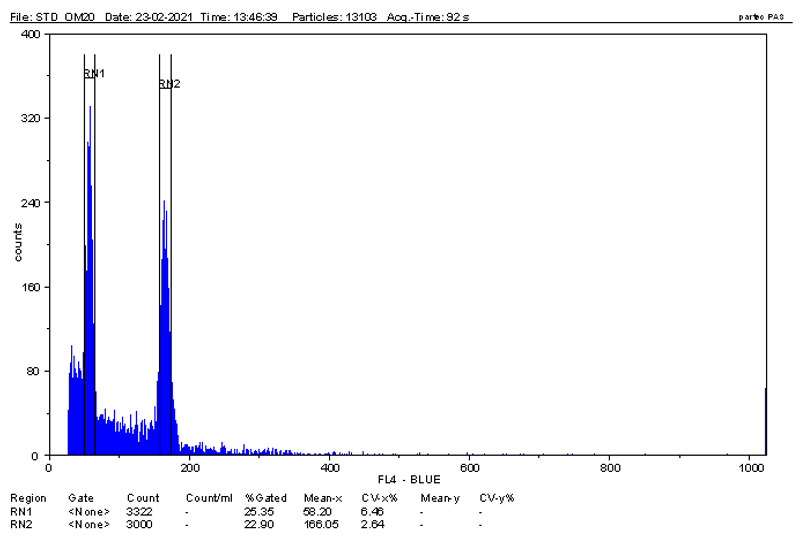 |  |  |  |  |  |  |  |  |  |
|  |  |  |  |  |  |  |  |  |  |
|  |  |  |  |  |  |  |  |  |  |
|  |  |  |  |  |  |  |  |  |  |
|  |  |  |  |  |  |  |  |  |  |
|  |  |  |  |  |  |  |  |  |  |
|  |  |  |  |  |  |  |  |  | 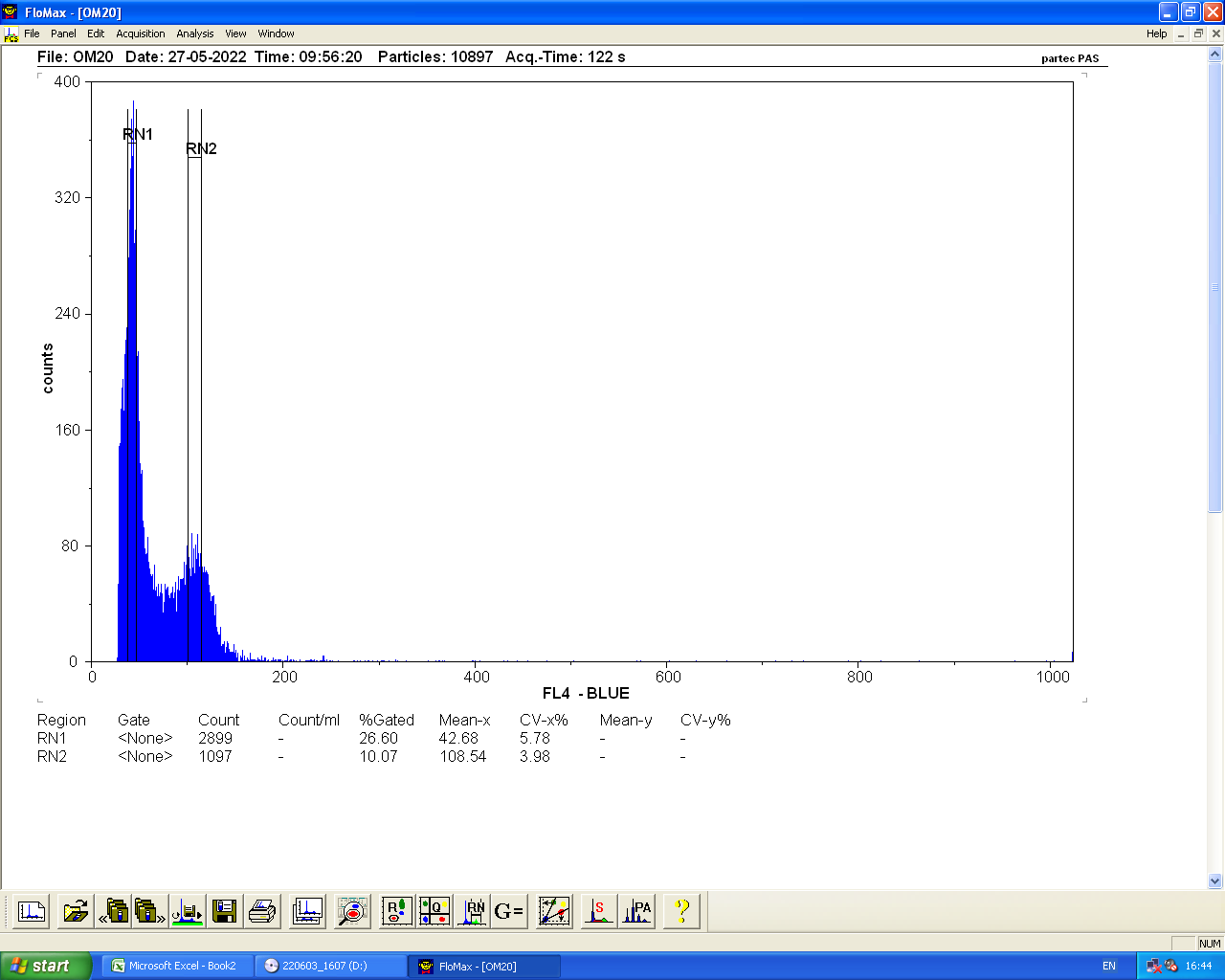 |
|  |  |  |  |  |  |  |  |  |  |
|  |  |  |  |  |  |  |  |  |  |
|  |  |  |  |  |  |  |  |  |  |
|  |  |  |  |  |  |  |  |  |  |
|  |  |  |  |  |  |  |  |  |  |
|  |  |  |  |  |  |  |  |  |  |
| *R. maddenii* ssp. *maddenii* | OM46 | 6*x* | *R. fortunei* (OM60) | fresh | 59.02 | 4.98 | 165.86 | 2.9 | 23-02-2021 (left) |
|  |  | 5-6*x*? | *R. fortunei* (OM60) | silica gel-dried | 61.5 | 6.15 | 166.68 | 3.81 | 18-05-2021 (middle) |
|  |  | 5*x* | *R. fortunei* (OM60) | Herbarium | 45.49 | 5.52 | 112.63 | 2.88 | 27-05-2022 (right) |
| 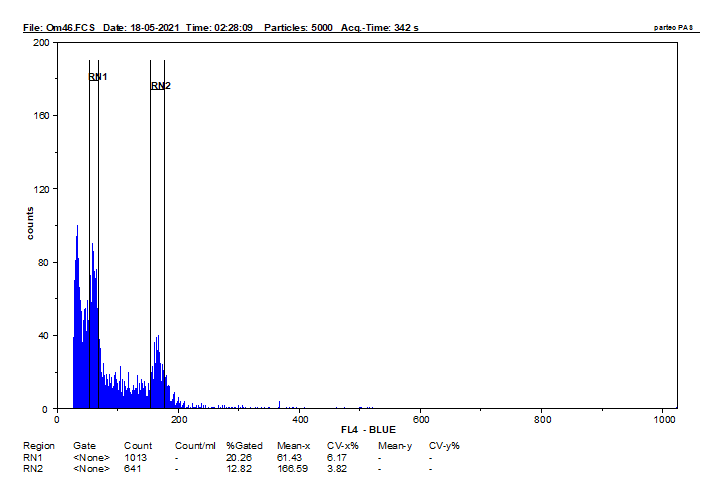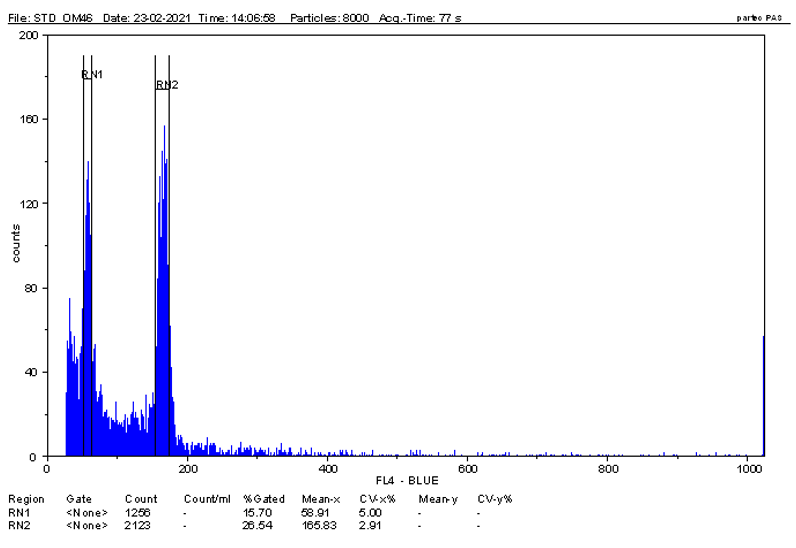 |  |  |  |  |  |  |  |  |  |
|  |  |  |  |  |  |  |  |  |  |
|  |  |  |  |  |  |  |  |  |  |
|  |  |  |  |  |  |  |  |  |  |
|  |  |  |  |  |  |  |  |  | 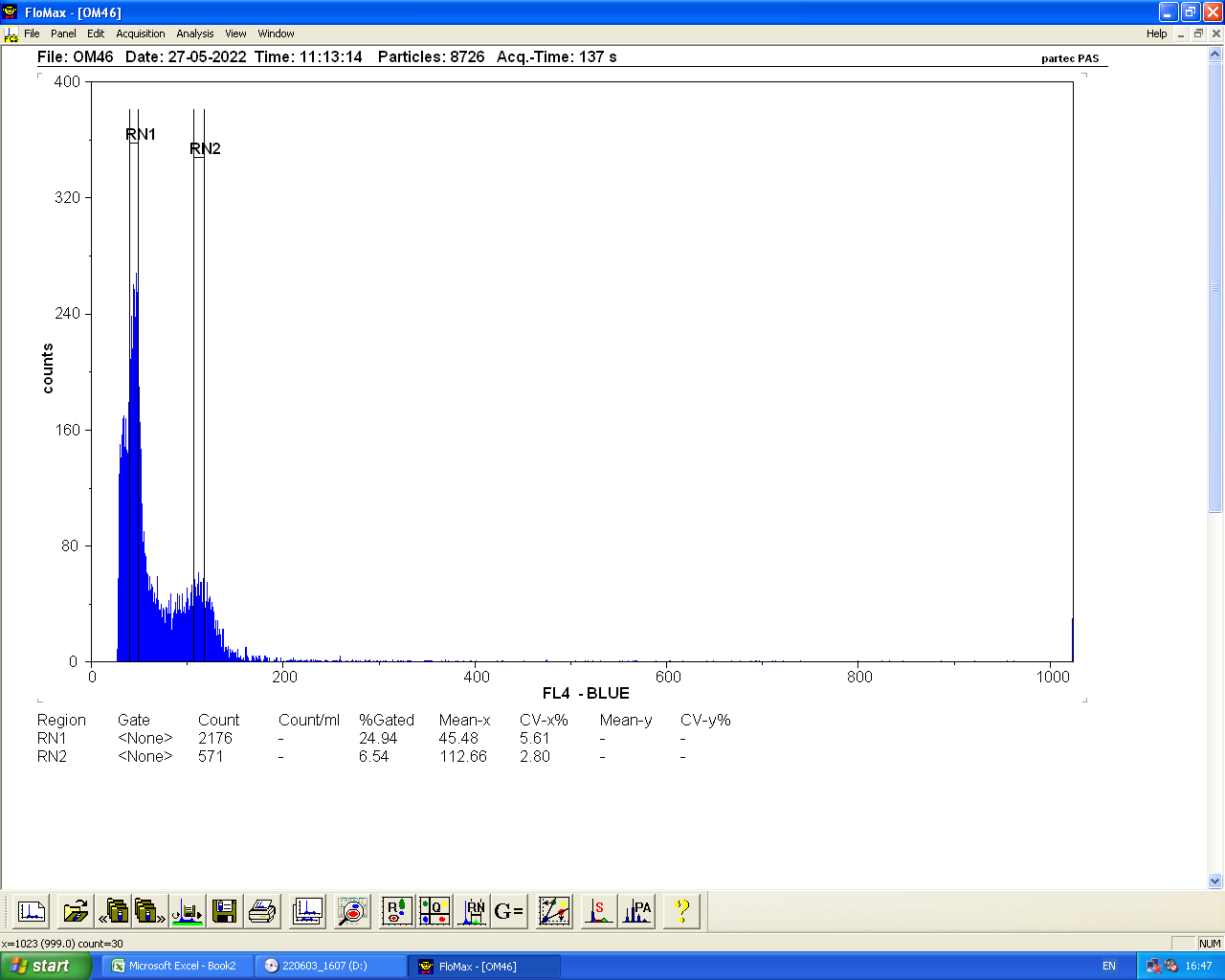 |
|  |  |  |  |  |  |  |  |  |  |
|  |  |  |  |  |  |  |  |  |  |
|  |  |  |  |  |  |  |  |  |  |
|  |  |  |  |  |  |  |  |  |  |
|  |  |  |  |  |  |  |  |  |  |
|  |  |  |  |  |  |  |  |  |  |
|  |  |  |  |  |  |  |  |  |  |
|  |  |  |  |  |  |  |  |  |  |
|  |  |  |  |  |  |  |  |  |  |
| *R. maddenii* ssp. *maddenii* | OM48 | 7*x* | *R. fortunei* (OM60) | fresh | 57.4 | 6.48 | 197.92 | 3.84 | 23-02-2021 (left) |
|  |  | 2x? / 7x? | *R. fortunei* (OM60) | silica gel-dried | 62.64 | 5.62 | 202.26 | 5.32 | 18-05-2021 (middle) |
|  |  | 6-7x | *R. fortunei* (OM60) | Herbarium | 37.05 | 9.41 | 125.97 | 2.87 | 27-05-2022 (right) |
| 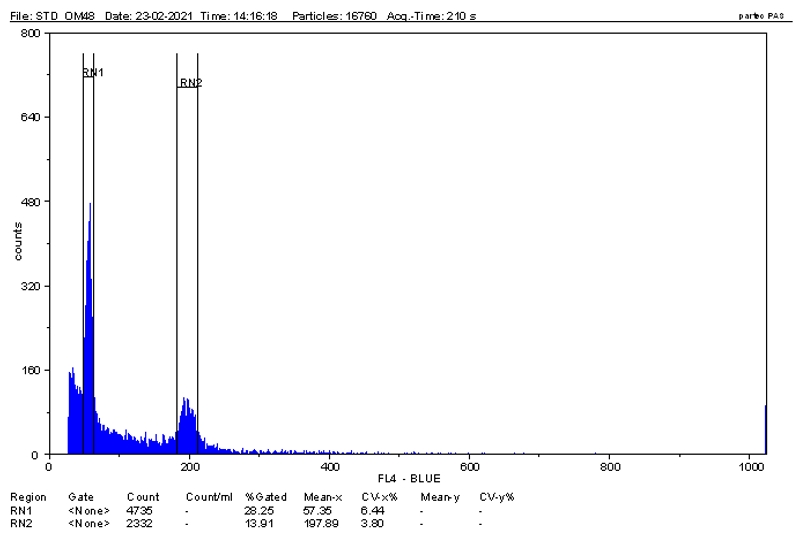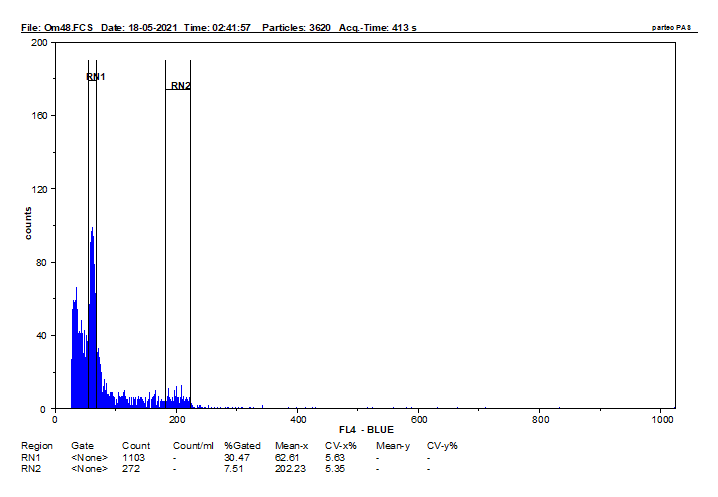 |  |  |  |  |  |  |  |  |  |
|  |  |  |  |  |  |  |  |  |  |
|  |  |  |  |  |  |  |  |  |  |
|  |  |  |  |  |  |  |  |  |  |
|  |  |  |  |  |  |  |  |  |  |
|  |  |  |  |  |  |  |  |  |  |
|  |  |  |  |  |  |  |  |  |  |
|  |  |  |  |  |  |  |  |  | 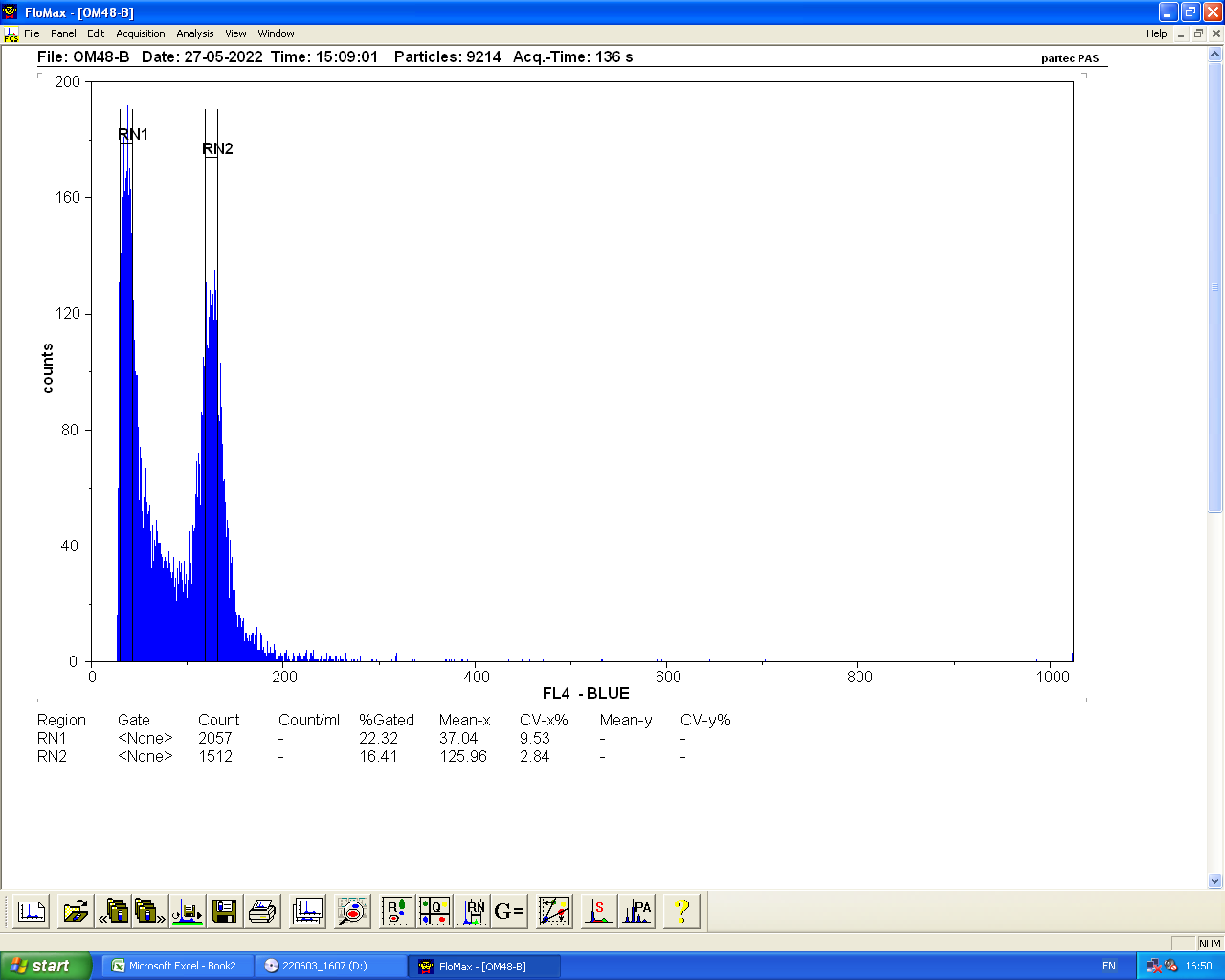 |
|  |  |  |  |  |  |  |  |  |  |
|  |  |  |  |  |  |  |  |  |  |
|  |  |  |  |  |  |  |  |  |  |
|  |  |  |  |  |  |  |  |  |  |
|  |  |  |  |  |  |  |  |  |  |
| *R. maddenii* ssp. *maddenii* | OM49 | 6*x* | *R. fortunei* (OM60) | fresh | 58.46 | 5.54 | 179.98 | 2.86 | 23-02-2021 (left) |
|  |  | 5-6*x*? | *R. fortunei* (OM60) | silica gel-dried | 62.19 | 5.18 | 173.5 | 5.34 | 18-05-2021 (middle) |
|  |  | 6*x* | *R. fortunei* (OM60) | Herbarium | 37.81 | 8 | 111.4 | 4.17 | 27-05-2022 (right) |
| 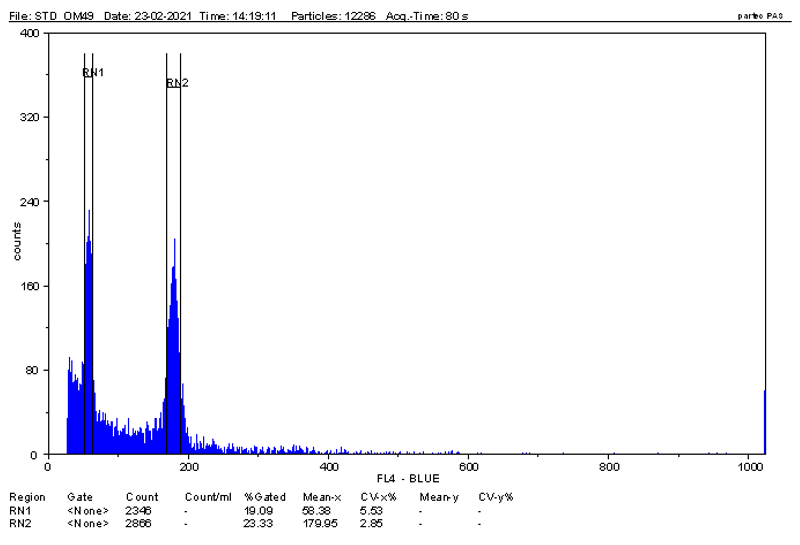 |  |  |  |  |  |  |  |  |  |
|  |  |  |  |  |  |  |  |  |  |
|  |  |  |  |  |  |  |  |  |  |
|  |  |  |  |  |  |  |  |  |  |
|  |  |  |  |  |  |  |  |  |  |
|  |  |  |  |  |  |  |  |  |  |
|  |  |  |  |  | 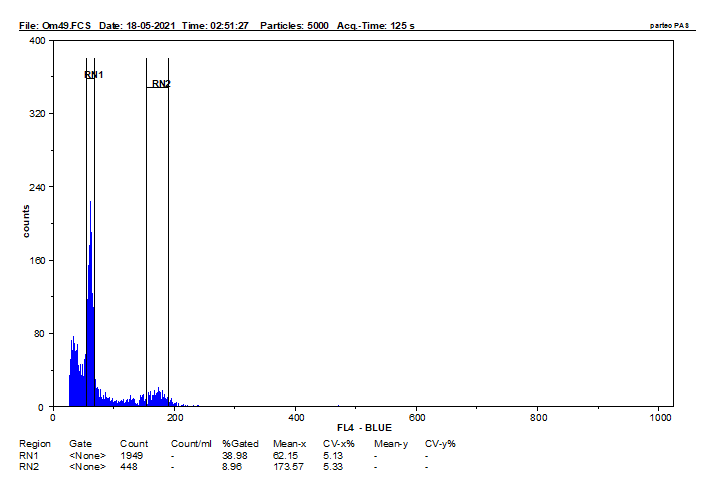 |  |  |  |  |
|  |  |  |  |  |  |  |  |  | 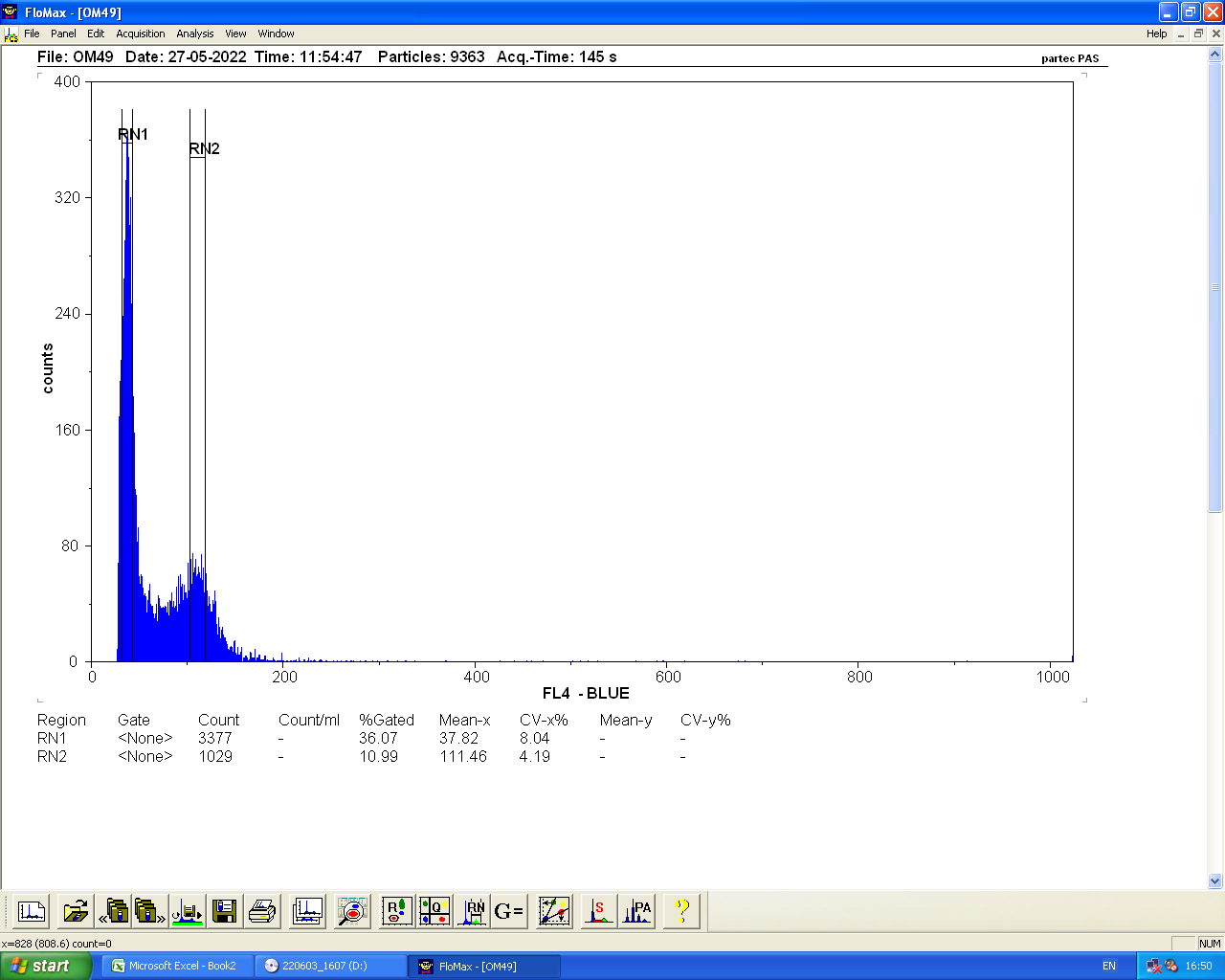 |
|  |  |  |  |  |  |  |  |  |  |
|  |  |  |  |  |  |  |  |  |  |
|  |  |  |  |  |  |  |  |  |  |
|  |  |  |  |  |  |  |  |  |  |
|  |  |  |  |  |  |  |  |  |  |
|  |  |  |  |  |  |  |  |  |  |
| *R. maddenii* ssp. *maddenii* | OM54 | 6*x* | *R. fortunei* (OM60) | fresh | 58.98 | 5.97 | 170.11 | 2.87 | 23-02-2021 (left) |
|  |  | 5-6*x*? | *R. fortunei* (OM60) | silica gel-dried | 63.03 | 4.5 | 173.35 | 3.52 | 18-05-2021 (middle) |
|  |  | 5*x* | *R. fortunei* (OM60) | Herbarium | 44.71 | 6.32 | 117.89 | 2.63 | 27-05-2022 (right) |
| 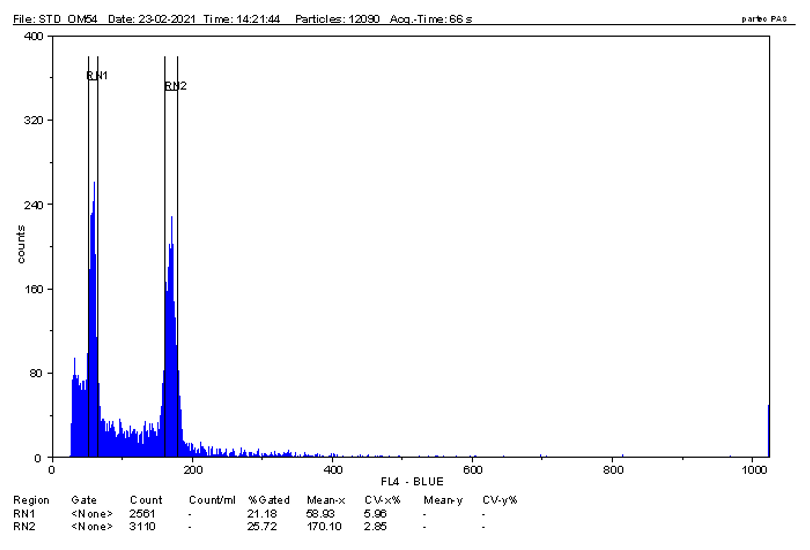 |  |  |  |  |  |  |  |  |  |
|  |  |  |  |  |  |  |  |  |  |
|  |  |  |  |  |  |  |  |  |  |
|  |  |  |  |  |  |  |  |  |  |
|  |  |  |  |  | 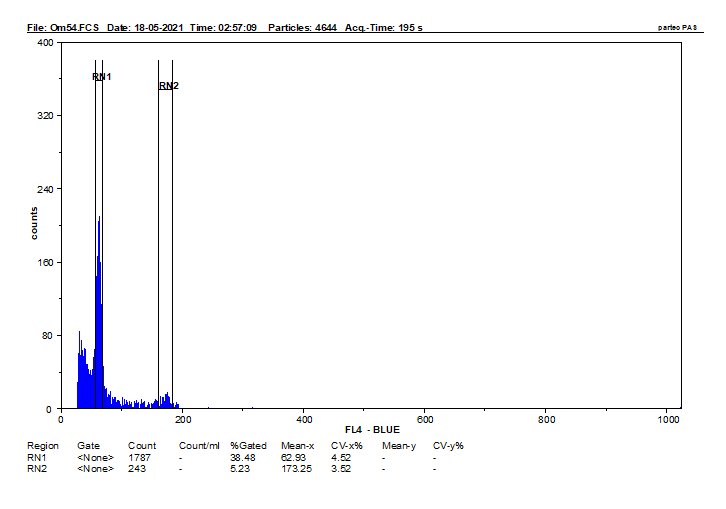 |  |  |  |  |
|  |  |  |  |  |  |  |  | 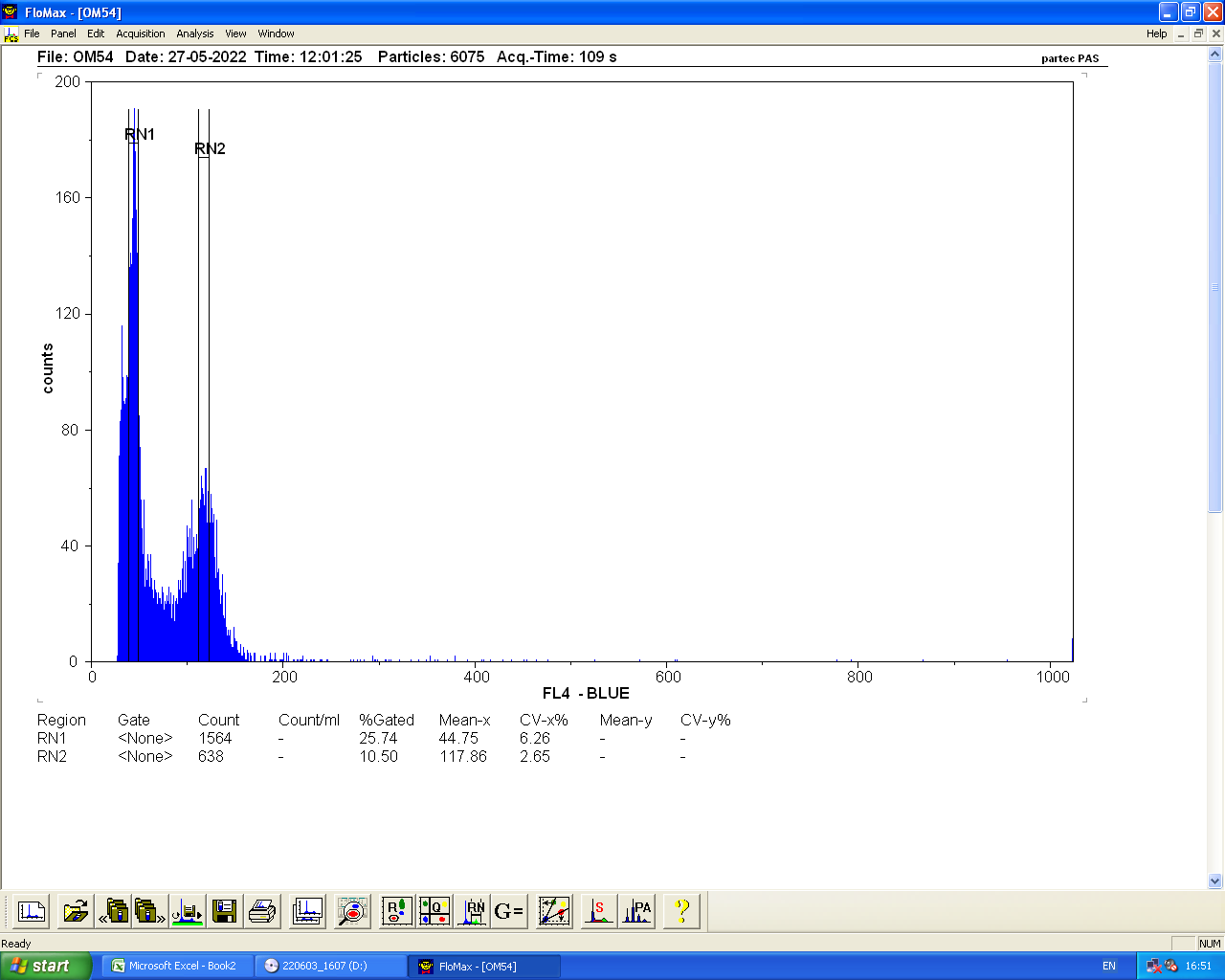 |  |
|  |  |  |  |  |  |  |  |  |  |
|  |  |  |  |  |  |  |  |  |  |
|  |  |  |  |  |  |  |  |  |  |
|  |  |  |  |  |  |  |  |  |  |
|  |  |  |  |  |  |  |  |  |  |
| *R. maddenii* ssp. *maddenii* | OM56 | 6*x* | *R. fortunei* (OM60) | fresh | 58.53 | 6.9 | 167.38 | 3.5 | 23-02-2021 (left) |
|  |  | 6*x* | *R. fortunei* (OM60) | silica gel-dried | 59.76 | 4.54 | 170.32 | 2.59 | 18-05-2021 (middle) |
|  |  | 2*x* | *R. fortunei* (OM60) | Herbarium | 34.88 | 9.43 | n/a | n/a | 27-05-2022 (right) |
| 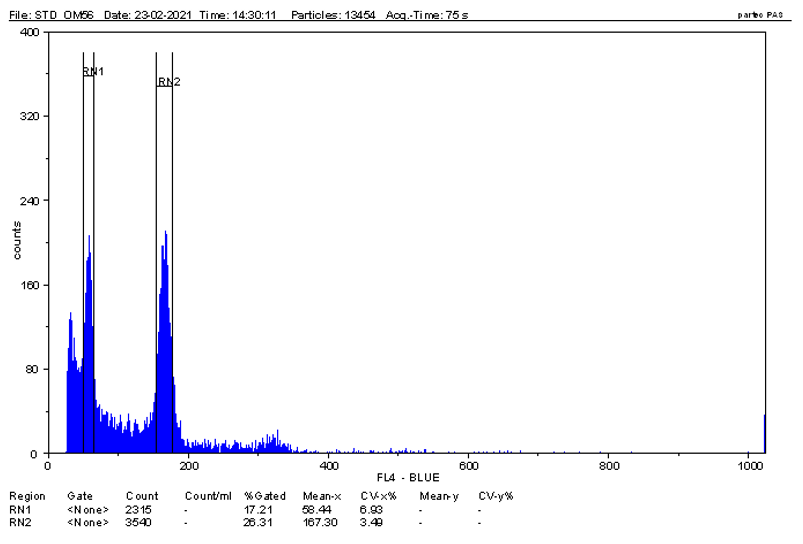 |  |  |  |  |  |  |  |  |  |
|  |  |  |  |  |  |  |  |  |  |
|  |  |  |  |  |  |  |  |  |  |
|  |  |  |  |  |  |  |  |  |  |
|  |  |  |  |  |  |  |  |  |  |
|  |  |  |  |  | 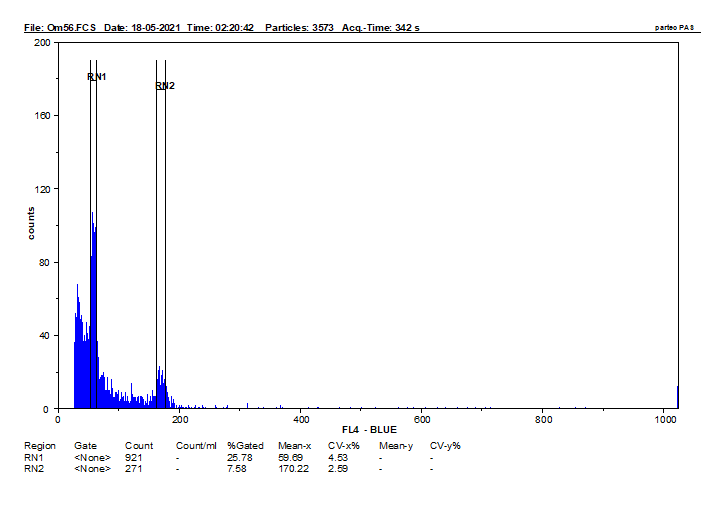 |  |  |  | 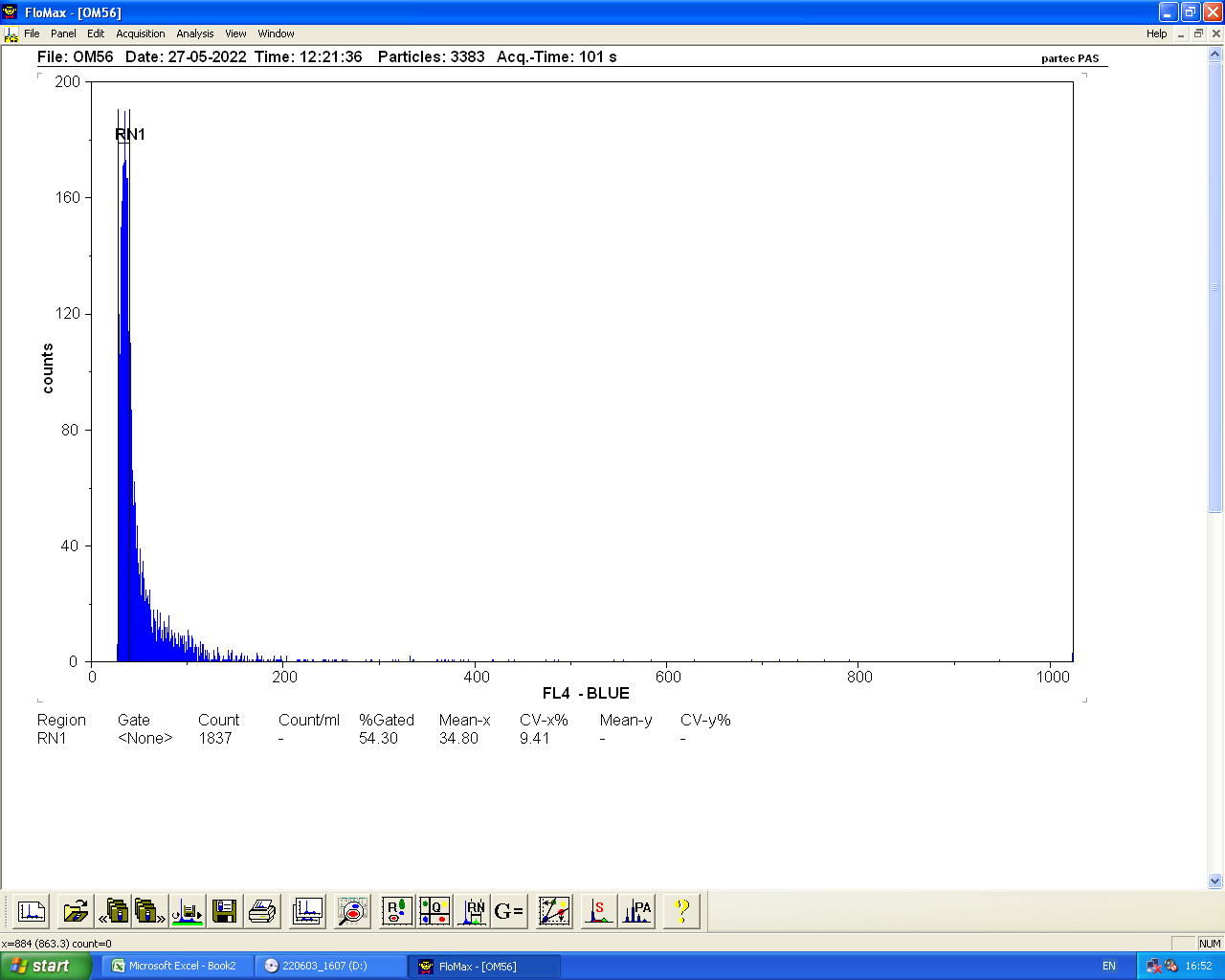 |
|  |  |  |  |  |  |  |  |  |  |
|  |  |  |  |  |  |  |  |  |  |
|  |  |  |  |  |  |  |  |  |  |
|  |  |  |  |  |  |  |  |  |  |
|  |  |  |  |  |  |  |  |  |  |
|  |  |  |  |  |  |  |  |  |  |
|  |  |  |  |  |  |  |  |  |  |
|  |  |  |  |  |  |  |  |  |  |
|  |  |  |  |  |  |  |  |  |  |
| *R. maddenii* ssp. *crassum* | PK22 | 8*x* | *R. fortunei* | fresh | 46.09 | 9.6 | 191.62 | 2.36 | 25-05-2022 (left) |
|  |  | 7*x* | *R. parryae* | fresh | 56.84 | 5.26 | 204.51 | 2.81 | 30-04-2021 (right) |
|  |  |  |  |  |  |  |  |  |  |
|  |  |  |  |  |  |  |  |  |  |
|  |  |  |  |  | 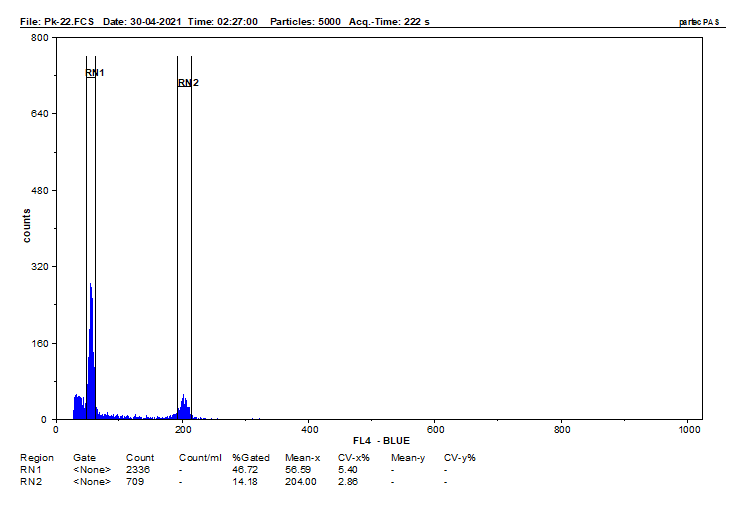 |  |  |  |  |
|  |  |  |  |  |  |  |  |  |  |
|  |  |  |  |  |  |  |  |  |  |
|  |  |  |  |  |  |  |  |  |  |
|  |  |  |  |  |  |  |  |  |  |
|  | 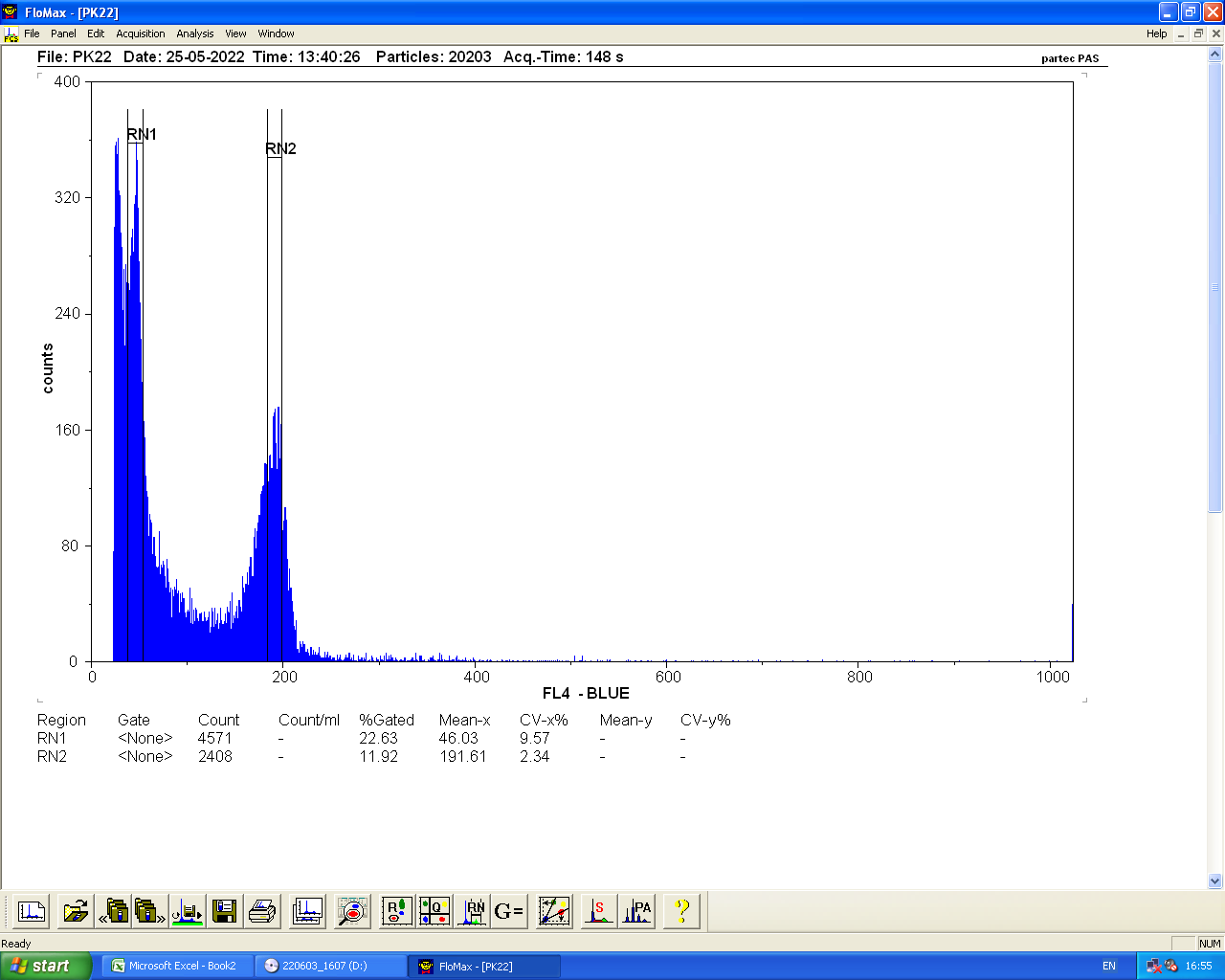 |  |  |  |  |  |  |  |  |
|  |  |  |  |  |  |  |  |  |  |
|  |  |  |  |  |  |  |  |  |  |
|  |  |  |  |  |  |  |  |  |  |
| *R. maddenii* ssp. *crassum* | PK45 | 7*x* | *R. fortunei* | fresh | 45.52 | 6.59 | 165.01 | 2.06 | 25-05-2022 (left) |
|  |  | 6*x* | *R. parryae* | fresh | 67.11 | 4.97 | 212.12 | 2.47 | 30-04-2021 (right) |
|  |  |  |  |  |  |  |  |  |  |
|  |  |  |  |  |  |  |  |  |  |
|  |  |  |  |  |  |  |  |  |  |
|  |  |  |  |  | 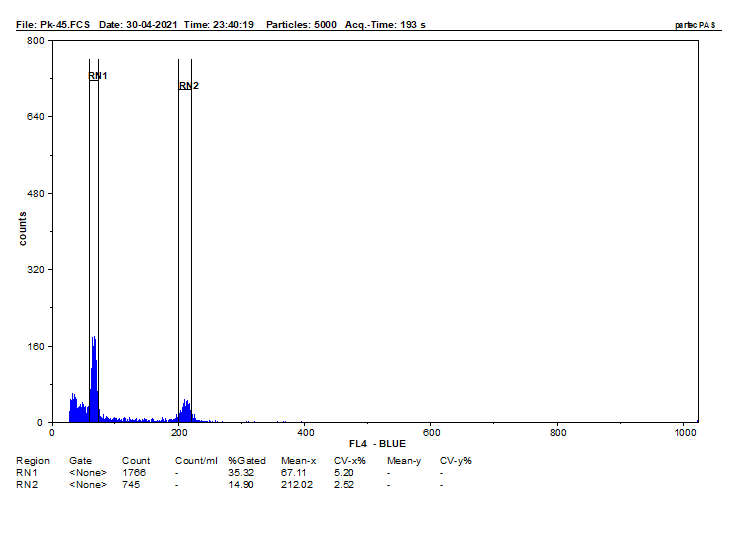 |  |  |  |  |
|  |  |  |  |  |  |  |  |  |  |
|  |  |  |  |  |  |  |  |  |  |
|  | 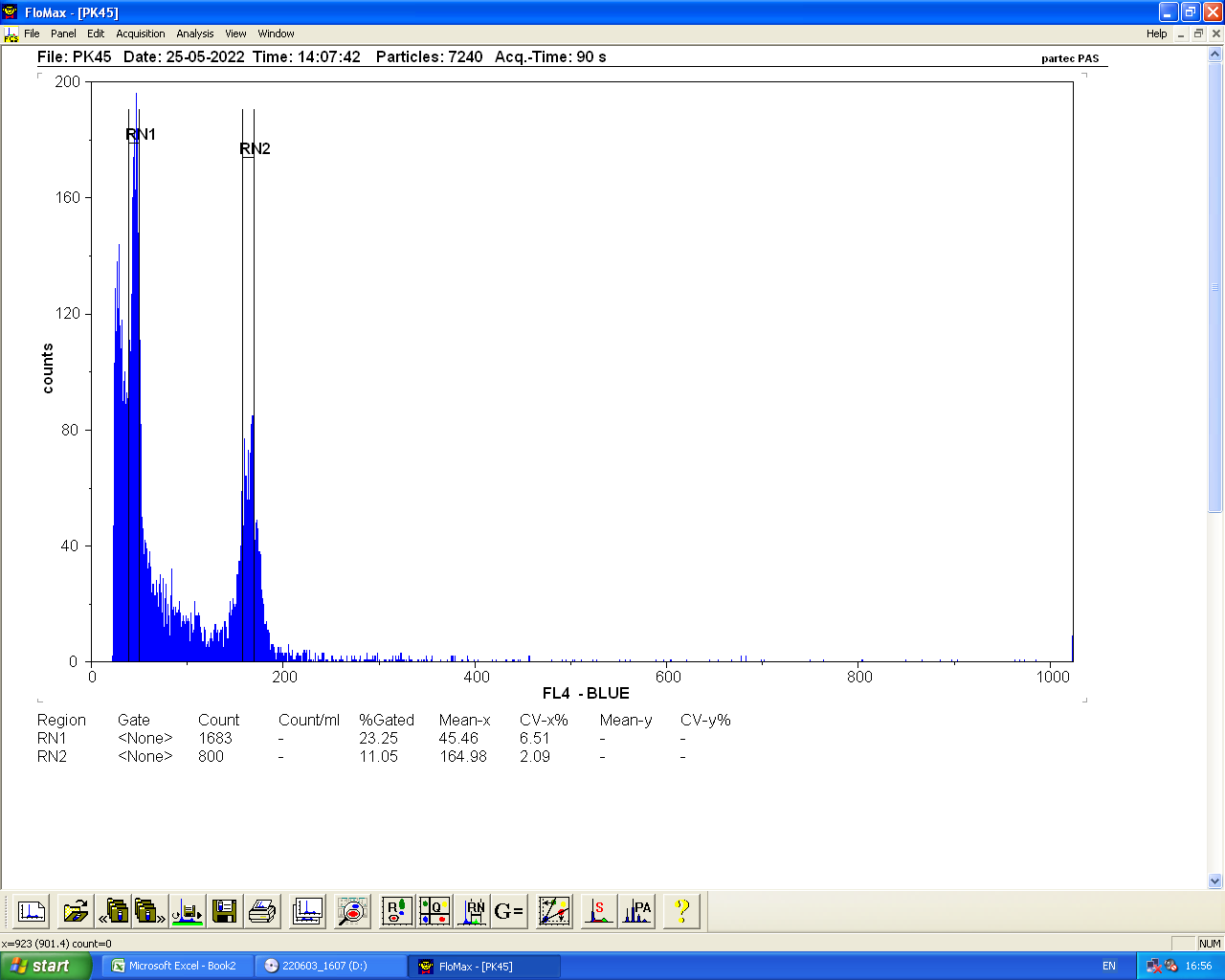 |  |  |  |  |  |  |  |  |
|  |  |  |  |  |  |  |  |  |  |
|  |  |  |  |  |  |  |  |  |  |
| *R. maddenii* ssp. *crassum* | PK59 | 6*x* | *R. fortunei* | fresh | 38.99 | 6.36 | 112.66 | 4.16 | 25-05-2022 (left) |
|  |  | 5*x* | *R. parryae* | fresh | 66.14 | 4.43 | 171.57 | 2.52 | 01-05-2021 (right) |
| 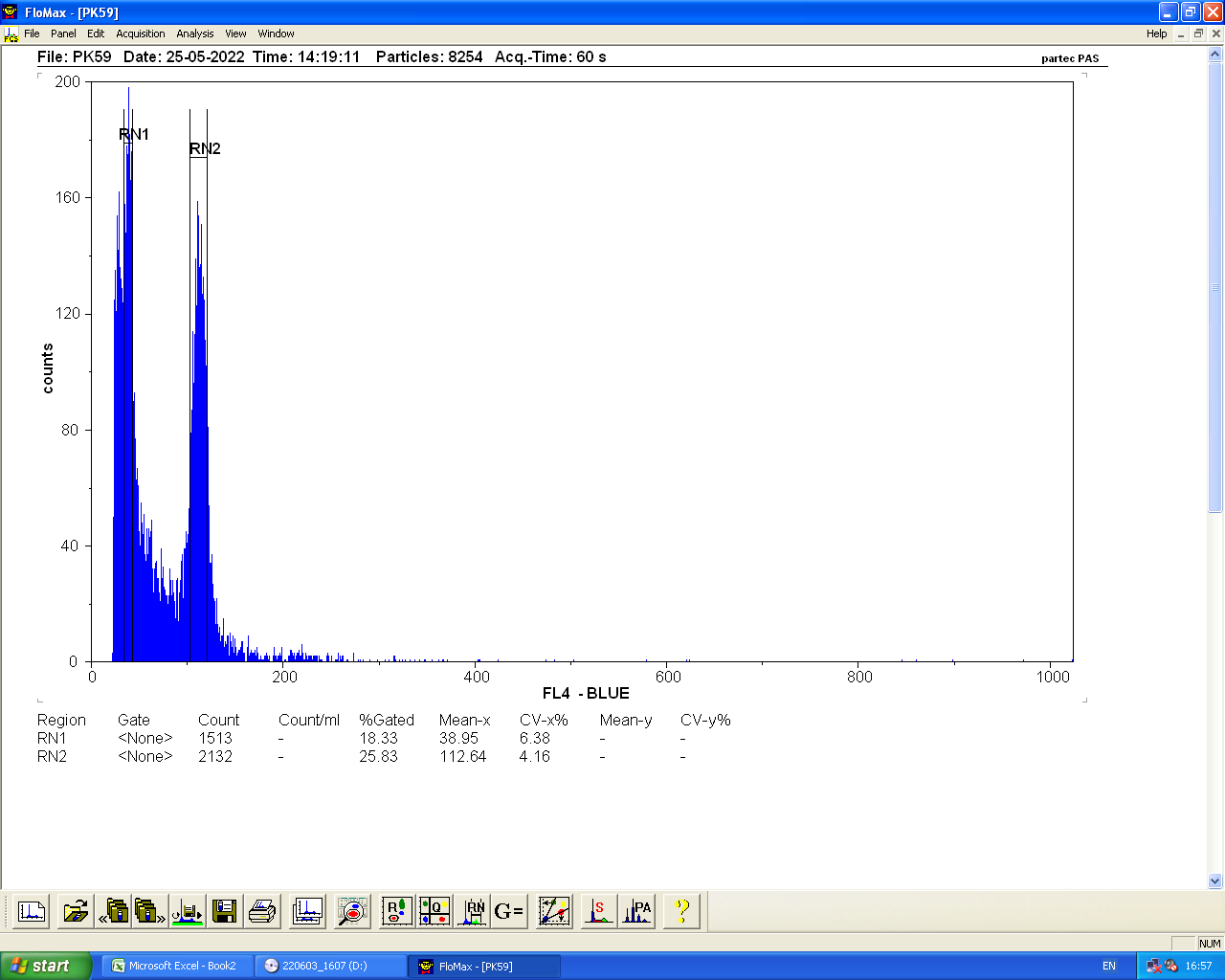 |  |  |  |  |  |  |  |  |  |
|  |  |  |  |  |  | 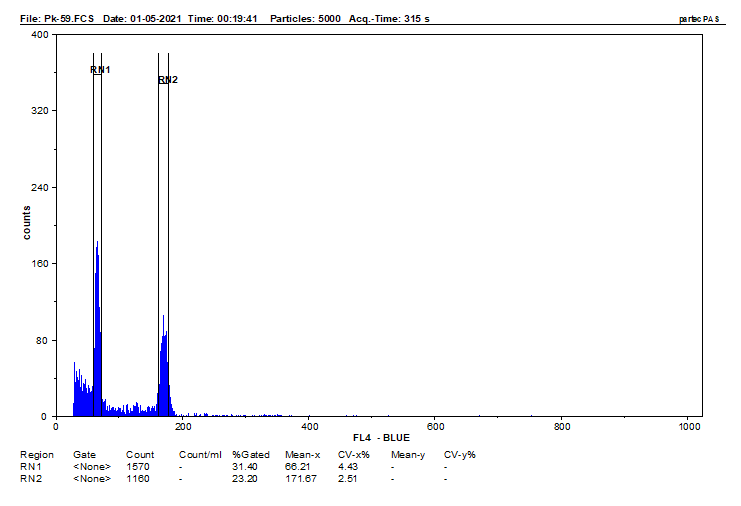 |  |  |  |
|  |  |  |  |  |  |  |  |  |  |
|  |  |  |  |  |  |  |  |  |  |
|  |  |  |  |  |  |  |  |  |  |
|  |  |  |  |  |  |  |  |  |  |
|  |  |  |  |  |  |  |  |  |  |
|  |  |  |  |  |  |  |  |  |  |
|  |  |  |  |  |  |  |  |  |  |
| *R. maddenii* ssp. *crassum* | PK61 | 7*x* | *R. fortunei* | fresh | 45.34 | 6.78 | 157.25 | 3.48 | 25-05-2022 (left) |
|  |  | 6*x* | *R. parryae* | fresh | 63.57 | 5.48 | 197.26 | 2.68 | 30-04-2021 (right) |
|  |  |  |  |  |  |  |  |  |  |
|  |  |  |  |  |  |  |  |  |  |
|  |  |  |  |  |  |  |  |  |  |
|  |  |  |  |  | 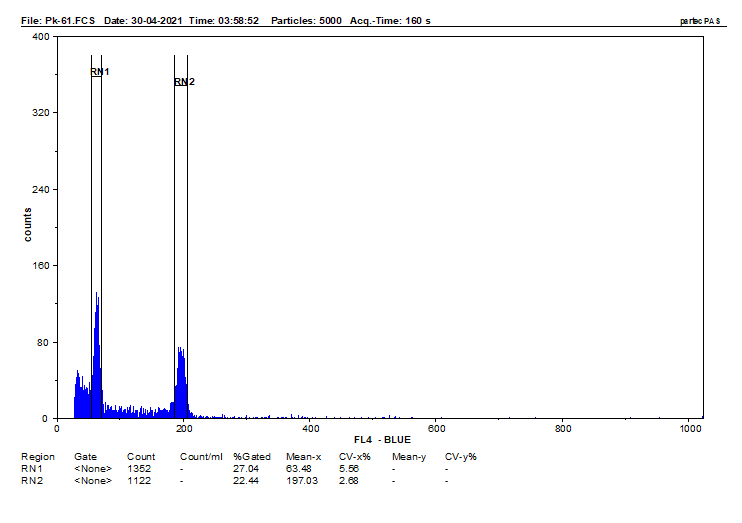 |  |  |  |  |
|  | 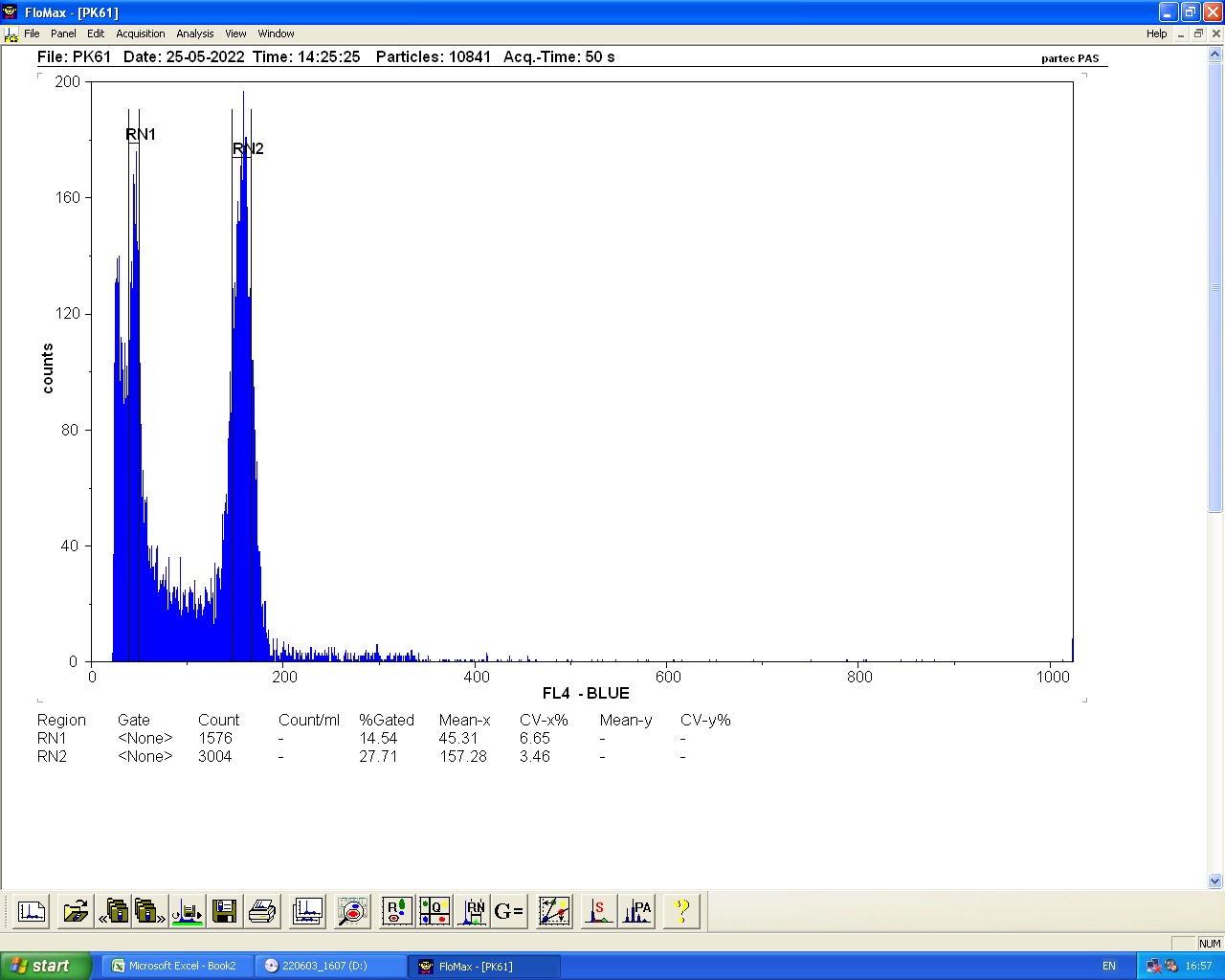 |  |  |  |  |  |  |  |  |
|  |  |  |  |  |  |  |  |  |  |
|  |  |  |  |  |  |  |  |  |  |
| *R. maddenii* ssp. *maddenii* | PK09 | 7*x* | *R. fortunei* | fresh | 41.41 | 16.62 | 151.06 | 2.91 | 25-05-2022 (left) |
|  |  | 5*x* | *R. parryae* | fresh | 61.12 | 5.09 | 163.18 | 2.74 | 30-04-2021 (right) |
| 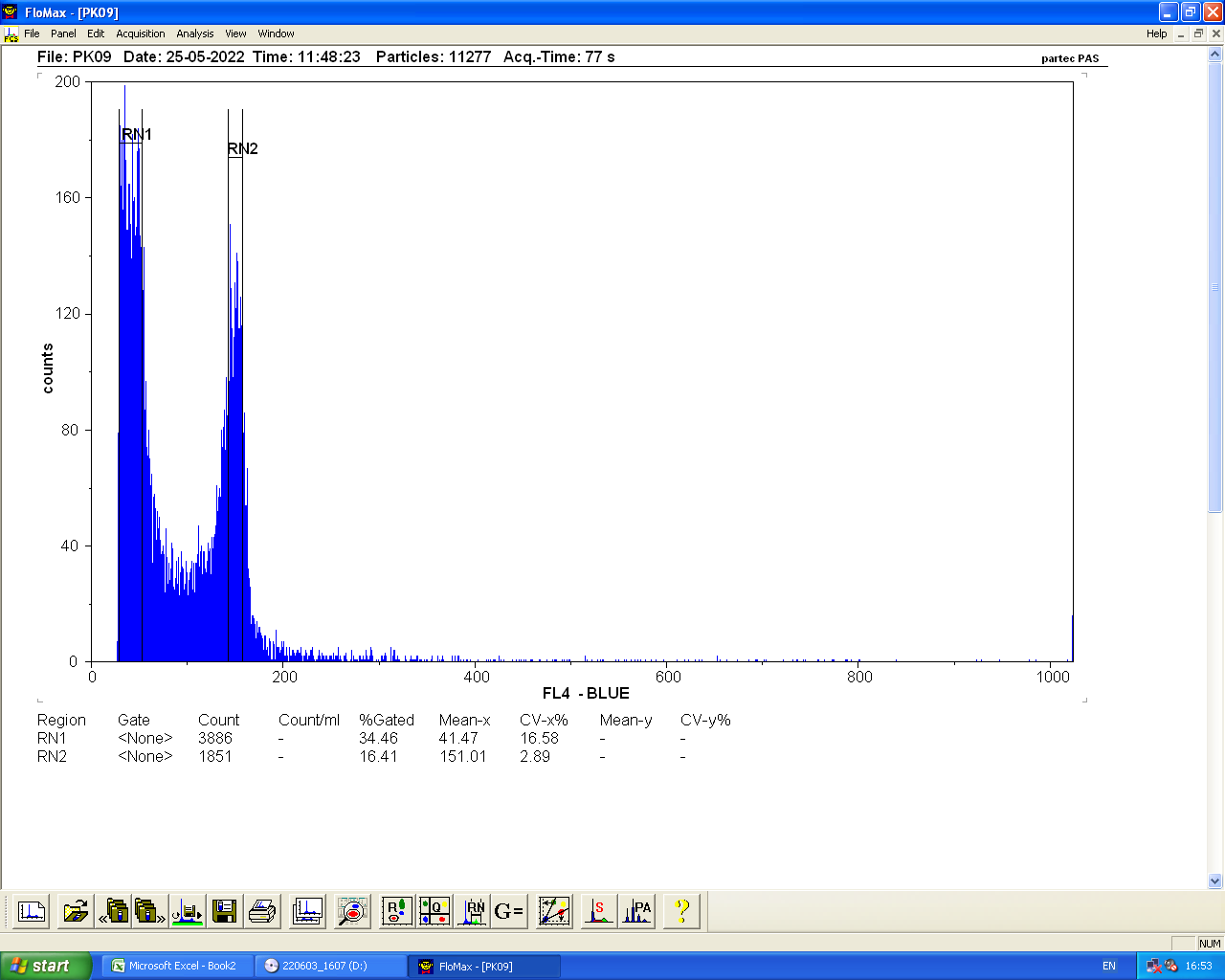 |  |  |  |  |  |  |  |  |  |
|  |  |  |  |  |  |  |  |  |  |
|  |  |  |  |  |  |  |  |  |  |
|  |  |  |  |  |  |  |  |  |  |
|  |  |  |  |  | 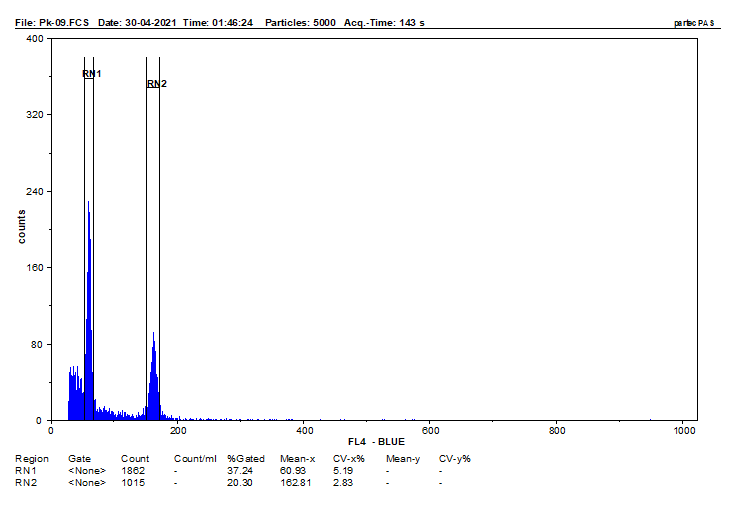 |  |  |  |  |
|  |  |  |  |  |  |  |  |  |  |
|  |  |  |  |  |  |  |  |  |  |
|  |  |  |  |  |  |  |  |  |  |
|  |  |  |  |  |  |  |  |  |  |
| *R. maddenii* ssp. *maddenii* | PK17 | 8*x* | *R. fortunei* | fresh | 43 | 7.02 | 176.66 | 2.57 | 27-05-2022 (left) |
|  |  | 7*x* | *R. parryae* | fresh | 59.74 | 5.13 | 216.78 | 2.24 | 30-04-2021 (right) |
| 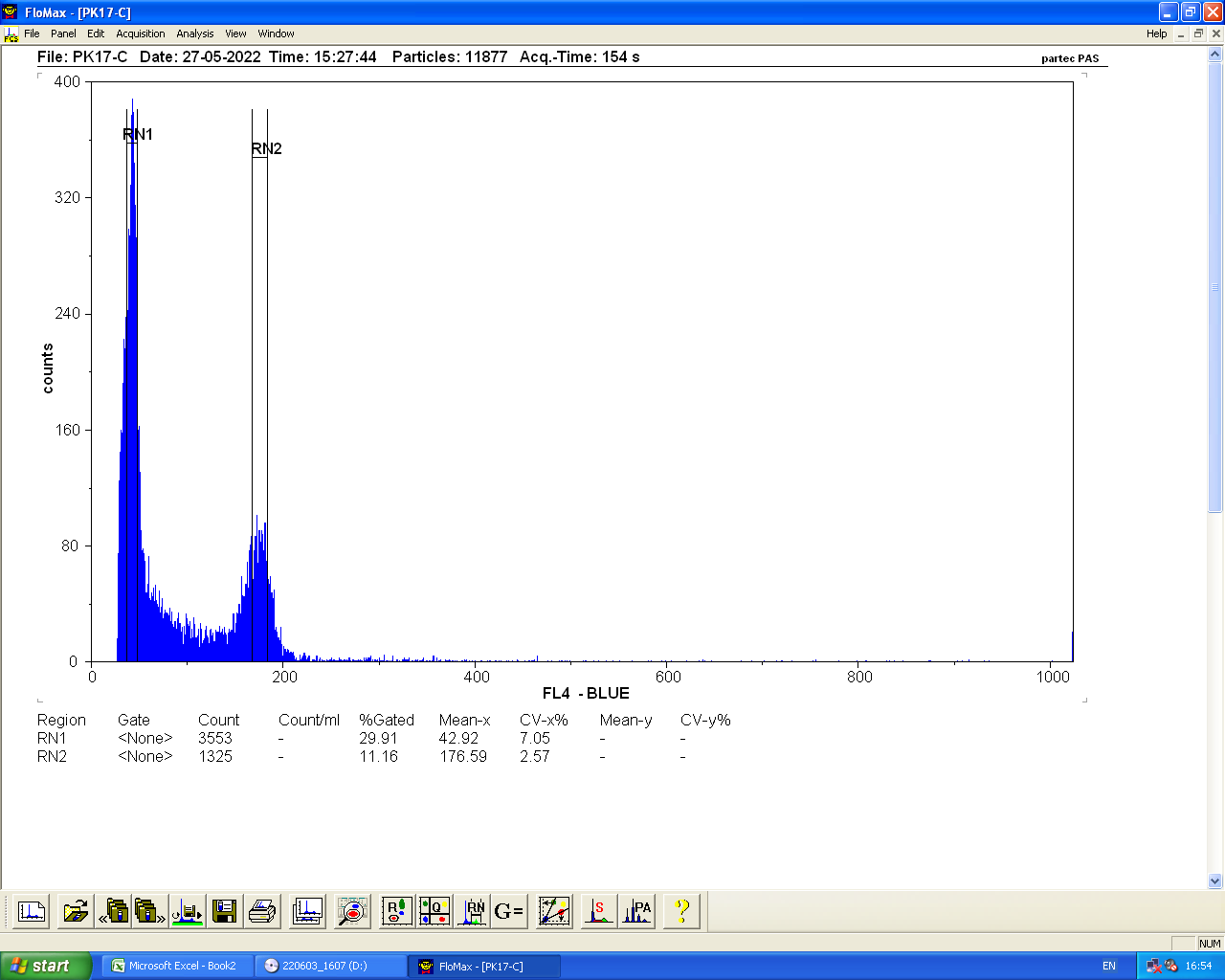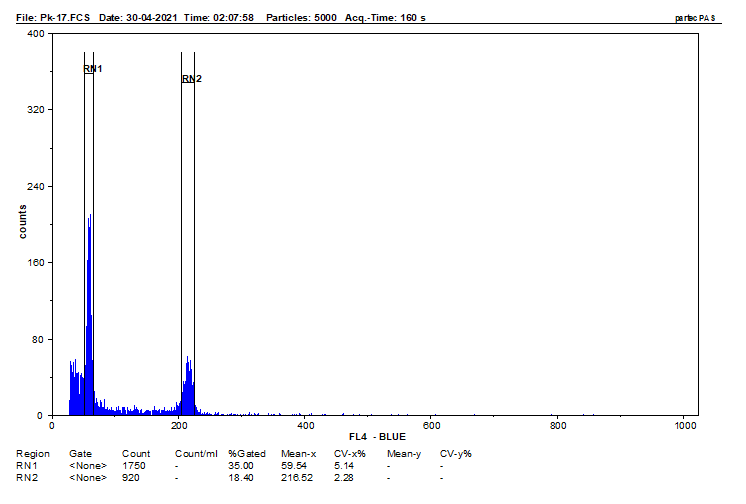 |  |  |  |  |  |  |  |  |  |
|  |  |  |  |  |  |  |  |  |  |
|  |  |  |  |  |  |  |  |  |  |
|  |  |  |  |  |  |  |  |  |  |
|  |  |  |  |  |  |  |  |  |  |
|  |  |  |  |  |  |  |  |  |  |
|  |  |  |  |  |  |  |  |  |  |
|  |  |  |  |  |  |  |  |  |  |
|  |  |  |  |  |  |  |  |  |  |
| *R. maddenii* ssp. *maddenii* | PK27 | 6*x* | *R. fortunei* | fresh | 42.39 | 6.3 | 124.27 | 3 | 25-05-2021 (left) |
|  |  | 5*x* | *R. parryae* | fresh | 56.24 | 6.01 | 146.7 | 2.75 | 30-04-2021 (right) |
| 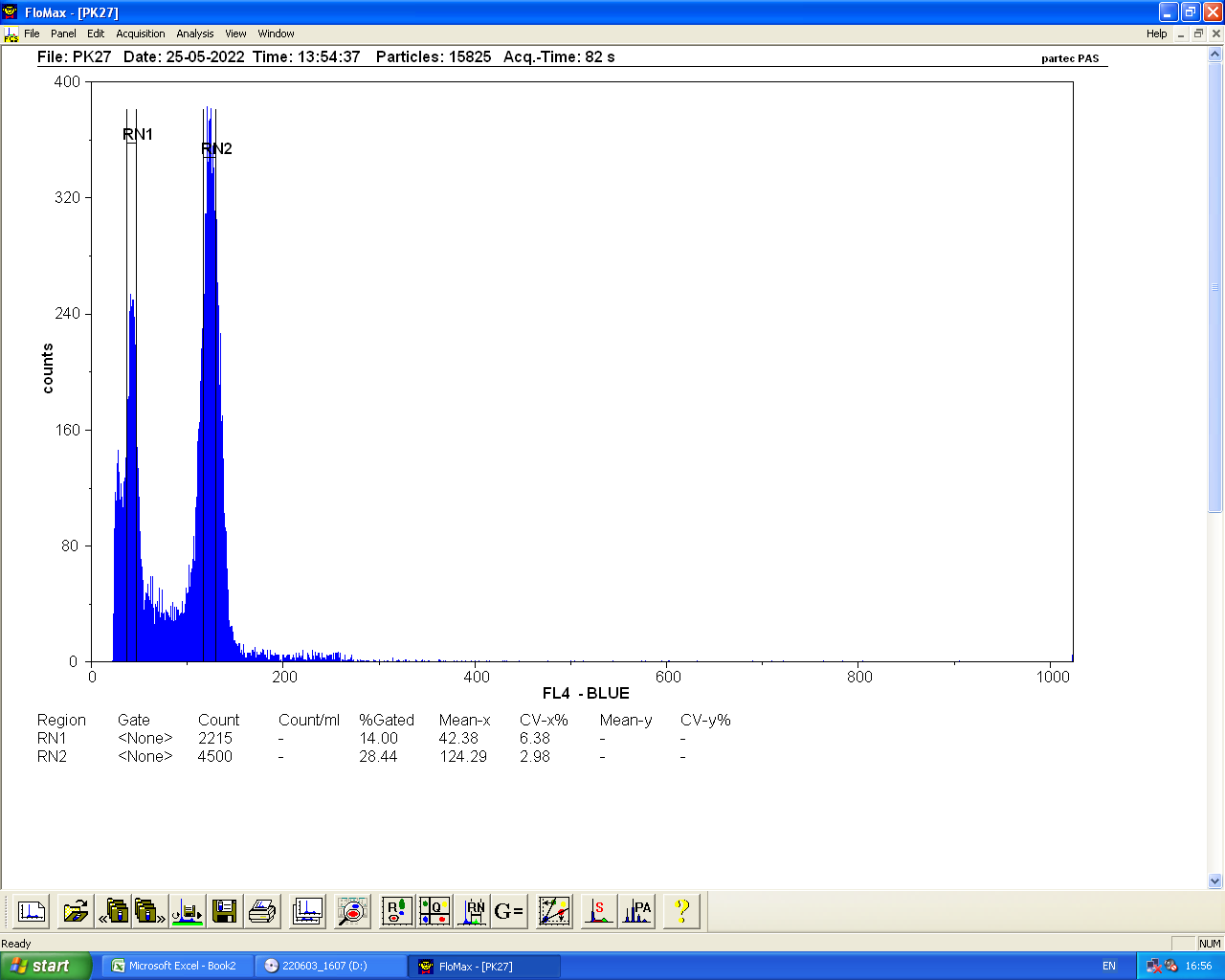 |  |  |  |  |  |  |  |  |  |
|  |  |  |  |  | 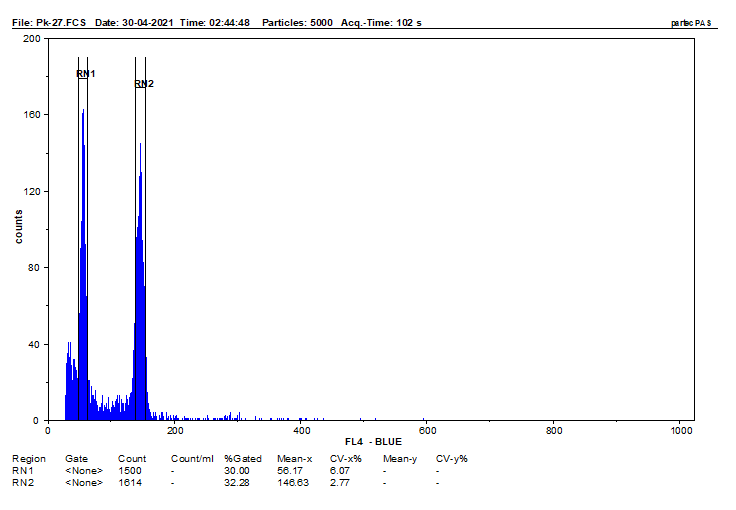 |  |  |  |  |
|  |  |  |  |  |  |  |  |  |  |
|  |  |  |  |  |  |  |  |  |  |
|  |  |  |  |  |  |  |  |  |  |
|  |  |  |  |  |  |  |  |  |  |
|  |  |  |  |  |  |  |  |  |  |
|  |  |  |  |  |  |  |  |  |  |
| *R. maddenii* ssp. *maddenii* | PK38 | 6*x* | *R. fortunei* | fresh | 39.29 | 8.12 | 121.05 | 3.38 | 25-05-2021 (left) |
|  |  | 5*x* | *R. parryae* | fresh | 69.55 | 5.22 | 178.2 | 3.52 | 30-04-2021 (right) |
|  |  |  |  |  |  |  |  |  |  |
|  |  |  |  |  |  |  |  |  |  |
|  |  |  |  |  |  |  |  |  |  |
|  |  |  |  |  |  |  |  |  |  |
|  |  |  |  |  |  |  |  |  |  |
|  |  |  |  |  | 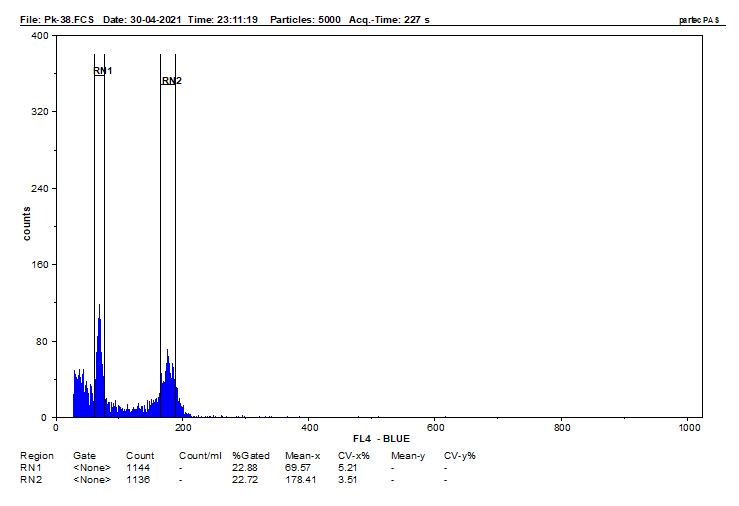 |  |  |  |  |
|  |  |  |  |  |  |  |  |  |  |
| 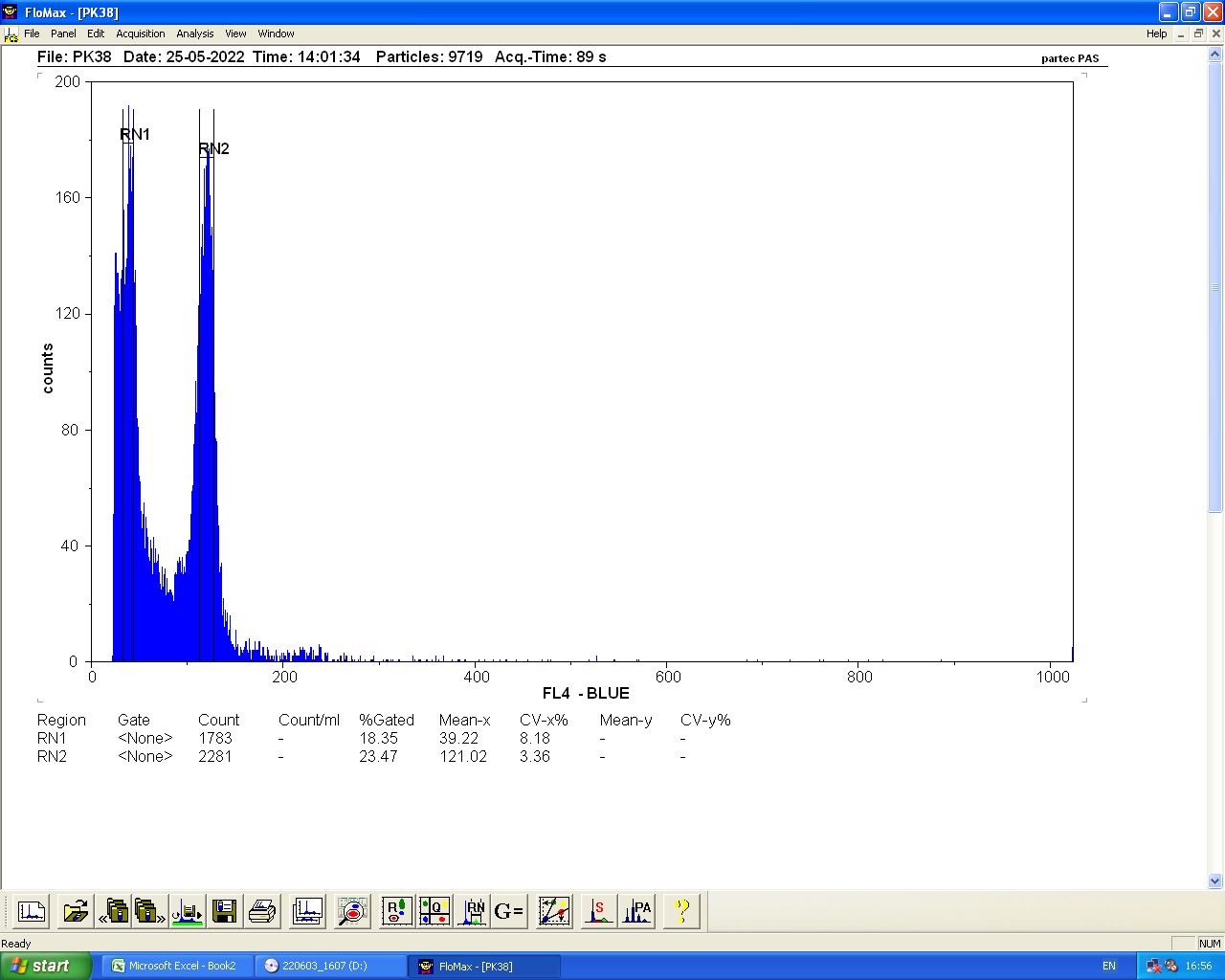 |  |  |  |  |  |  |  |  |  |
| *R. maddenii* ssp. *maddenii* | PK52 | 6*x* | *R. fortunei* | fresh | 42.35 | 6.31 | 127.2 | 2.77 | 25-05-2022 (left) |
|  |  | 5*x* | *R. parryae* | fresh | 68.34 | 4.53 | 181.49 | 2.13 | 01-05-2021 (right) |
| 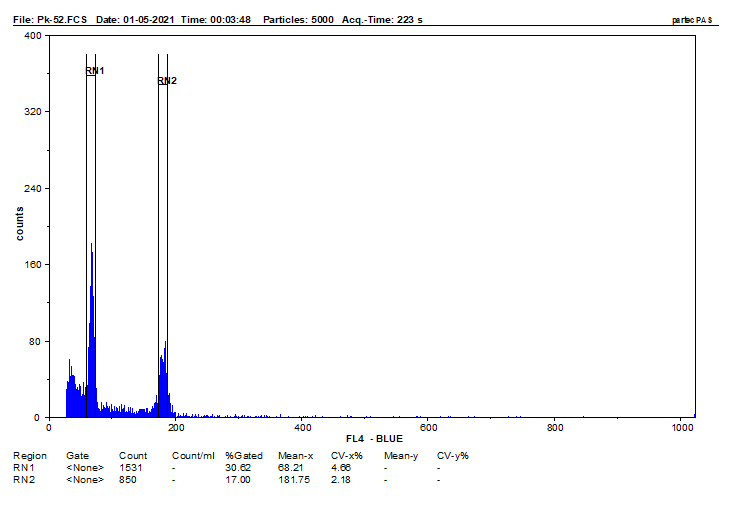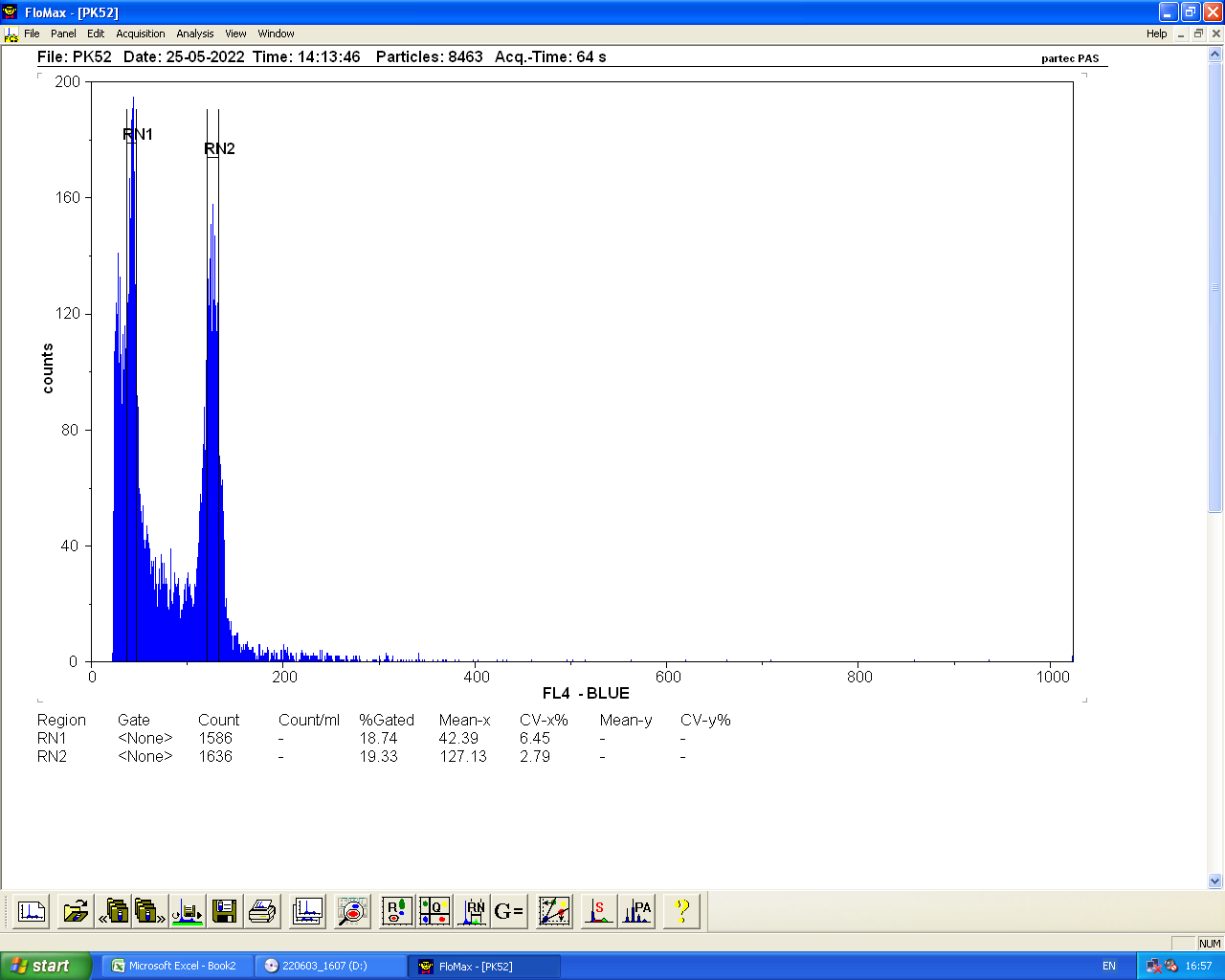 |  |  |  |  |  |  |  |  |  |
|  |  |  |  |  |  |  |  |  |  |
|  |  |  |  |  |  |  |  |  |  |
|  |  |  |  |  |  |  |  |  |  |
|  |  |  |  |  |  |  |  |  |  |
|  |  |  |  |  |  |  |  |  |  |
|  |  |  |  |  |  |  |  |  |  |
|  |  |  |  |  |  |  |  |  |  |
| *R. maddenii* ssp. *maddenii* | PK68 | 6*x* | *R. fortunei* | fresh | 42.92 | 6.96 | 127.69 | 3 | 25-05-2022 (left) |
|  |  | 5*x* | *R. parryae* | fresh | 60.92 | 5.05 | 158.64 | 3.14 | 30-04-2021 (right) |
|  |  |  |  |  |  |  |  |  |  |
|  |  |  |  |  |  |  |  |  |  |
|  |  |  |  |  |  |  |  |  |  |
|  | 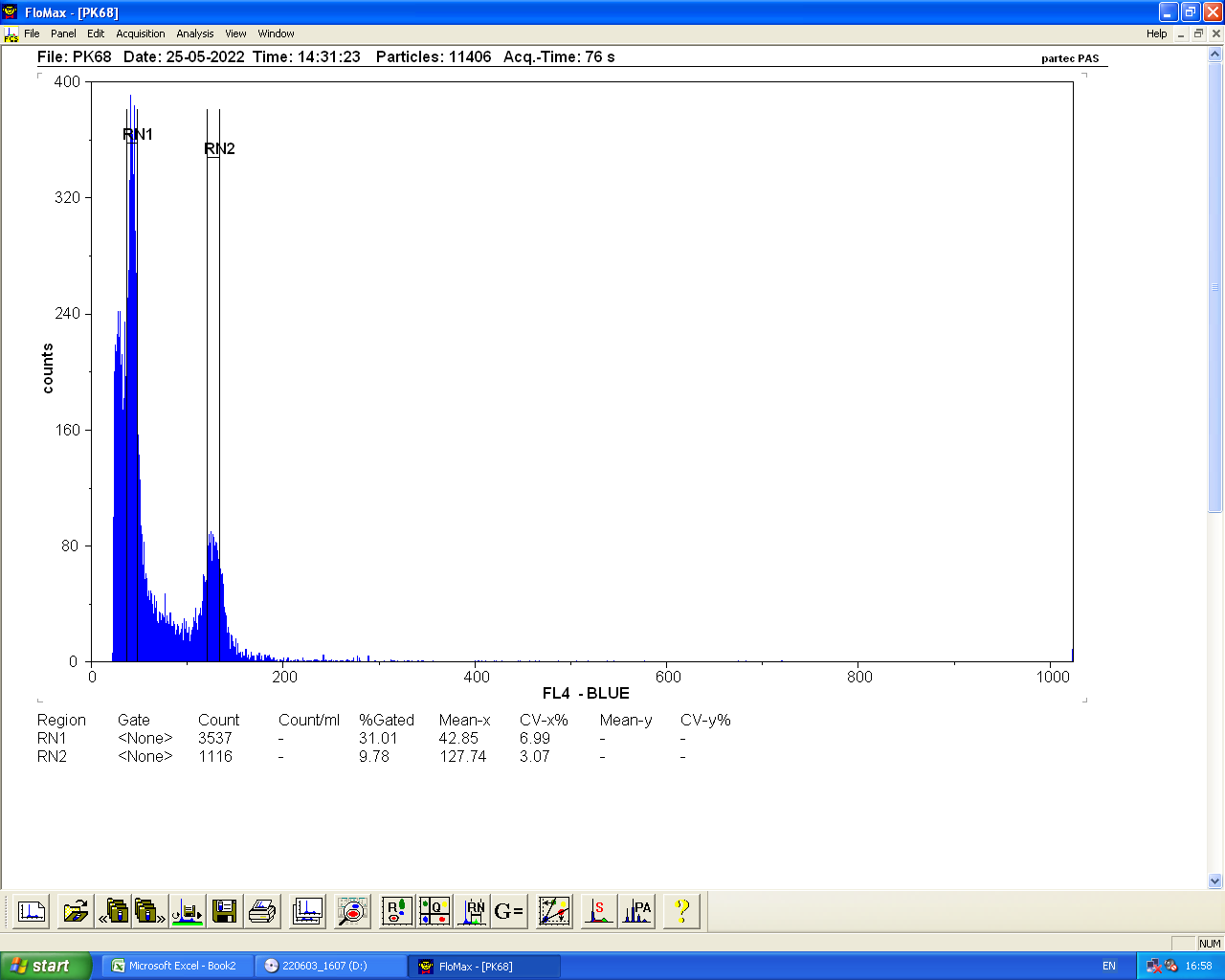 |  |  |  |  |  |  |  |  |
|  |  |  |  |  | 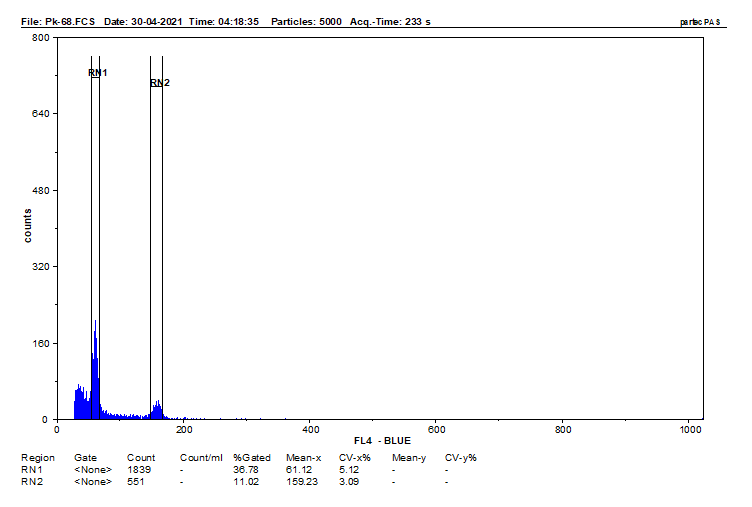 |  |  |  |  |
|  |  |  |  |  |  |  |  |  |  |
|  |  |  |  |  |  |  |  |  |  |
| *R. taggianum* | PK05 | 2*x*? | *R. parryae* | fresh | 54.11 | 7.93 | n/a | n/a | 30-04-2021 (left) |
|  |  |  | no standard co-shopped | fresh | 58.45 | 4.6 | 121.35 | 3.03 | 30-04-2021 (middle)* |
|  |  | 5*x*? | *R. fortunei* | fresh | 47.74 | 8.04 | 129.79 | 4.77 | 17-12-2021 (right) |
|  |  | PK 05 conclusion: 2*x* looks the most reasonable according to ploidy of sample chopped without standard. *showing polyploidy even with no standard material, suspected to be cell cyle of the sample. | | | | |  |  |  |
|  |  |  | | | | |  |  |  |
|  |  |  | | | | |  |  |  |
|  |  |  | | | | |  |  |  |
|  | 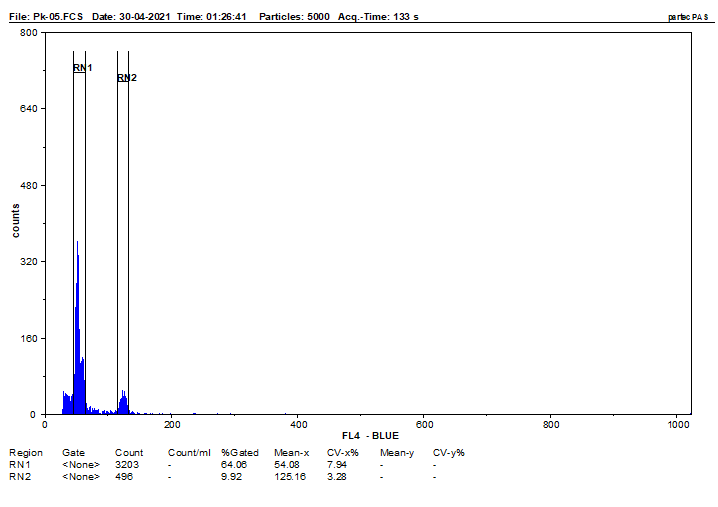 |  | | | | |  |  |  |
|  |  |  | | | | |  |  |  |
|  |  | 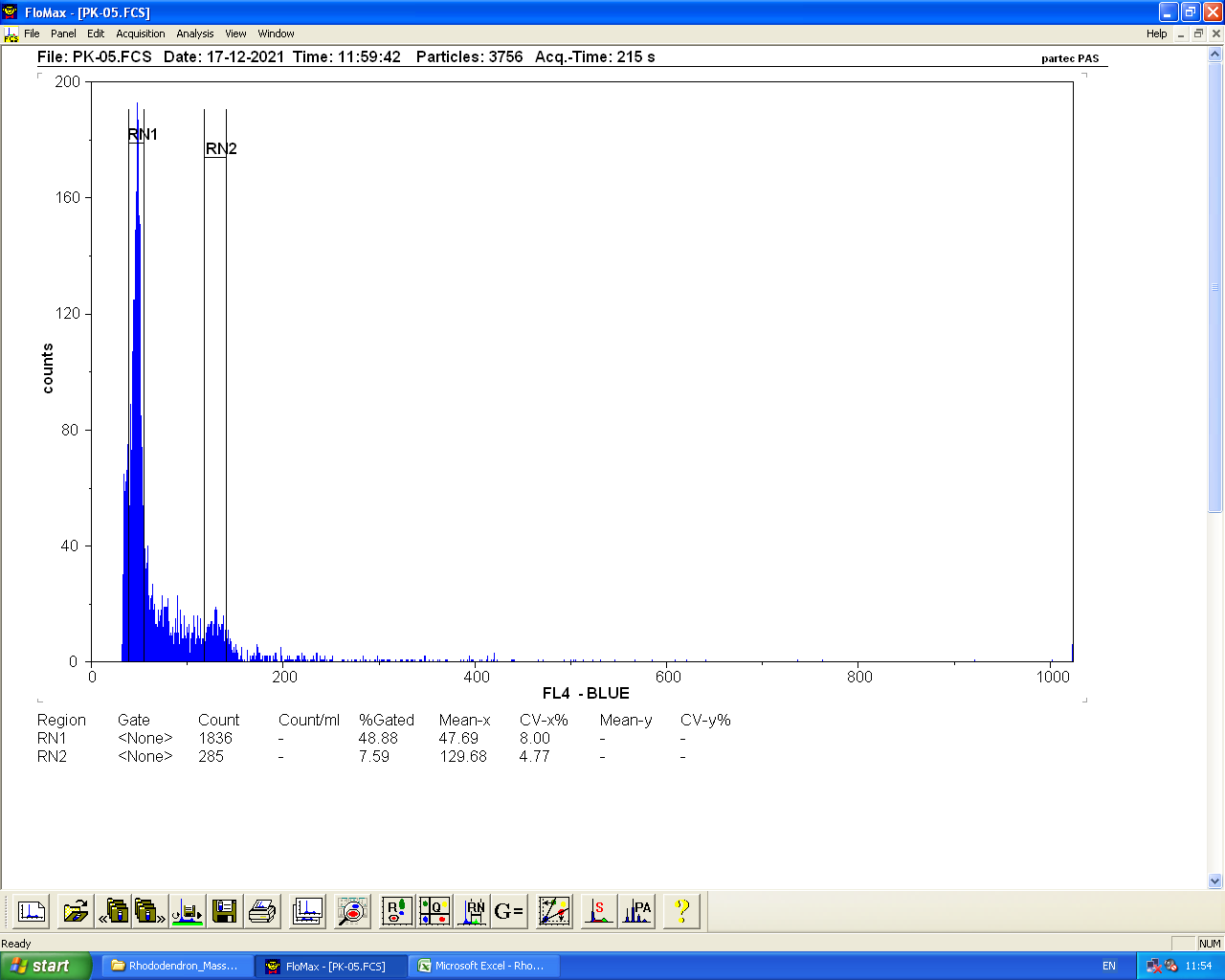 | | | | |  |  |  |


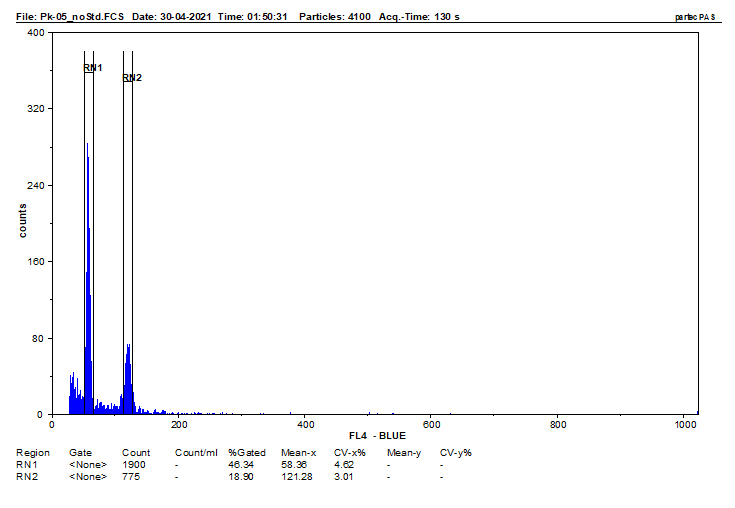

Supplement: plad016_suppl_Supplementary_Table_S3 [file plad016_suppl_supplementary_table_s3.docx]
