# Supplementary material for: Ploidy variation in Rhododendron subsection Maddenia and its implications for conservation
Source: AoB Plants. 2023 Apr 12;15(3):plad016. doi: 10.1093/aobpla/plad016 (PMC10184449; doi:10.1093/aobpla/plad016)
Supplement: plad016_suppl_Supplementary_Table_S4 [file plad016_suppl_supplementary_table_s4.docx]

**Table S4.** Genome size measurements of *R. fortunei* Lindl. and *R. parryae* Hutch. $"2C-Tax"=\frac{"Tax-Mean-x"}{"Std-Mean-x"}\boldsymbol{\times"}2C-Std"$.

| **Taxon** | **Standard** | **Flow Code** | **Tax-Mean-x** | **Std-Mean-x** | **Ratio** | **2C-Std (pg)** | **2C-Tax (pg)** |
| --- | --- | --- | --- | --- | --- | --- | --- |
| *Rhododendron parryae* Hutch. | *Pisum sativum* L. | RP-PS-1 | 71.47 | 377.29 | 0.189429882 | 8.8 | 1.666982957 |
| *Rhododendron parryae* Hutch. | *Pisum sativum* L. | RP-PS-2 | 98.36 | 488.55 | 0.201330468 | 8.8 | 1.771708116 |
| *Rhododendron parryae* Hutch. | *Pisum sativum* L. | RP-PS-3 | 113.82 | 549.49 | 0.207137528 | 8.8 | 1.822810242 |
| ***Rhododendron parryae* Hutch.** |  |  |  |  |  |  | **1.75 (Average)** |
|  |  |  |  |  |  |  |  |
| *Rhododendron parryae* Hutch. | *Zea mays* L. | RP-ZM-1 | 66.16 | 219.54 | 0.301357384 | 5.33 | 1.606234855 |
| *Rhododendron parryae* Hutch. | *Zea mays* L. | RP-ZM-2 | 104.41 | 316.29 | 0.330108445 | 5.33 | 1.759478011 |
| *Rhododendron parryae* Hutch. | *Zea mays* L. | RP-ZM-3 | 103.61 | 317.11 | 0.326732049 | 5.33 | 1.74148182 |
| ***Rhododendron parryae* Hutch.** |  |  |  |  |  |  | **1.70 (Average)** |
|  |  |  |  |  |  |  |  |
| *Rhododendron fortunei* Lindl. (OM60) | *Pisum sativum* L. | RFOM60-PS-1 | 88.15 | 493.78 | 0.178520799 | 8.8 | 1.570983029 |
| *Rhododendron fortunei* Lindl. (OM60) | *Pisum sativum* L. | RFOM60-PS-2 | 85.97 | 503.91 | 0.170605862 | 8.8 | 1.501331587 |
| *Rhododendron fortunei* Lindl. (OM60) | *Pisum sativum* L. | RFOM60-PS-3 | 88.48 | 518.9 | 0.17051455 | 8.8 | 1.50052804 |
| ***Rhododendron fortunei* Lindl. (OM60)** |  |  |  |  |  |  | **1.52 (Average)** |
|  |  |  |  |  |  |  |  |
| *Rhododendron fortunei* Lindl. (OM60) | *Zea mays* L. | RFOM60-ZM-1 | 90.71 | 319.99 | 0.283477609 | 5.33 | 1.510935654 |
| *Rhododendron fortunei* Lindl. (OM60) | *Zea mays* L. | RFOM60-ZM-2 | 90 | 304.61 | 0.295459768 | 5.33 | 1.574800565 |
| *Rhododendron fortunei* Lindl. (OM60) | *Zea mays* L. | RFOM60-ZM-3 | 92.52 | 305.96 | 0.30239247 | 5.33 | 1.611751863 |
| ***Rhododendron fortunei* Lindl. (OM60)** |  |  |  |  |  |  | **1.57 (Average)** |
|  |  |  |  |  |  |  |  |
